# Heterologous biosynthesis and characterization of a glycocin from a thermophilic bacterium

**DOI:** 10.1038/s41467-019-09065-5

**Published:** 2019-03-07

**Authors:** Arnoldas Kaunietis, Andrius Buivydas, Donaldas J. Čitavičius, Oscar P. Kuipers

**Affiliations:** 1 Molecular Genetics Dept., Groningen Biomolecular Sciences and Biotechnology Institute, University of Groningen, Nijenborgh 7, 9747 AG Groningen, Netherlands; 2 Department of Microbiology and Biotechnology, Institute of Biosciences, Life Sciences Center, Vilnius University, Saulėtekio av. 7, LT-10223 Vilnius, Lithuania

## Abstract

The genome of the thermophilic bacterium, *Aeribacillus pallidus* 8, encodes the bacteriocin pallidocin. It belongs to the small class of glycocins and is posttranslationally modified, containing an *S*-linked glucose on a specific Cys residue. In this study, the pallidocin biosynthetic machinery is cloned and expressed in *Escherichia coli* to achieve its full biosynthesis and modification. It targets other thermophilic bacteria with potent activity, demonstrated by a low minimum inhibitory concentration (MIC) value. Moreover, the characterized biosynthetic machinery is employed to produce two other glycopeptides Hyp1 and Hyp2. Pallidocin and Hyp1 exhibit antibacterial activity against closely related thermophilic bacteria and some *Bacillus* sp. strains. Thus, heterologous expression of a glycocin biosynthetic gene cluster including an *S*-glycosyltransferase provides a good tool for production of hypothetical glycocins encoded by various bacterial genomes and allows rapid in vivo screening.

## Abstract (web summary)

Heterologous production of the glycocins, posttranslationally modified peptide bacteriocins containing a sugar moiety, has not been achieved. Here, the authors express a thermophilic bacterium glycocin biosynthetic gene cluster and *S*-glycosyltransferase for the production of antibacterial glycocins in *E. coli*.

## Introduction

Ribosomally synthesized and posttranslationally modified peptides (RiPPs) are produced in all three domains of life. Part of the RiPPs overlap with a group of antibacterial peptides produced by bacteria, and this group historically is designated as bacteriocins^1–3^. They are active against other bacteria that are mostly closely related to the producer. These peptides exhibit considerable diversity with respect to their size, structure, mechanism of action, inhibitory spectrum, immunity mechanisms, and targeted receptors^4^. In the era of emergence of antibiotic-resistant bacteria^5^, bacteriocins have been suggested as a potential alternative to antibiotics in clinics and veterinary settings, but also as food preservatives against spoilage and pathogenic microorganisms^2,6,7^.

Thermophilic bacteria have shown a great potential in biofuel production because of their higher metabolic rate and enzyme stability at elevated temperatures. Moreover, growth at high temperature facilitates recovery of volatile products, like ethanol^8^, and reduces requirement for cooling. Thermophilic fermentations are less prone to contaminations by mesophiles, although there are still risks that bioreactors will be contaminated by other thermophiles^9,10^. In addition, contamination by thermophiles is also a problem in production of dairy products^11^. This shows the need of discovery of new natural compounds that have activity against thermophilic bacteria.

Glycocins are posttranslationally glycosylated bacteriocins. The sugar moieties are linked to the side chains of either Cys, Ser, or Thr residues. A glycocin can be regarded as being “glycoactive” when sugar moieties are essential for the antimicrobial activity^12^. Only a few glycocins have been identified and reported to date: sublancin 168, produced by *Bacillus subtilis*^13^; glycocin F, produced by *Lactobacillus plantarum*^14^; ASM1 (homologous to glycocin F), produced by *Lb. plantarum*^12,15^; enterocin F4-9, produced by *Enterococcus faecalis*^16^ and thurandacin, encoded by *Bacillus thuringiensis* and identified by genomic data mining and chemo-enzymatical synthesis in vitro^16^.

The understanding of their mechanism of growth inhibition of target bacteria is far from complete. It is known that a specific phosphoenolpyruvate:sugar phosphotransferase system (PTS) is a factor affecting glycocin F and sublancin antibacterial activities^12,17^ and that sublancin does not affect the integrity of the cell membrane and acts bacteriocidal^12,17^. In contrast, glycocin F and enterocin F4-9 have been reported to be bacteriostatic^14,16^.

The synthetic machinery of the best-studied glycocin, sublancin, is encoded by a gene cluster containing *sunA, sunS, sunT, bdbA*, and *bdbB* genes. The precursor peptide, SunA, is modified by the *S*-glycosyltransferase SunS, which forms a very unusual *S*-linkage between the Cys residue and glucose. SunA glycosylation in vitro by SunS has been confirmed by chemo-enzymatical synthesis of mature sublancin^13^. Based on the SunT sequence similarity to bacteriocin ABC- transporters/peptidases, it is assumed that SunT cleaves the leader sequence and transports the core peptide to the outside of the cell. Two thiol-disulfide oxidoreductases, BdbA and BdbB, might be responsible for disulfide bond formation in the peptide^18,19^. In addition, it has been confirmed that the same gene cluster encodes the immunity protein SunI^20^. A similar genetic organization was found in gene clusters encoding the putative synthetic machineries of glycocin F^14^, thurandacin^21^ and enterocin F4-9^16^.

To date only two bacteriocins, i.e. geobacillin I and geobacillin II, produced by the thermophilic bacteria *Geobacillus thermodenitrificans* NG80-2, are well characterized^22–24^. Other bacteriocin-like antibacterial compounds from thermophilic microorganisms have been described in much less detail^25–31^. These reasons prompted us to find and to study new bacteriocins of this group. Thus, we have chosen the thermophilic *Aeribacillus pallidus* 8 strain that was previously isolated from soil above oil wells in Lithuania^32^. Previous studies have shown that this strain secretes an antibacterial compound that is active against other thermophilic bacteria. Unfortunately purification of this compound and identification of its amino acid sequence were not successful^32,33^.

In this study we have identified genes in the genome of *A. pallidus* 8 that encode a biosynthetic machinery for a hypothetical bacteriocin (i.e. pallidocin), which belongs to the small class of glycocins. We also demonstrate the functional expression of the whole biosynthetic gene cluster of a glycocin in Gram-negative *Escherichia coli*, which facilitates further engineering and mechanistic studies.

Following characterization of pallidocin demonstrated that it exhibits extremely strong activity against specific thermophilic bacteria, such as *(Para)Geobacillus* sp. and *Caldibacillus* sp. In addition, we identified and synthesized a variety of hypothetical glycopeptides and determined their properties by employing heterologous expression system of pallidocin. The characterized pallidocin *S*-glycosyltransferase PalS could be used not only for biosynthesis of hypothetical glycocins, but also for introduction of unique posttranslational modifications into other peptides with the aim to improve their bioactivities. Pallidocin is a good candidate to prevent bacterial contaminations in industrial fermentations operating under elevated temperatures.

Previously, full maturation of recombinant glycocins was only reported in vitro for thurandacin and sublancin. Glycosylation and leader cleavage were performed enzymatically, followed by chemical oxidative folding^13,21^. The in vitro experiments limit the yield of the final product, are time consuming and expensive. Recently, a system was developed for the heterologous expression of sublancin in *E. coli* SHuffle T7 Express cells that in vivo installs the glycosylation and oxidative folding following a single in vitro step of proteolytic leader cleavage^34^. The SHuffle T7 Express strain expresses the disulfide bond isomerase DsbC, aiding oxidative folding of proteins in the cytoplasm^35^. Here, we demonstrate a different in vivo heterologous expression system to produce completely mature glycocins in *E. coli* BL21(DE3), evading the in vitro chemical and enzymatic steps.

## Results

### Identification and heterologous biosynthesis of pallidocin

**Fig. 1:**
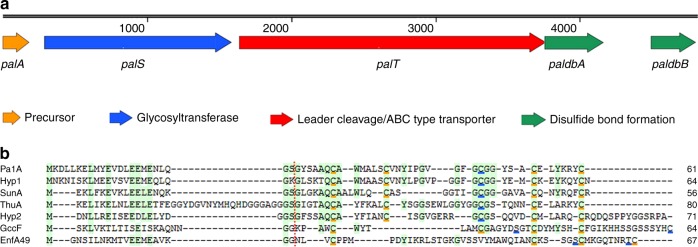
Gene cluster and precursor peptides of glycocins. **a** Pallidocin biosynthetic gene cluster (4805 bp) *pal* identified by BAGEL4 in *A. pallidus* 8 genome. **b** Alignment of glycocin precursor peptides. Conserved regions are highlighted in green color, Cys forming disulfide bonds are underlined in orange, glycosylated amino acids are underlined in blue. Red dots between amino acids indicate predicted or experimentally determined leader cleavage sites. Numbers in the end of the sequences indicate the length of peptides in amino acids. PalA pallidocin precursor, SunA sublancin precursor, Hyp putative glycocin precursor Hyp1, Hyp2 putative glycocin precursor Hyp2, EnfA4-9 enterocin F4-9 precursor, GccF glycocin F precursor

The pallidocin producer strain was identified as *A*. *pallidus* 8 (previously referred to as *Geobacillus* sp. 8^32^) by a Genome-to-Genome Distance Calculator (GGDC) using a digital DNA−DNA hybridization (dDDH) analysis tool^36^. The *A. pallidus* 8 genome^37^ was processed with the BAGEL4 bioinformatics tool^38^ for the identification of gene clusters for bacteriocins biosynthesis. An operon coding for a putative glycocin was identified (Fig. 1a). It contains five genes which were named *palA*, *palS*, *palT*, *paldbA*, and *paldbB*. Proteins encoded by these genes display between 38 and 53% sequence similarity with proteins of known functions, encoded in other bacteria. Based on this analysis we presumed that *palA* encodes the 61 amino acid precursor peptide (Fig. 1b), *palS* encodes the SunS-like family peptide *S*-glycosyltransferase, *palT* encodes the SunT-like superfamily peptidase/ABC transporter protein, *paldbA* encodes the thioredoxin-like enzyme, and *paldbB* encodes the DsbB-like disulfide bond formation protein B.

We decided to try to express this gene cluster in the easy-to-handle Gram-negative host *E. coli*. The whole biosynthetic gene cluster (*pal*) of the hypothetical glycocin (Fig. 1a), which was named pallidocin, was amplified by PCR and cloned into the expression vector (Supplementary Fig. 1). The *pal* operon, starting with the start codon for the PalA, was cloned into the MCS behind the arabinose promoter and RBS of the vector. The expression of the *pal* genes in the heterologous host *E. coli* BL21(DE3) was induced with arabinose. The produced active antibacterial peptide was purified from 2L of supernatant (Supplementary Fig. 2) using liquid chromatography methods. The yield of the peptide was enough for mass spectrometric (MS) analysis and initial antibacterial activity screening, but not for the quantification by measuring the absorbance at 280 nm wavelength.

### Structural and functional characterization of pallidocin

The predicted monoisotopic mass [M + H]^+^ of the unmodified pallidocin core peptide is 4061.76. The monoisotopic mass [M + H]^+^ of purified native pallidocin observed by LC-ESI-MS was 4219.79 (Supplementary Fig. 3a). The monoisotopic mass [M + H]^+^ of the peptide, after treatment with tris(2-carboxyethyl)phosphine-hydrochloride (TCEP), was 4223.82 (Supplementary Fig. 3b). This suggests that the peptide has a posttranslational modification with a mass of +162 and two disulfide bonds (−4). To identify the modified residue, pallidocin was fragmented with chymotrypsin and further analyzed by LC-ESI-Q-MS/MS mass spectrometry (Supplementary Figs. 4-9). This showed that Cys25 of the core peptide has a modification with a mass of +162.05, which might be a hexose moiety. To identify the sugar, pallidocin was analyzed by GC-MS (Supplementary Fig. 10). The results demonstrated that the moiety attached to the Cys25 residue is glucose.

**Fig. 2:**
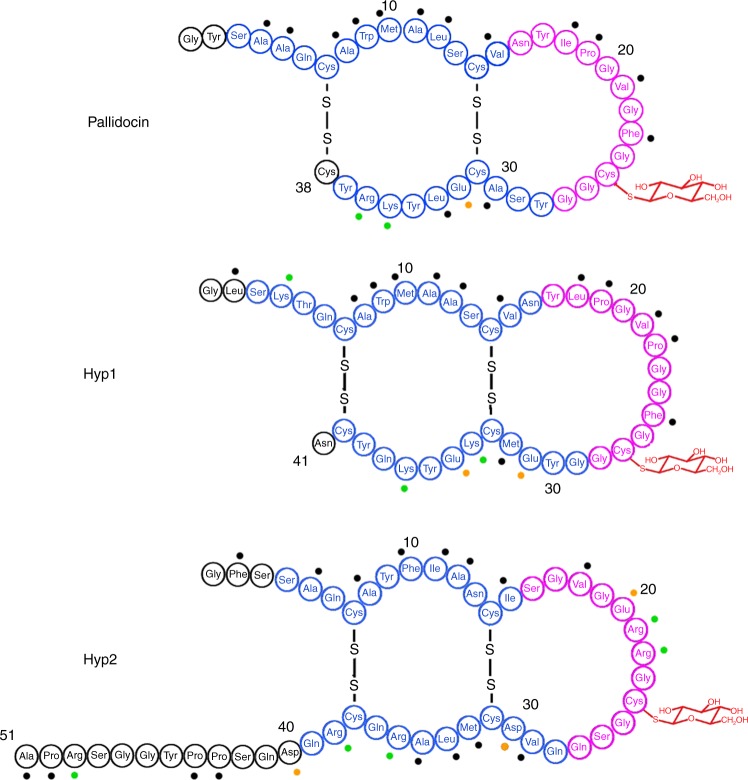
Proposed structures of pallidocin, Hyp1 and Hyp2 glycocins based on PSIPRED predictions from the sequence and the known tertiary structure of sublancin 168 and GccF. α-helical structure highlighted in blue color. Coil structure highlighted in purple. Black dots indicate hydrophobic amino acids. Orange indicates acidic amino acids, whereas green indicates basic amino acids

Based on the analysis by the secondary structure prediction tool PSIPRED^39^, pallidocin has two α-helices (Fig. 2). Far-UV CD spectra analysis of mature pallidocin (Supplementary Fig. 11) revealed that the peptide contains substantial amounts of helical structure, judging from the pattern of the spectra from 193 to 240 nm. Similar spectra patterns were observed for sublancin^34,40,41^ and glycocin F^14^, also. The estimate of the secondary structure content, made by the method of Raussens et al.^42^, predicted predominantly helical structure, with an estimate of 47% helix. The peptide was also estimated to contain 13% β-turn and 11% β-sheet structure.

A wide range of temperatures and pHs were applied to evaluate pallidocin’s stability (Supplementary Table 1). The peptide was stable at room temperature for 10 days, while after 30 days it retained 12% of the activity. Fifty percent antibacterial activity was retained after autoclaving pallidocin at 121 °C for 15 min. Incubation at the pH range 2−10 did not affect pallidocin’s antibacterial activity, demonstrating its exceptionally high stability.

### Functional assessment of the genes *palS* and *palT*

The genes *palS* and *palT* were cloned (Supplementary Fig. 12) and coexpressed with *palA-his* in *E. coli* BL21(DE3) to determine their functions. Gene *palA* was fused with a His7-tag in the C-terminus (Supplementary Figs. 12 and 13) to facilitate the purification of its product. The highest yield of synthesized peptides was observed in the insoluble fraction of the cell lysate. Peptides were purified from the insoluble fraction, tested for activity and analyzed by MALDI-TOF-MS to evaluate the presence of modifications (Table 1).

**Table 1:** Results of glycocin precursor genes coexpression with *palS* and *palT*

Coexpressed genes	*palA-his*	*sunA-his*	*hyp1-his*	*hyp2-his*	*enfA4-9-his*	*gccF-his*
No coexpression (only precursor peptide)	NA, NM, NLC	NA, NM, NLC	NA, NM, NLC	NA, NM, NLC	NA, NM, NLC	NA, NM, NLC
Coexpression with palS	+, Glc, NLC	+, Glc, NLC	+, Glc, NLC	NA, Glc, NLC	NA, NM, NLC	NA, NM, NLC
Coexpression with palT	NA, NM, leader cleaved	NA, NM, NLC	NA, NM, NLC	NA, NM, NLC	NA, NM, NLC	NA, NM, NLC
Coexpression with palST	+, Glc, leader cleaved	+, Glc, NLC	+, Glc, NLC	NA, Glc, NLC	NA, NM, NLC	NA, NM, NLC

Glc refers to the peptide’s modification with a mass of +162; plus symbol refers to the presence of antibacterial activity of the purified peptide

*NA* no activity, *NM* no modifications, *NLC* no leader cleavage, *palA-his* pallidocin precursor, *sunA-his* sublancin precursor, *hyp1-his* putative glycocin precursor Hyp1, *hyp2-his* putative glycocin precursor Hyp2, *enfA4-9-his* enterocin F4-9 precursor, *gccF-his* glycocin F precursor

**Fig. 3:**
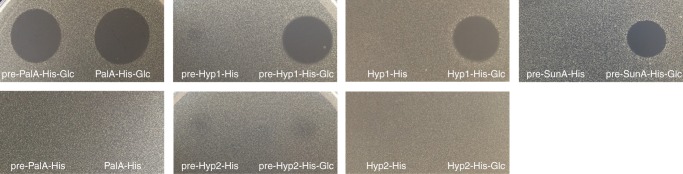
Antibacterial activities of synthesized and purified peptides. The peptides were derived after coexpression of glycocin precursors with PalS/PalT/PalST proteins in *E. coli*, subsequent purification by Ni^2+^ affinity chromatography (IMAC) and RP-HPLC. Antibacterial activity was indicated as a clearly visible inhibition zone of sensitive strain *P. genomospecies* 1 NUB36187 and assessed by a spot on a lawn assay

As expected, expression of only *palA-his* resulted in the synthesis of pre-PalA-His, the precursor peptide with the leader still attached (Supplementary Fig. 14), and with no antibacterial activity (Fig. 3). Coexpression of *palA*-*his* and *palS* genes resulted in the biosynthesis of pre-PalA-His-Glc (the precursor peptide with the leader attached and a mass increment of 162), which portrays glucosylation (Supplementary Fig. 15). Notably, this compound was active against the sensitive thermophilic strain *Parageobacillus genomospecies* 1 NUB36187 (Fig. 3). Coexpression of *palA*-*his* with *palT* resulted in the biosynthesis of the PalA-His core peptide with a mass corresponding to the leaderless peptide and this compound lacked antibacterial activity (Fig. 3). Coexpression of the genes *palA*-*his* with *palS* and *palT* resulted in the biosynthesis of a PalA-His-Glc core peptide (mature pallidocin) with the mass corresponding to the leaderless peptide with a mass increment of 162, corresponding to glucosylation (Supplementary Fig. 16). The glucosylated core peptide had antibacterial activity too (Fig. 3).

### The role of disulfide bonds on the antibacterial activity

To confirm the presence and importance of disulfide bonds in pallidocin, purified glycosylated precursor peptide with leader pre-PalA-His-Glc and leaderless PalA-His-Glc core peptide (mature pallidocin) were treated with TCEP and iodoacetamide (IAA) (Supplementary Table 2). These peptides were derived after coexpression of *palA-his* with *palS* or *palST*. After the treatment, the masses of pre-PalA-His-Glc and PalA-His-Glc core peptide measured by MALDI-TOF-MS increased by +234 and +228, respectively. The expected mass increment for one alkylated Cys is 57, for four alkylated Cys is 228. After all, despite the observed mass difference (6), which is tolerated as the machine error, it is clear that the disulfide bonds were reduced and all free Cys residues were alkylated.

Notably, the disruption of disulfide bonds resulted in the loss of antibacterial activity against the indicator strain *P*. *genomospecies* 1 NUB36187 (Supplementary Table 2). After treatment with only IAA, no loss of antibacterial activity and mass increment was observed (Supplementary Table 2), confirming that all disulfide bonds are present in the pre-PalA-His-Glc and the PalA-His-Glc core peptide.

### In vitro leader peptide cleavage of pallidocin precursor

To develop a more efficient way for pallidocin production, the genes *his-Xa-palA* and *palS* were coexpressed in *E. coli* BL21(DE3). PalA was engineered by adding a His-tag at the N-terminus and a Factor Xa peptidase cleavage site (IEGR) in front of the core peptide (Supplementary Fig. 13) for convenient leader removal. After coexpression, the glycosylated precursor peptide pre-His-Xa-PalA-Glc was purified and the leader was cleaved off in vitro using Factor Xa. The generated leaderless PalA-Glc core peptide (mature pallidocin) was further purified by RP-HPLC followed by mass spectrometry analysis for mass confirmation (Supplementary Fig. 17). The yield of synthesized active mature pallidocin was ~15 μg per 100 mL of bacterial culture. Activities of the glycosylated precursor peptide with leader and mature pallidocin were compared by the agar well diffusion assay using *P. genomospecies* 1 NUB36187 as a sensitive strain (Supplementary Fig. 18). The assay shows the highest serial twofold dilution of bacteriocin sample, which still displays antibacterial activity. Results indicate that pre-His-Xa-PalA-Glc had approximately 500 times lower activity than the mature pallidocin.

### Pallidocin alignment with other glycocins

BLAST analysis of PalA identified two hypothetical peptides (Hyp1 and Hyp2) which have low sequence similarity to PalA (Fig. 1b). They were encoded in *Bacillus megaterium* BHG1.1 (Hyp1) and *Bacillus* sp. JCM19047 (Hyp2) genomes. Hyp1 consists of a 23-residue leader sequence, a 41-residue core peptide, a 20-residue leader sequence Hyp2, and a 51-residue core peptide. Each of the peptides Hyp1 and Hyp2 has five Cys residues in the core sequence.

**Fig. 4:**
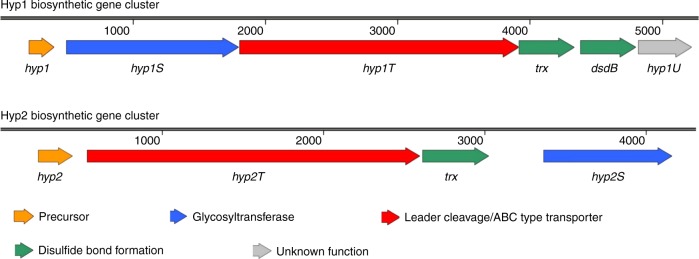
The predicted biosynthetic gene clusters of glycocins Hyp1 and Hyp2. Hyp1 biosynthetic gene cluster encoded in the genome of *B. megaterium* BHG1.1 is 5013 bp in length and encodes for proteins: Hyp1, Hyp1S, Hyp1T, Trx, DsbB, and Hyp1U. The Hyp2 biosynthetic gene cluster encoded in the genome of *Bacillus* sp. JCM19047 is 3932 bp in length and encodes for proteins: Hyp2, Hyp2T, Trx, and Hyp2S

Genome analyses of *B. megaterium* BHG1.1 and *Bacillus* sp. JCM19047 by the BAGEL4 tool did not find any gene clusters related to bacteriocin biosynthesis. However, BLASTp analysis of the genomic context of *hyp1* revealed a gene cluster coding for putative glycocin biosynthetic machinery (Fig. 4). Genes in the cluster alongside the Hyp1 precursor gene encode for: Hyp1S protein with 50% sequence similarity to SunS-like family peptide *S*-glycosyltransferases; Hyp1T protein with 68% sequence similarity to SunT-like superfamily peptidase domain-containing ABC transporters; Trx protein with 69% sequence similarity to thioredoxin-like superfamily proteins and DsbB protein with 74% sequence similarity to DsbB-like superfamily disulfide bond formation proteins B. Moreover, BLASTp analysis of the genomic context of *hyp2* revealed that a gene cluster may encode for putative glycocin biosynthetic machinery (Fig. 4). Genes in the cluster alongside the Hyp2 precursor gene encode the Hyp2S protein with 42% sequence similarity to SunS-like family peptide *S*-glycosyltransferases; Hyp2T protein with 40% sequence similarity to SunT-like superfamily peptidase domain-containing ABC transporters and the Trx protein with 43% sequence similarity to thioredoxin-like superfamily proteins. Two putative glycocin precursors, i.e. Hyp1 and Hyp2, were investigated and examined further for possible posttranslational modifications by the biosynthetic machinery of pallidocin.

Alignment of all glycocins (Fig. 1b) showed that the leader sequences of SunA, ThuA, PalA, and Hyp1 share motifs of conserved residues. In contrast, the leader sequences of GccF and EnfA49 display no significant similarities. All glycocins have a double-glycine type motif (a proteolytic cleavage site at the end of the leader sequence). GccF and EnfA49 precursors have leader cleavage sites following the Gly-Gly motif. SunA, ThuA, and PalA precursors have a Gly-Ser-Gly motif, where the leader sequence is cleaved between the Ser and second Gly residue. In case of peptide Hyp2, it has putative leader cleavage site with motif Gly-Ser-Gly, whereas peptide Hyp1 has Gly-Lys-Gly.

All glycocins characterized to date have five Cys residues in the core peptide and form two disulfide bonds between them. Hyp1 and Hyp2 core peptides have also five Cys residues and it is very probable that they form these bonds too. Early studies and our current one show that the Cys residue in the conserved region Gly-Cys-Gly-Gly/Ser of SunA, ThuA and PalA is glucosylated. This motif is present in Hyp1 and Hyp2 peptides as well and it suggests that Cys in this region is the target for glycosylation.

The secondary structure prediction tool PSIPRED^39^ proposes that two α-helices are present in the Hyp1 and Hyp2 core peptides as well as in pallidocin. Four out of five Cys residues reside in these helical structures of the peptides. Structures of sublancin 168 and glycocin F elucidated by NMR method have also two α-helices, which are nested by two disulfide bonds^43,44^ and giving rise to the predicted structures for Hyp1 and Hyp2 (Fig. 2).

### Heterologous biosynthesis of hypothetical glycocins

To determine whether PalS and PalT are able to modify and process different heterologous glycocin precursors in a heterologous Gram-negative host, they were coexpressed with genes coding for glycocin precursor peptides with leaders and His6-tags (Supplementary Figs. 13 and 19), i.e. pre-SunA-His, pre-GccF-His, pre-EnfA49-His, pre-Hyp1-His and pre-Hyp2-His. As before, the highest yield of peptides was observed in the insoluble fraction of cell lysate. After coexpression, the peptides were purified from the insoluble fraction and analyzed by mass spectrometry (Table 1). The analysis could not confirm that PalT cleaved off the leaders. However, it demonstrated that in addition to pre-PalA-His, the PalS glycosyltransferase does modify pre-SunA-His (Supplementary Fig. 20 and 21), pre-Hyp1-His (Supplementary Figs. 22 and 23), and pre-Hyp2-His (Supplementary Fig. 24 and 25), with a mass of +162 consistently pointing to monoglycosylation. The antibacterial activity analysis of the peptides showed that only pre-SunA-His-Glc and pre-Hyp1-His-Glc (glycosylated precursors with leaders) but not the pre-Hyp2-His-Glc were active against indicator strain *P. genomospecies* 1 NUB36187 (Table 1 and Fig. 3).

We designed the genes *core_hyp1-his* and *core_hyp2-his* encoding the leaderless Hyp1 and Hyp2 core peptides with a His6-tag sequence at the C-terminus (Supplementary Figs. 13 and 19). As previously, *palS* was coexpressed with the *core_hyp1-his* and *core_hyp2-his*. The peptides were produced, purified and analyzed by mass spectrometry. LC-ESI-MS analysis confirmed that leaderless Hyp1-His and Hyp2-His core peptides (Supplementary Figs. 26 and 27) were glycosylated. However, only the Hyp1-His-Glc core peptide showed antibacterial activity against *P. genomospecies* 1 NUB36187 (Fig. 3). To confirm the importance of glycosylation, *core_hyp1-his* gene was expressed in *E. coli* without *palS* coexpression. The recombinant Hyp1-His core peptide did not show any antibacterial activity (Fig. 3), indicating that glycosylation indeed plays a crucial role in the antimicrobial activity of Hyp1, as well as for pallidocin, pre-SunA-His-Glc (this work) or mature sublancin^13,34^ (glucosylated and oxidatively folded SunA core peptide). In addition, reduced and alkylated pre-Hyp1-His-Glc (Supplementary Table 3) and Hyp1-His-Glc core peptides (Supplementary Table 4) lost their antibacterial activities, confirming that disulfide bonds are also important for glycocin activity.

### Antibacterial activity of identified glycocins

The antibacterial activity spectrum of purified pallidocin was tested against a number of Gram-positive and Gram-negative bacteria using a spot on lawn assay. Purified pallidocin exhibited antibacterial activity against *Bacillus cereus* ATCC14579, *B. megaterium* DSM319 and some thermophilic bacteria: *Geobacillus* *stearothermophilus* B4109, B4111, B4112, B4114 strains, *P. genomospecies* 1 NUB36187, *Parageobacillus toebii* B4110, B4162 strains, *Parageobacillus caldoxylosilyticus* B4119 and *Caldibacillus debilis* B4165.

The activity spectrum of the synthesized glycocin Hyp1-His-Glc (glycosylated leaderless core peptide) was partially similar to the pallidocin spectrum (Supplementary Fig. 28). Glycocin was active against *Geobacillus* sp. B4113 and *G. stearothermophilus* B4163 strains, but exhibited no activity against *G. stearothermophilus* B4112, *P. toebii* B4110, *P. caldoxylosilyticus* B4119 and *B. cereus* ATCC14579 strains, in contrast to pallidocin.

Minimum inhibitory concentrations (MICs) were determined for some pallidocin susceptible strains (Supplementary Tables 5, 6 and 7). In liquid NB medium, the MIC of pallidocin against *B. megaterium* DSM319 was 37 nM, *P. genomospecies* 1 NUB36187 was 2.4 pM (i.e ±10 ng/L), *G. stearothermophilus* B4114 was 246 pM, *Geobacillus toebii* B4162 was 493 pM, and *P. caldoxylosilyticus* B4119 was 985 pM. MIC tests showed that *Geobacillus* spp. strains used in this assay rapidly acquired resistance for pallidocin as observed by growing colonies in the halo area or the growth in some 96-plate wells with pallidocin concentrations higher than MIC.

## Discussion

We identified and characterized a posttranslationally modified bacteriocin (pallidocin). The gene cluster of the pallidocin biosynthetic machinery is encoded on the chromosome of the thermophilic bacterium *A. pallidus* 8, which is unprecedented. The characterized bacteriocin belongs to the small class of glycocins and shares genetic and structural similarities with sublancin 168^13^, glycocin F^14^, thurandacin^21^ and enterocin F4-9^16^.

Here, we show that the whole glycocin biosynthetic gene cluster, derived from a thermophilic bacterium, can be cloned and functionally expressed in a heterologous host *E. coli* BL21(DE3). Surprisingly, mature bacteriocin (glycosylated, oxidatively folded and leaderless) from Gram-positive bacteria could be synthesized and secreted by this Gram-negative host. Structural characterization of the purified recombinant pallidocin revealed posttranslational modifications: glycosylation and two disulfide bonds within two predicted α-helices. These two features appear to be specific for the class of glycocins^12^. Pallidocin has an *S*-linked glucose moiety to the Cys25, which is very uncommon among bacteria. Only a few cases of *S*-linked glycopeptides have been described and confirmed to date, i.e. for glycocin F, sublancin 168 and thurandacin^13,14,21^.

Pallidocin exhibits high stability after exposure to high temperatures and a wide range of pH values. To our knowledge, only sublancin and enterocin F4-9 have been properly characterized for their stability. The stability of sublancin is decreased by 50% after 30 min incubation at 70 °C temperature. Sublancin is not very stable at acidic conditions; after incubation at pH 2 and 3 for 30 min, it retains only 20 and 40% of its activity, respectively^40^, while enterocin F4-9 after incubation at 80 °C for 15 min retains its full activity only at pH values from 2 to 8. After incubation at 100 °C for 15 min, enterocin F4-9 retains its full activity only at pH 4. Its activity is completely lost after incubation at 121 °C as well as at pH 10^16^. Compared to sublancin and enterocin F4-9, pallidocin is much more stable at high temperatures. Its activity decreases 50% only after 15 min incubation at 121 °C and is completely stable at 90 °C for 3 h. In contrast to sublancin and enterocin F4-9, pallidocin retains its full activity at acidic and basic conditions (pH 2−10).

In vitro studies on the glycosylation of the sublancin precursor showed that *S*-glycosyltransferase has relaxed substrate specificity. It is able to attach other sugars: xylose, mannose, *N*-acetylglucosamine or galactose, as well. The native glycopeptide sublancin purified from *B. subtilis* contains glucose^13^. We do not know which sugar would be present in native pallidocin if the peptide was derived from *A. pallidus* 8. We can assume that native pallidocin has an *S*-linked glucose as well, as this sugar was found in recombinant pallidocin produced by *E. coli*.

Experiments in which only two or three genes were coexpressed revealed that *palS* codes for an *S*-glycosyltransferase, which introduces glucose to the Cys25 residue in pallidocin. When *palA-his* was coexpressed with *palS* and *palT*, the mature pallidocin (PalA-His-Glc core peptide) was purified also. Additionally, the PalT protein shares sequence similarity to other bacteriocin ABC transporters, which leads to the conclusion that PalT has a dual, i.e. peptidase and transport, function.

Oxidative folding of the peptide in the cytoplasm is unlikely. The formation of structural disulfide bonds in *E. coli* appears to be strictly segregated according to subcellular compartmentalization^45^. Because a reducing environment is necessary for enzymatic activity of glycosyltransferases^13^ most probably PalS glycosylates peptides in the cytoplasm. When the whole gene cluster *pal* is expressed, the synthesized glycosylated precursor peptide should be transported to the periplasm by PalT, where the oxidative folding could take place. Following the precursor peptide coexpression with PalS, these bonds could be formed spontaneously by air oxidation^46,47^ during peptide extraction and/or the purification process. In case of precursor peptide coexpression with PalST, disulfide bonds in the glycosylated core peptide could be formed in the periplasm or spontaneously by air oxidation^46,47^ during the peptide extraction and purification process.

We applied PalS for possible modifications of other glycocins and produced two glycosylated peptides: Hyp1 and Hyp2. PalS specifically monoglycosylated a certain group of glycocin precursors (pre-PalA-His, pre-SunA-His, pre-Hyp1-His, and pre-Hyp2-His), but it was not able to modify pre-GccF-His and pre-EnfA4-9-His. Interestingly, the 42%, 50% and 53% sequence similarities of Hyp1S, Hyp2S and SunS, respectively, to the PalS *S*-glycosyltransferase show quite surprisingly that this similarity is enough to allow modification of heterologous substrates.

The distinctive feature of the glucosylated peptides is a Cys residue flanked by glycines (Gly-Cys-Gly-Gly/Ser) in the interhelical loop. PalS, ThuS, and SunS form a sugar *S*-linkage with a Cys residue in Gly-Cys-Gly-Gly/Ser motif of interhelical loop, and this motif is present only in SunA, ThuA, PalA, Hyp1, and Hyp2. Therefore, we can assign PalA, Hyp1, and Hyp2 to the *sublancin-*type glycocins, which is composed of SunA and ThrA^12^.

Previous research on the sublancin *S*-glycosyltransferase, SunS, suggested that this enzyme recognizes an α-helical segment of the substrate and glycosylates only a Cys residue in the flexible loop following this helix^48^. Studies on thurandacin glycosyltransferase, ThuS, showed that it glycosylates ThuA at Cys28 or both Ser19 and Cys28. ThuS represents glycosyltransferase that catalyzes both *O*- and *S*-glycosylation of proteins. Earlier studies also demonstrated that SunS is not able to modify ThuA, although ThuS is able to modify SunA generating its bisglucosylated product. Moreover, SunA and ThuA with changed short sequences in their interhelical loops were also glucosylated by ThuS in these regions. On basis of this knowledge, it was suggested that the peptide sequence selectivity of ThuS is much more relaxed than that of SunS^21^. Here, we demonstrate that PalS has quite flexible substrate selectivity too and that it may monoglycosylate various precursor peptides: pre-PalA-His, pre-SunA-His, pre-Hyp1-His, and pre-Hyp2-His. In addition, we show that leaderless Hyp1-His and Hyp2-His core peptides can be modified by PalS, resulting in highly active antibacterial peptide Hyp1-His-Glc but not Hyp2-His-Glc.

All sublancin-type glycocins, including the pallidocin, Hyp1 and glycosylated core peptide Hyp2, have a relatively rich content of hydrophobic residues at the N-terminus, and charged residues at the C-terminus. Comparing the core peptides of glycocins, Hyp2 has a relatively long C-terminus “tail”, not characteristic to other sublancin-type glycocins, and is relatively rich in charged residues (Glu20, Arg21, Arg22) in the interhelical loop (Fig. 2). These two features or one of them might be the reason why glycosylated Hyp2 core peptide and precursor did not have antibacterial activity against the strains tested. This could be the subject for future research on glycocin variability.

In contrast to previous work on sublancin^48^, a newly published study showed that nonglycosylated and oxidatively folded core peptide of sublancin has the same topology of disulfide bonds as the native sublancin^34^. In fact, the previous assumptions that the free thiol of unmodified Cys disrupts the formation of the correct disulfide bridges by thiol-disulfide exchange and the blocked Cys residue can aid to form correct disulfide bonds between four free Cys residues^48^ were proven wrong.

We show that the disruption of disulfide bonds in the glycosylated precursor peptides pre-PalA-His-Glc, pre-Hyp1-His-Glc or the PalA-His-Glc and Hyp1-His-Glc core peptides leads to the loss of antibacterial activity. These data support observations of earlier investigations on sublancin. It confirms that disulfide bonds are crucial for antibacterial activity of glycocins as well as glycosylation^13,34^.

Earlier studies on sublancin and thurandacin showed that the leader must be cleaved off to gain antibacterial activity^13,21^. In contrast, our synthesized glycosylated precursors with leaders (pre-PalA-His-Glc, pre-SunA-His-Glc and pre-Hyp1-His-Glc) still showed activity against a sensitive strain, suggesting that the leader removal from the glycosylated glycocin precursor is not essential for activity. In fact, glycocins with a leader attached exhibit substantial antibacterial activity. However, the absence of the leader increases the activity. It should be noted that in contrast to previous studies on glycocins, we have used a thermophilic indicator strain, which, as we show, exhibits extreme susceptibility, even to a leader-containing glycocin.

Previously, only full maturation of recombinant glycocins was reported in vitro for thurandacin and sublancin. Glycosylation and leader cleavage was performed enzymatically, followed by chemical oxidative folding^13,21^. The in vitro experiments limit the yield of the end product, are time consuming and expensive. Recently, a system was developed for the heterologous expression of sublancin in *E. coli* SHuffle T7 Express cells that in vivo installs the glycosylation and oxidative folding following a single in vitro step of proteolytic leader cleavage^34^. SHuffle T7 Express strain expresses the disulfide bond isomerase DsbC, aiding oxidative folding of proteins in the cytoplasm^35^. Here, we demonstrate a different in vivo heterologous expression system for completely mature glycocins in *E. coli* BL21(DE3), evading the in vitro chemical and enzymatic steps.

With the aid of PalS and PalT we can synthesize completely mature and active pallidocin, which is glycosylated, oxidatively folded and leaderless. Because pallidocin glycosyltransferase has flexible substrate selectivity, we propose that PalS could be a good tool for in vivo biosynthesis and screening of hypothetical glycocins, as we showed with Hyp1 and Hyp2. This approach demonstrates that after in vivo peptide glycosylation the disulfide bonds most probably are formed spontaneously during the purification process. It means that the in vitro chemical oxidative folding is not absolutely necessary. Moreover, the in vivo glycosylation of core peptides evades the enzymatic leader cleavage.

Pallidocin exhibits antimicrobial activity against specific Gram-positive bacteria. Most of the tested thermophilic bacteria were susceptible to pallidocin. Among *Bacillus* sp. only *B. megaterium* DSM319 and *B. cereus* ATCC14579 strains were susceptible. It indicates a rather narrow activity spectrum restricted to closely related bacteria. Enterocin F4-9 exhibits antimicrobial activity against *Enterococcus faecalis* and *E. coli* JM109^16^. Glycocin F has narrow activity spectrum also that is restricted to the *Lactobacillus* genus^14^. Notably, sublancin is active against several Gram-positive bacteria, like *Staphylococcus* and, especially *Bacillus* species^20^. Pallidocin MIC values for thermophilic bacteria are extremely low when compared to the values for *B. megaterium* DSM319 and *B. cereus* ATCC14579. Notably, glycosylated leaderless Hyp1 core peptide demonstrated a different activity spectrum against thermophilic bacteria as compared to pallidocin. As in case of sublancin, the susceptible strains relatively easily develop resistance to pallidocin. The mechanism of resistance development will be the subject of our future study.

******Studies on sublancin and thurandacin have generally been focused on in vitro analysis and on the glycosyltransferase as potential tool for antibody generation and other purposes. *S*-linked glycopeptides have been highlighted to be more chemically and biologically stable compounds than *O*-linked glycopeptides^13,21^. Here, we identified and characterized antibacterial peptide pallidocin and expanded the currently small glycocin family. In addition, we demonstrated that glycosyltransferase PalS can be used as a tool for the biosynthesis of glycosylated antibacterial peptides in vivo. We have developed a system for heterologous expression and screening for hypothetical sublancin-type precursors with a minimal number of genes required. Pallidocin or Hyp1 could be applied in industrial processes facing thermophilic bacterial contaminations.

## Methods

### Mass spectrometry analysis

Matrix-assisted laser desorption/ionization time-of-flight mass spectrometry (MALDI-TOF-MS) analysis was carried out using a Voyager-DE-Pro (Applied Biosystems) at the Interfaculty Mass Spectrometry Center (IMSC) of the University Medical Center Groningen (UMCG). One microliter of analyte was spotted onto an MALDI target and dried under ambient conditions. One microliter of matrix (saturated α-cyano-4-hydroxy-cinnamic acid matrix in 50% ACN/50% water with 0.1% TFA) was spotted onto the dried sample on the MALDI target and dried under ambient conditions prior to analysis. The Voyager-De-Pro was set in linear positive mode. The spectrum range was 3000–10,000. Voltage settings were 25,000 V acceleration, 93% grid, 0.1% guide wire. A data Explorer 4.9 (Applied Biosystems) was used to process and analyze the acquired data.

High-resolution mass spectrometry (LC-ESI-Q-MS and MSMS) was carried out on a Q Exactive Hybrid Quadrupole-Orbitrap MS system (Thermo Scientific) combined with an UltiMate 3000 RSLC system (Thermo Scientific) at the IMSC of the UMCG. Each sample was injected into the UltiMate 3000 UHPLC system consisting of a quaternary pump, an autosampler and a column oven, which was coupled by a HESI-II electrospray source to the Q Exactive Orbitrap mass spectrometer (all Thermo Scientific). A Kinetex EVO-C18 (2.6 µm particles, 100 × 2.1 mm) column (Phenomenex) was used. The eluents for the LC separation were (A) water and (B) acetonitrile (ACN) both containing 0.1% formic acid. The following gradient was used: 5% B until 0.5 min, then linear gradient to 90% B in 4.5 min. This composition was held for 2 min, after which a switch back to 5% B was performed within 0.1 min. After 2.9 min of equilibration, the next injection was performed. The LC flow rate was 500 µL/min, the LC column was kept at 60 °C and the injection volume was 10 µL. The HESI-II electrospray source was operated with the parameters recommended by the MS software for the LC flow rate used (spray voltage 3.5 kV (positive mode)); other parameters were sheath gas 50 AU, auxiliary gas 10 AU, cone gas 2 AU, capillary temperature 275 °C, heater temperature 400 °C. The samples were measured in positive mode from m/z 500 to 2000 at a resolution of 70,000 @ m/z 200. The instrument was calibrated in positive mode using the Pierce LTQ Velos ESI Positive Ion Calibration Solution (Thermo Scientific). The system was controlled using the software packages Xcalibur 4.1, SII for Xcalibur 1.3 and Q-Exactive Tune 2.9 (all Thermo Scientific). The Xtract-algorithm within Xcalibur was used for deconvolution of the isotopically resolved data to a monoisotopic spectrum represented in Supplementary Figures 3-9, 16, 17, 21, 26 and 27. For spectra represented in Supplementary Figures 14, 15, 20, and 22-25, manual deconvolution was calculated by formula 1. Theoretical peptide masses were calculated using a web tool ProteinProspector (MS-Product/MS-Isotope) at http://prospector.ucsf.edu/prospector/mshome.htm.

1 $$\begin{array}{l}{\mathrm{m/z \times charge - }}\left( {{\mathrm{mass}}\,{\mathrm{of}}\,{\mathrm{the}}\,{\mathrm{attached}}\,{\mathrm{H}}^ + } \right) + \left( {{\mathrm{1 \times mass}}\,{\mathrm{of}}\,{\mathrm{H}}^ + } \right) \\ \quad = {\mathrm{peptide}}\,{\mathrm{molecular}}\,{\mathrm{weight}}\,\left[ {{\mathrm{M}} + {\mathrm{H}}} \right]^ + .\hfill \end{array}$$

### Genomic DNA analysis

The pallidocin producer strain was identified as *Aeribacillus pallidus* 8 (previously referred to as *Geobacillus sp*. 8 ^32^). A Genome-to-Genome Distance Calculator (GGDC)^36,49^ for digital DNA−DNA hybridization (dDDH) analysis at http://ggdc.dsmz.de/home.php was applied for the analysis of the genome with accession number LVHY00000000.1), which has been sequenced previously^37^.

Genomic DNA was submitted for bacteriocin mining to the BAGEL4 server^38^ at http://bagel4.molgenrug.nl/. An operon coding for a putative glycocin was identified (Fig. 1a) in the genome. The information on the biosynthetic gene cluster *pal*, responsible for in vivo production of pallidocin and containing the genes *palA*, *palS*, *palT*, *paldbA*, and *paldbB*, was accessed via the National Center for Biotechnology Information (NCBI) at http://www.ncbi.nlm.nih.gov/. The gene cluster is of chromosomal origin with the length of 4805 nucleotides and is found between base pairs 2768 and 7572 in the contig with accession number NZ_LVHY01000134.1.

Peptide and protein sequences were submitted to the NCBI database for BLASTp analysis at https://blast.ncbi.nlm.nih.gov/Blast.cgi. BLASTp analysis revealed that *palA* encodes for the 61 amino acid precursor peptide (Fig. 1b) with 39% sequence similarity to the sublancin 168 precursor SunA of *B*. *subtilis* (accession number NP_390031.1). *palS* encodes for a protein with 53% sequence similarity to the SunS-like family peptide glycosyltransferase of *B*. *pseudomycoides* (accession number WP_097849814.1). *palT* encodes for a protein with 38% sequence similarity to the SunT-like superfamily leader cleavage/ABC-type transporter of *Paeniclostridium sordellii* (accession number WP_057576215.1). *paldbA* encodes for a protein with 50% sequence similarity to the thioredoxin-like enzyme of *B*. *megaterium* (accession number WP_061859981.1). *paldbB* encodes a protein with 47% sequence similarity to the DsbB-like disulfide bond formation protein B of *B*. *cereus* (accession number WP_098593227.1).

BLASTp analysis revealed that Hyp1 (accession number WP_061859978.1) is encoded in the genome of *Bacillus megaterium* BHG1.1 (accession number LUCO00000000). The genome encodes the Hyp1 biosynthetic gene cluster, which is 5013 bp in length and encodes for proteins Hyp1S (accession number WP_081113339.1), Hyp1T (accession number WP_061859980.1), Trx (accession number WP_061859981.1), DsbB (accession number WP_061859982.1), and Hyp1U (accession number WP_061859983.1).

BLASTp analysis revealed that Hyp2 (accession number WP_035395705.1) is encoded in the genome of *Bacillus* sp. JCM 19047 (accession number BAWC00000000). The genome encodes the Hyp2 biosynthetic gene cluster, which is 3932 bp in length and encodes for proteins: Hyp2T (accession number WP_035395706.1), Trx (accession number WP_035395707.1), and Hyp2S (accession number GAF22634.1).

### Bacteriocin activity assays

A colony of indicator strain *P. genomospecies* 1 NUB36187 (BGSC 9A11) was spread on a Nutrient broth (NB) medium agar plate containing: 1% tryptone (BD Bacto), 0.5% beef extract (BD BBL), 0.5% NaCl (Merck), 1.5% agar (BD Bacto), and incubated overnight at 60 °C. Grown biomass was collected with sterile inoculum loop and spread on a fresh NB agar plate and incubated for 4 h at 60 °C. After the incubation (before cells starts to form spores), all biomass from the plate was washed with NB medium and the cell suspension was adjusted to OD (600 nm) of 1. The suspension was mixed with liquid NB agar medium (55 °C) at the ratio 1:100 and mixed thoroughly. Fifteen milliliters of the resulting cell suspension was dispersed in a Petri plate and left to solidify. For a well diffusion assay, wells were cut out in the solid medium in the Petri plate and filled with 50 µL of serial twofold dilutions of samples. The titer was defined as the reciprocal of the highest dilution that resulted in inhibition of the indicator strain.

For a spot on lawn assay, samples of 10 µL were spotted on the solid medium in the Petri dish and incubated at 60 °C overnight. *P. genomospecies* 1 NUB36187 (BGSC 9A11) was used as sensitive strain for all experiments. Antibacterial activities of hypothetical glycocins were tested against other thermophilic and mesophilic strains by the same approach. When the mesophilic strains were tested the incubations were performed at 37 °C. Analyzed bacteriocins were screened for their antibacterial activities against *B. cereus* ATCC14579, *B. megaterium* DSM319, *Geobacillus* sp. B4113, *G. stearothermophilus* B4109, B4111, B4112, B4114, B4161, B4163, *P. toebii* B4110, B4162, *P. caldoxylosilyticus* B4119 and *C. debilis* B4165.

### DNA amplification by PCR

A single PCR mix included Phusion HF Buffer (Thermo Scientific), 2.5 mM dNTPs mix (Thermo Scientific), 1.5 mM MgCl_2_ (Thermo Scientific), *PfuX7* DNA polymerase (homemade), primers (0.5 μM each), and 1 ng/µL DNA template. Target DNA was PCR-amplified by 30 cycles of denaturing (94 °C for 30 s), annealing (5 °C or more lower then *T*_m_ for 30 s), and extending (68 °C for 1 min per 1 kbp). Amplifications were confirmed by 1 or 2% agarose gel electrophoretic analyses. The list of primers and their sequences is provided in Supplementary Table 8.

### DNA cloning

DNA digestion was performed with restriction endonucleases purchased from Thermo Scientific and according to the manufacturer’s recommendations. Amplified or digested DNA was always cleaned with a NucleoSpin Gel and PCR Clean-up Extraction Kit (Macherey−Nagel), unless stated otherwise. A T4 DNA Ligase (Thermo Scientific) was used for DNA ligations, according to the manufacturer’s recommendations, unless stated otherwise. The ligation products were transformed to *E. coli* TOP10 cells by electroporation. Cells were plated on Lysogeny broth (LB) agar plates with appropriate antibiotics and grown at 37 °C overnight. Several colonies were picked and tested by colony PCR, to confirm whether the insert is present in the vector. Colonies with correct inserts were inoculated into LB medium with the appropriate antibiotic. The cultures were grown at 37 °C overnight, and plasmids were isolated using a NucleoSpin Plasmid Extraction Kit (Macherey−Nagel). DNA sequences of inserts in isolated plasmids were always confirmed by DNA sequencing. For gene expression, plasmid DNA was transformed to *E. coli* BL21(DE3) by electroporation. Cells were plated on LB agar plates with appropriate antibiotics and grown at 37 °C overnight. Several colonies were picked and inoculated into LB medium with the appropriate antibiotic, grown overnight at 37 °C, mixed with glycerol (glycerol end concentration 20%) and stored at −80 °C for further use.

### Cloning of pallidocin biosynthetic gene cluster *pal*

*A. pallidus* 8 was grown in a Bacto Brain Heart Infusion medium (BD Diagnostics) at 55 °C in a shaking incubator. Genomic DNAs were extracted with a GenElute Kit (Sigma-Aldrich) according to the manufacturer’s recommendations. The whole gene cluster *pal* encoding the pallidocin biosynthetic machinery (Fig. 1a) was amplified by PCR as a single unit (4805 bp) using F-PalA-USER and R-PalA-USER primers and *A. pallidus* 8 genomic DNA as template. The pBAD24 vector was PCR-amplified using F-pBAD-USER and R-pBAD-USER primers and pBAD24 plasmid as the template. Obtained PCR products were ligated by USER Enzyme (NEB), according to the manufacturer’s protocol, and transformed to *E. coli* TOP10 cells by electroporation. Colony PCR method using F-pBAD24 and R-pBAD24 primers was used for selection of positive transformants. Obtained construct pBAD24-pal was propagated in *E. coli* TOP10 cells and isolated. The presence of the cloned insert in the construct was confirmed by PCR (Supplementary Fig. 1). To amplify the insert the following pairs of primers were used together with pBAD24-pal as the template: the whole gene cluster *pal* (F-PalA-BspHI and R-BdbB); *palA* (F-PalA-BspHI and R-PalA-HindIII); *palS* (F-PalS-In-Fusion and R-PalS-In-Fusion); *palT* (F-PalT-In-Fusion and R-PalT-In-Fusion); *bdbA* (F-BdbA and R-BdbA); *bdbB* (F-BdbB and R-BdbB).

The sequence of the insert (*pal* gene cluster) was confirmed by DNA sequencing. DNA sequencing was performed with primers: F-pBAD24, R-pBAD24, Pal0, Pal1, Pal2, Pal3, Pal4, Pal5, and Pal6. The insert *pal*, starting with the start codon for the PalA, was cloned into the MCS behind the arabinose promoter and RBS of the pBAD24 vector. Sequences of primers are provided in Supplementary Table 8. For the protein expression pBAD24-pal was transformed to *E. coli* BL21(DE3).

### Overexpression of *pal* and purification of pallidocin

*E. coli* BL21(DE3) cells transformed with pBAD24-pal was grown overnight at 37 °C in LB medium containing ampicillin (50 µg/mL) and inoculated to 1 L of M9-ampicillin minimal medium (12.8 g/L Na_2_HPO_4_ × 7H_2_O, 3 g/L KH_2_PO_4_, 0.5 g/L NaCl, 1 g/L NH_4_Cl, 0.24 g/L MgSO_4_, 0.11 g/L CaCl_2_, and 4 mL glycerol in MiliQ water) at the ratio 1:100. The cells were grown at 37 °C to the OD (600 nm) of 0.6–0.7, then arabinose was added to the final concentration of 2 mM and the culture was incubated at 37 °C for additional 16 h. Cells were harvested by centrifugation at 10,000 × *g* for 15 min at 4 °C. Supernatant was collected, immediately filtered through a 0.45 µm filter and uploaded on an Econo-Column chromatography column, 2.5 × 30 cm (Bio-Rad) filled with a 50 g of Amberlite XAD16N hydrophobic polyaromatic resin (Sigma-Aldrich), which was previously equilibrated with deionized water. After sample loading, the column was washed with 500 mL deionized water. Elution was performed with 250 mL of 100% methanol. The eluate was collected, diluted with deionized water (ratio 1:3) and lyophilized in a freeze-dryer. Pellets were dissolved in 100 mL of 50 mM lactic acid buffer (pH 4.5) and filtered through a 0.45 µm filter. Then, it was loaded on an NGC system (Bio-Rad) equipped with a HiTrap SP HP 5 mL cation exchange column (GE Healthcare Life Sciences), which was previously equilibrated with 50 mM lactic acid buffer (pH 4.5). The column was then washed with 50 mM lactic acid buffer (pH 4.5) and elution performed with 50 mM lactic acid buffer containing 300 mM NaCl (pH 4.5). The eluate was mixed with trifluoroacetic acid (TFA) to the end concentration of 0.1% and loaded on an RP-HPLC system (Agilent) equipped with a Jupiter Proteo, C-12, 250 × 10 mm column (Phenomenex). The column was equilibrated in 5% of solvent B (solvent A = MiliQ water with 0.1% TFA, solvent B = ACN with 0.1% TFA). Bacteriocin was eluted by an increase of solvent B up to 60% over 80 min with a flow rate of 2 mL/min. Elution fractions were tested for antibacterial activity against *P. genomospecies* 1 NUB36187 using a drop on a lawn assay. Active fractions were lyophilized, pellets dissolved in a solution containing 6 M guanidine-HCl and 0.1% TFA, and applied on a Jupiter Proteo, C-12, 250 × 4.6 mm column (Phenomenex) which was equilibrated in 5% of solvent B. Bacteriocin was eluted by an increase of solvent B up to 60% over 80 min with a flow rate of 1 mL/min. Elution fractions were tested for antibacterial activity against *P. genomospecies* 1 NUB36187 using a spot on lawn assay. Samples of elution fractions with antibacterial activity were analyzed by MALDI-TOF-MS, and the rest were lyophilized and stored at −80 °C until further use.

### Cloning for gene coexpression

The gene *palA* was amplified by PCR using F-PalA-BspHI and R-PalA-HindIII primers, the gene *palS* was amplified by PCR using F-PalS-In-Fusion and R-PalS-In-Fusion primers. The gene *palT* was amplified by PCR using F-PalT-In-Fusion and R-PalT-In-Fusion primers. The DNA region containing two genes *palS* and *palT* was amplified by PCR using F-PalS-In-Fusion and R-PalT-In-Fusion primers. *A. pallidus* 8 genomic DNA was used as the template to amplify the genes.

Amplified *palA* was double digested with BspHI and HindIII. pRSFDuet-1 vector was double digested with NcoI and HindIII. Both digestion products were cleaned and ligated by a conventional cloning. The ligation mixtures were transformed to *E. coli* TOP10 cells by electroporation. Positive colonies were selected by colony PCR using F-pRSFDuet-1 and R-pRSFDuet-1 primers. Positive clones were propagated for the plasmid isolation. The sequences of the inserts in the constructs were confirmed by DNA sequencing using F-pRSFDuet-1 and R-pRSFDuet-1 primers.

A site-directed mutagenesis approach was used to introduce the His7-tag sequence (GGHHHHHHH) in the C-terminus of the PalA peptide. A new construct pRSFDuet-1 encoding *palA-his* was generated by PCR amplification using 5′-phosphorylated primers F-PalA-His and R-PalA-His. Construct pRSFDuet-1-*palA* was used as template. The PCR product was cleaned, ligated and transformed to *E. coli* TOP10. Positive clones were confirmed by colony PCR and propagated for plasmid isolation. The His7-tag encoding sequence in the resulting construct (pRSFDuet-1-*palA-his*) was confirmed by DNA sequencing using F-pRSFDuet-1 and R-pRSFDuet-1 primers.

To introduce a Factor Xa cleavage site and a His-tag (N-terminus) in the PalA peptide, a *his-Xa-palA* gene was engineered. First, the gene *palA* was amplified by PCR using F-PalA-BamHI and R-PalA-HindIII^2^ primers and *A. pallidus* 8 genomic DNA as the template. PCR product *palA* and vector pRSFDuet-1 were double digested with BamHI and HindIII according to the manufacturer’s recommendations. Resulting products were cleaned, ligated by conventional cloning and transformed to *E. coli* TOP10 by electroporation. Positive clones were selected by colony PCR using F-pRSFDuet-1 and R-pRSFDuet-1 primers and propagated for isolation of plasmids. The sequence of the insert was confirmed by DNA sequencing using F-pRSFDuet-1 and R-pRSFDuet-1 primers. The insert, *palA* gene, in the resulting construct pRSFDuet-1-his-palA was introduced behind the His6-tag encoding sequence (MGSSHHHHHHSQDP).

Next, a site-directed mutagenesis approach was used to incorporate a Factor Xa proteolytic cleavage site to the N-terminal part of the PalA peptide (Supplementary Fig. 13). Four wild-type peptide residues LQGS were changed to IEGR. pRSFDuet-1 coding for *his-Xa-palA* was amplified by PCR using 5′-phosphorylated primers F-PalA-Xa and R-PalA-Xa and template pRSFDuet-1-his-palA, then ligated and transformed to *E. coli* TOP10 by electroporation. Positive clones were selected by colony PCR followed by plasmid isolations. Correct sequence of constructed pRSFDuet-1-his-Xa-palA vector was confirmed by DNA sequencing using F-pRSFDuet-1 and R-pRSFDuet-1 primers. Sequences of the primers are provided in Supplementary Table 8.

Synthetic genes of sublancin 168 (*sunA-his*), glycocin F (*gccF-his*), enterocin F4-9 (*enfA4-9-his*), hypothetical peptide 1 (*hyp1-his*), and hypothetical peptide 2 (*hyp2-his*) were synthesized by GenScript with codon optimization for *E. coli* and delivered in a pUC57 vector. The genes encode glycocin precursors with leaders and His6-tag sequences (HHHHHH) in the C-terminuses of the peptides. All synthesized genes were cloned into the pRSFDuet-1 vector and transformed to *E. coli* TOP10. The presences of the inserts in the vector pRSFDuet-1 were confirmed by PCR (Supplementary Fig. 19). To amplify the inserts, the following primer pairs were used: *sunA-his* (primers F-SunA-BamHI and R-SunA-HindIII); *hyp1-his* (primers F-Hyp1 and R-Hyp1); *hyp2-his* (primers F-Hyp2 and R-Hyp2); *gccF-his* (primers F-GccF and R-GccF); *enfA4-9-his* (primers F-EnfA4-9 and R-EnfA4-9). Constructed pRSFDuet-1 vectors coding for the cloned genes were used as the templates, respectively. Sequences of the inserts in the plasmids were also confirmed by DNA sequencing using F-pRSFDuet-1 and R-pRSFDuet-1 primers. Sequences of the primers are provided in Supplementary Table 8.

A site-directed mutagenesis approach was used to engineer *core_hyp1-his* and *core_hyp2-his* genes coding for Hyp1-His and Hyp2-His core peptides without leader sequences and with a His6-tag (HHHHHH) in the C-terminuses (Supplementary Fig. 13). pRSFDuet-1-core_hyp1-his was amplified by PCR using 5′-phosphorylated primers F-Hyp1-Leaderless and R-Leaderless. pRSFDuet-1-core_hyp2-his was amplified by 5′-phosphorylated primers F-Hyp2-Leaderless and R-Leaderless. pRSFDuet-1-hyp1-his and pRSFDuet-1-hyp2-his vectors were used as templates, respectively. Obtained PCR products were ligated and transformed to *E. coli* TOP10 by electroporation. Positive clones were selected by colony PCR using F-pRSFDuet-1 and R-pRSFDuet-1 primers and propagated for isolation of plasmids. The presences of the genes coding for leaderless peptides in the vectors were confirmed by PCR (Supplementary Fig. 19). We amplified the insert *core_hyp1-his* by primers F-Hyp1-Leaderless and R-Hyp1-HindIII, and pRSFDuet-1-core_hyp1-his vector as template. The insert *core*_*hyp2-his* was amplified by primers F-Hyp2-Leaderless and R-Hyp2, and pRSFDuet-1-core_hyp2-his vector as template. In addition, the inserts encoding Hyp1-His and Hyp2-His core peptides were confirmed by DNA sequencing using F-pRSFDuet-1 and R-pRSFDuet-1 primers. Sequences of the primers are provided in Supplementary Table 8.

To generate pBAD24 vectors coding for genes *palS*, *palT* and *palST*, the pBAD24 vector was double digested with NcoI and PstI, separated by DNA electrophoresis and extracted from agarose gel using a NucleoSpin Gel and PCR Clean-up Extraction Kit (Macherey−Nagel). Next, PCR-amplified *palS*, *palT* and *palST* were ligated with the double digested pBAD24 vector using a Quick-Fusion Cloning Kit (Bimake) according to the manufacturer’s recommendations. After the ligation, the mixtures were diluted with MiliQ in ratio 1:5 and transformed to *E. coli* TOP10 by electroporation. Positive clones were selected by colony PCR using F-pBAD24 and R-pBAD24 primers and propagated for isolation of plasmids. The presences of the cloned inserts in the constructs were confirmed by PCR (Supplementary Fig. 12). We amplified the insert *palA-his* by primers F-PalA-BspHI and R-PalA-HindIII, and pRSFDuet-1-palA-his vector as template. The insert *palS* was amplified by primers F-PalS-In-Fusion and R-PalS-In-Fusion, and pBAD24-palS vector as template. The insert *palT* was amplified by primers F-PalT-In-Fusion and R-PalT-In-Fusion, and pBAD24-palT vector as template. The insert *palST* was amplified by primers F-PalS-In-Fusion and R-PalT-In-Fusion, and pBAD24-palST vector as template.

Sequences of the inserts in the resulting constructs (pBAD24-*palS*, pBAD24-*palT* and pBAD24-*palST*) were also confirmed by DNA sequencing. We used F-pBAD24, R-pBAD24, and Pal1 primers for pBAD24-*palS* sequencing; F-pBAD24, R-pBAD24, Pal3, Pal4, and Pal5 primers for pBAD24-*palT* sequencing; F-pBAD24, R-pBAD24, Pal1, Pal2, Pal3, Pal4, and Pal5 primers for pBAD24-*palST* sequencing.

Genes of precursors were cloned in MCS1 behind the IPTG promoter and RBS of the pRSFDuet-1 vector. Genes *palS/palT/palST* were cloned into the MCS behind the arabinose promoter and RBS of the pBAD24 vector. Sequences of primers are provided in Supplementary Table 8. The vectors pRSFDuet-1 and pBAD24 coding for *palS/palT/palST* were transformed to *E. coli* BL21(DE3) for protein expression experiments.

### Coexpression of glycocin precursors with PalS and PalT

*E. coli* BL21(DE3)-containing vectors pRSFDuet-1-*palA-his* and pBAD24 coding for either *palS, palT* or *palST* were grown at 37 °C overnight in LB medium containing ampicillin (50 µg/mL) and kanamycin (30 µg/mL). The next day it was inoculated into 100 mL of fresh LB−ampicillin−kanamycin medium in the ratio 1:100 and grown at 37 °C to the OD (600 nm) of 0.6−0.7. Arabinose and IPTG were added to a final concentration of 1 mM of each and the culture was grown for additional 4 h at 37 °C. After the induction, cells were harvested by centrifugation at 7000 × *g* for 15 min at 4 °C and resuspended in 5 mL of binding buffer (20 mM NaH_2_PO_4_, 500 mM NaCl, pH 7.4).

pRSFDuet-1 vectors with cloned glycocin precursor genes: *sunA-his/gccF-his/enfA4-9-his/hyp1-his/hyp2-his*, were coexpressed in *E. coli* BL21(DE3) together with the pBAD24 vector coding for *palS/palT/palST*, respectively. The pRSFDuet-1 vector coding for *his-Xa-palA* was coexpressed in *E. coli* BL21(DE3) together with the pBAD24 vector coding for *palS*. The coexpression was performed in the same approach as the coexpression of pRSFDuet-1-palA-his with pBAD24-pasS. pRSFDuet-1 vector encoding *core_hyp1-his/core_hyp2-his* was coexpressed with pBAD24 vector encoding *palS* in *E. coli* BL21(DE3). The coexpression was induced for 7 h by the same approach as was coexpression of pRSFDuet-1-palA-his with pBAD24-pasS.

### Purification of the peptides

The obtained cell suspension after coexpression was sonicated on ice for 20 min using a VCX 130 Sonicator with cycle 10 s ON and 10 s OFF. The highest concentration of the produced peptide was found in the insoluble fraction of lysed cells. Cell debris was removed by centrifugation at 15,000 × *g* for 20 min at 4 °C. The supernatant was discarded and the pellet of insoluble fraction was resuspended and sonicated at the same conditions in 5 mL of binding buffer containing 6 M guanidine-HCl. The sample was filtered through a 0.45 μm filter and applied to an NGC system (Bio-Rad) equipped with a HisTrap FF 1 mL (GE Healthcare Life Sciences) immobilized metal affinity chromatography (IMAC) column pre-equilibrated with binding buffer containing 4 M guanidine-HCl. After sample application, the column was washed with binding buffer containing 4 M guanidine-HCl. The peptide was eluted with elution buffer (20 mM NaH_2_PO_4_, 500 mM NaCl, 500 mM imidazole, pH 7.4) containing 4 M guanidine-HCl.

The fractions containing eluted peptides were further purified by an RP-HPLC system (Agilent) equipped with Jupiter Proteo, C-12, 250 × 10 mm column (Phenomenex). The eluate was mixed with TFA to reduce the pH to 2–3 and loaded on the column equilibrated in 5% of solvent B. The peptides were eluted by an increase of solvent B up to 60% over 60 min with a flow rate of 2 mL/min. All fractions were tested for antibacterial activity against *P. genomospecies* 1 NUB36187 using a drop on lawn assay. Additionally, elution fractions were analyzed by MALDI-TOF-MS and LC-ESI-MS.

### Leader cleavage of the pre-His-Xa-PalA-Glc peptide

Lyophilized pellets of purified glycosylated pallidocin precursor pre-His-Xa-PalA-Glc were dissolved in 1 mL of 6 M guanidine-HCl and loaded on gel filtration column PD-10 (GE Healthcare Life Sciences). The buffer exchange of the sample was performed according to the manufacturer’s recommendations. The column was pre-equilibrated with 50 mM tris-HCl, 100 mM NaCl, pH 7.5 buffer. The eluate was collected and mixed with CaCl_2_ to the end concentration of 2 mM. 10–20 µL of Factor Xa peptidase (enzyme concentration 1 mg/mL, NEB) was added to 0.5 mL of previously gel filtrated peptide solution (peptide concentration 0.5 mg/mL). The mixture was stored at room temperature for 3–6 h. After sample treatment with the peptidase, 4 mL of 6 M guanidine-HC and TFA, to quench the pH to 2–3, were added. Then, the sample was loaded on an RP-HPLC system (Agilent) equipped with a Jupiter Proteo, C-12, 250 × 4.6 mm column (Phenomenex) equilibrated in 5% of solvent B. The peptide was eluted by an increase of solvent B from 20% up to 60% over 80 min with a flow rate of 1 mL/min. Elution fractions were tested for antibacterial activity against *P. genomospecies* 1 NUB36187 using a drop on lawn assay. The elution fractions were analyzed by MALDI-TOF-MS. Elution fractions containing active and glycosylated pallidocin core peptide PalA-Glc were lyophilized by freeze-dryer. Pellets were stored at −20 °C or dissolved in MiliQ containing 50% ACN and 0.1% TFA solution for further use. The quantity of purified peptide was measured by a NanoPhotometer N60 (Implen). The molar absorptivity (extinction coefficient) was calculated (at 280 nm and 13,200/M/cm or at 205 nm and 171,330/M/cm) based on the peptide sequence. Calculations were performed by a web tool, provided at http://spin.niddk.nih.gov/clore^48^. Determined peptide quantities at 280 and 205 nm were the same. The yield of synthesized pallidocin from 200 mL of bacterial culture was ~30 µg.

### Iodoacetamide assays for detection of free cysteines

To detect the presence of free cysteine thiols in peptides, an iodoacetamide (IAA) assay was used. For the detection of free Cys residues in the native peptide, reactions contained 100 mM tris-HCl (pH 8.3), 40 mM IAA and the peptide. For the detection of free Cys residues in a reduced peptide, reactions contained 100 mM tris-HCl (pH 8.3), 10 mM TCEP, 40 mM IAA and the peptide. All reactions were in total volume of 1 mL, incubation conditions were 2 h at room temperature in the dark. The reaction mixtures were quenched with TFA to pH < 4. Samples were loaded on an RP-HPLC system (Agilent) equipped with a Jupiter Proteo, C-12, 250 × 4.6 mm column (Phenomenex) equilibrated in 5% of solvent B. Peptides were eluted by an increase of solvent B from 20% up to 60% over 40 min with a flow rate of 1 mL/min. Elution fractions containing peptides were tested for antibacterial activity against *P. genomospecies* 1 NUB36187 using a drop on lawn assay and further analyzed by MALDI-TOF-MS. The detection of free cysteines was determined by the presence or absence of thiol modifications, carboxyamidomethyl (CAM).

### Effects of pH on bacteriocin stability

50 mM buffer solutions of KCl-HCl pH 2, citric acid-sodium citrate pH 4, phosphate pH 6, tris-HCl pH 8 and sodium carbonate-sodium bicarbonate pH 10 were prepared for the following assay. Pallidocin was dissolved in MiliQ water containing 50% ACN and 0.1% TFA to the end concentration of 1 ng/µL. 13.5 µL of each buffer solution was mixed with 1.5 µL of pallidocin solution and the resulting mixtures were stored for 3 h at room temperature. After the incubation, 15 µL of 500 mM tris-HCl pH 7.5 buffer solution and 120 µL of NB medium were added to the mixtures. Next, serial twofold dilutions with NB medium were made for each mixture. Bacteriocin activity was tested for each sample with agar well diffusion assay.

### Effect of temperature on bacteriocin stability

Pallidocin was dissolved in MiliQ water containing 50% ACN and 0.1% TFA to the end concentration of 1 ng/µL. One hundred and fifty microliters of NB medium was mixed with 1.5 µL pallidocin solution and stored at room temperature for 24 h, 10 days and 30 days. In addition, samples were stored at different temperatures: 60 °C, 90 °C for 3 h and autoclaved for 15 min at 121 °C. After the incubation, serial twofold dilutions in NB medium were made for each mixture. Bacteriocin activity was tested for each sample with agar well diffusion assay.

### Determination of minimum inhibitory concentrations

MICs were determined as described by Wiegand et al.^50^ with some modifications. One colony of a sensitive strain was picked from an NB agar plate, inoculated to liquid NB medium and grown at 55 °C, in a shaking incubator until OD (600 nm) of 0.1 was reached. Then, the culture was diluted with NB medium till concentration of 10×10^5^ CFU/mL. One hundred and fifty microliters of fresh NB medium was mixed with 5 µL of pallidocin solution (1 ng/µL in 50% ACN and 0.1% TFA) and serial twofold dilutions with NB medium were made. Seventy-five microliters of resulting diluted pallidocin mixtures were transferred to a 96-well plate and mixed with 75 µL previously prepared cell suspension of sensitive strain. The final volume of the mixture in the well was 150 µL and the end concentration of the sensitive strain was 5×10^5^ CFU/mL. Positive controls, 150 µL mixture of NB medium with the sensitive strain (5×10^5^ CFU/mL), and negative controls, 150 µL mixture of NB medium with 5 µL of pallidocin solution (1 ng/µL in 50% ACN and 0.1% TFA), were prepared and dispersed in the same 96-well plates. The plate with a lid was placed in a plastic box (12 cm × 20 cm × 6 cm) with a wet paper towel to keep high humidity and prevent medium evaporation at high temperature. The plate was incubated for 18 h at 55 °C in a shaking incubator. After incubation the growth of bacteria was evaluated visually and by a plate reader. The analyses were performed in triplicate.

It should be noted that because of the high mutation rate and emergence of resistant mutants in some wells in the plate, the calculation of average MIC from three replicates are prone to variation. The final MIC value was determined by the lowest amount of pallidocin required to inhibit cell growth in a well. Because bacteria were grown at 55 °C, it was not possible to use a plate reader at this condition, as the instrument is not suited for measurements at high temperatures.

### Determination of the sugar modification of pallidocin

The presence of a sugar moiety on Cys25 of pallidocin core peptide was confirmed via acid-catalyzed methanolysis and derivatization of the sugar, analysis by gas chromatography-mass spectrometry (GC-MS), and comparison to derivatized sugar standards. Purified pallidocin was lyophilized in a glass tube, internal standard-mannitol and 0.5 mL of methanolic 1 M HCl (Sigma-Aldrich) were added. The mixture was heated at 85 °C overnight. The reaction was cooled at room temperature and neutralized by adding solid Silver Carbonate (Sigma-Aldrich) till pH 7. For re-N-acetylation, two drops of Acetic Anhydride (Sigma-Aldrich) was added, mixed and stored overnight at room temperature in the dark. Next day, the reaction mixture was centrifuged at 1000 × *g* for 2 min. Supernatant was transferred to a new glass tube. 0.5 mL of methanol was added to the silver salt pellets, mixed and centrifuged again. Supernatant was collected and pooled with the first one. The procedure was repeated twice. Collected supernatant was evaporated in a vacuum evaporator. Tri-methylation of the sugar was performed by adding 0.3 mL of silylation reagents (pyridine:hexamethyldisilazane:trimethyl-chlorosilane = 5:1:1). The mixture was incubated at room temperature for 30 min.

*Sugar standards mix*: d-mannose, d-galactose, d-glucose, *N*-acetylgalactosamine and *N*-acetylglucosamine were also treated and derivatized using the conditions described above.

The derivatized sugar of pallidocin and sugar standards mix were analyzed individually by a GCMS-QP2010 Plus system (Shimadzu) equipped with a Zebron ZB-1HT column, L = 30 m × I.D. = 0.25 mm × df = 0.25 μm (Phenomenex). The temperature gradient was from 140 to 240 °C at 4 °C per min. The carrier gas was helium and the flow rate set at 2.0 mL/min.

### Circular dichroism spectroscopy

Circular dichroism (CD) spectra were recorded using a J-815 CD Spectrometer (JASCO) with a cuvette of 0.1 cm path length in the range from 190 to 260 nm at 0.5 nm intervals. For CD spectrum measurement lyophilized pallidocin was dissolved in a solvent (40% ACN, 0.1% TFA) to a final concentration of 9 µM. The spectrum of scan was obtained with a 2 nm optical bandwidth. The baseline scans were collected with the solvent alone and then subtracted from the sample scan. The estimate of secondary structure content was made using spectra from 193 nm to 260 nm by the method of Raussens et al.^42^ at http://perry.freeshell.org/raussens.html.

### Code availability

The BAGEL4^38^ server (http://bagel4.molgenrug.nl/) was used for bacteriocin mining in the genome sequences. The strain was identified by submitting its genomic DNA sequence to a Genome-to-Genome Digital Calculator (GGDC)^36,49^ for digital DNA−DNA hybridization (dDDH) analysis at http://ggdc.dsmz.de/home.php. BLAST analyses of peptide and protein sequences were performed using the NCBI database at https://blast.ncbi.nlm.nih.gov/Blast.cgi.

Calculations of extinction coefficients for peptide’s absorbance at 280 and 205 nm were performed by a web tool Protein Calculator^51^ at http://spin.niddk.nih.gov/clore. Theoretical peptide mass calculations were performed using a web tool ProteinProspector (MS-Product/MS-Isotope) at http://prospector.ucsf.edu/prospector/mshome.htm. The estimate of secondary structure content based on far-UV CD spectroscopy was made by the method of Raussens et al.^42^ at http://perry.freeshell.org/raussens.html. The estimate and modeling of secondary structures based on amino acid sequences were made by PSIPRED^22^ web tool at http://bioinf.cs.ucl.ac.uk/psipred/.

### Reporting summary

Further information on experimental design is available in the Nature Research Reporting Summary linked to this article.

## Supplementary information

Supplementary Information

Peer Review File

Reporting Summary

Source Data

## Data Availability

Data supporting the findings of this work are available within the paper and its Supplementary Information files. A reporting summary for this article is available as a Supplementary Information file. The source data underlying Supplementary Figures 1, 11, 12, 19 and Supplementary Tables 5−7 are provided as a Source Data file. All other data are available from the corresponding author on request.
