# Supplementary material for: Heterologous biosynthesis and characterization of a glycocin from a thermophilic bacterium
Source: Nat Commun. 2019 Mar 7;10:1115. doi: 10.1038/s41467-019-09065-5 (PMC6405829; doi:10.1038/s41467-019-09065-5)
Supplement: Supplementary file 1 — Supplementary Information [file 41467_2019_9065_MOESM1_ESM.pdf]

Heterologous biosynthesis and characterization of a glycocin from a thermophilic bacterium

Kaunietis *et al.*

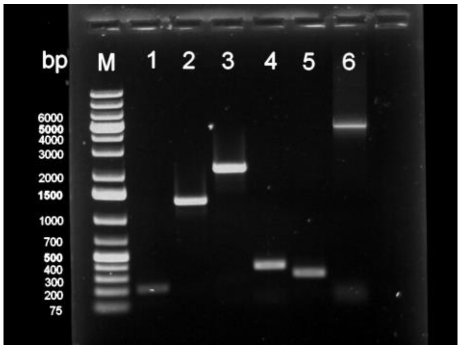

Supplementary Figure 1. DNA electrophoresis of PCR products amplified from the construct pBAD24-pal. Bp - base pairs, M - GeneRuler 1 kb Plus DNA Ladder (Thermo Fisher), 1 - *palA*, 2 - *palS*, 3 - *palT*, 4 - *paldbA*, 5 - *paldbB*, 6 - *pal*. Source data are provided as a Source Data file.

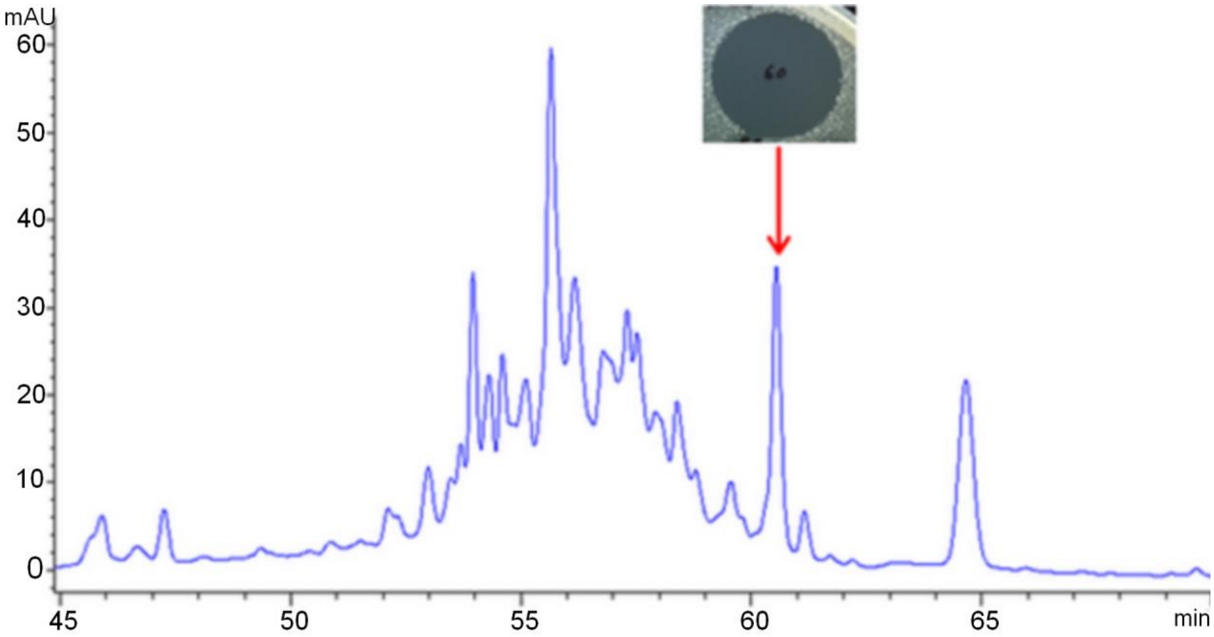

Supplementary Figure 2. Purification of pallidocin after expression of the whole biosynthetic gene cluster *pal*. Chromatogram represents the last step of purification by RP-HPLC. Inhibitory activity of elution fractions tested by spot on lawn assay. *P. genomospecies* 1 NUB36187 was used as a sensitive strain.

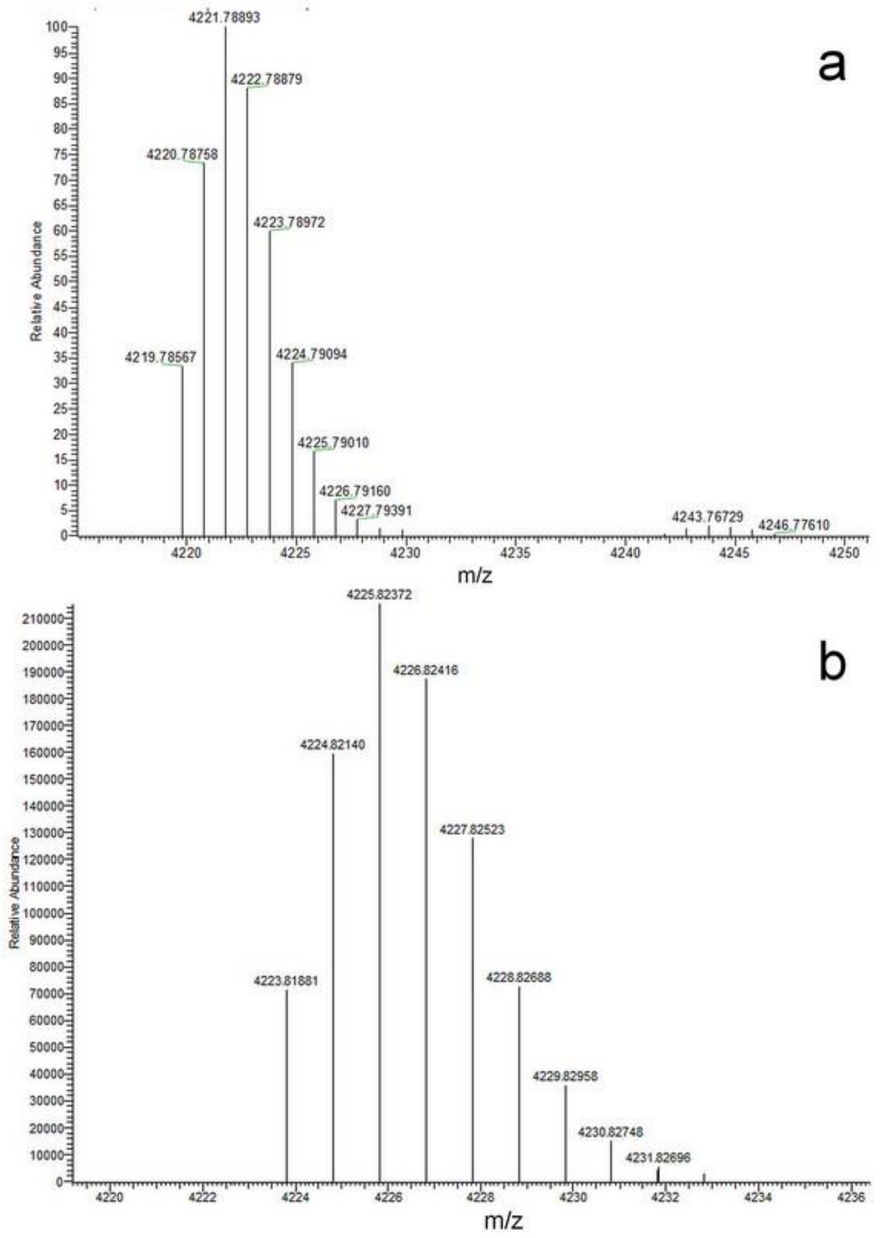

Supplementary Figure 3. LC-ESI-MS analysis of pallidocin derived after whole biosynthetic gene cluster *pal* expression in *E. coli*. The predicted monoisotopic mass $[M+H]^+$ of the unmodified pallidocin core peptide is 4061.76. (a) native pallidocin analysis, observed monoisotopic mass $[M+H]^+$ is 4219.79. (b) reduced with TCEP pallidocin analysis, observed monoisotopic mass $[M+H]^+$ is 4223.82. The results indicate that pallidocin has two disulfide bonds (-4) and a posttranslational modification (+162.05).

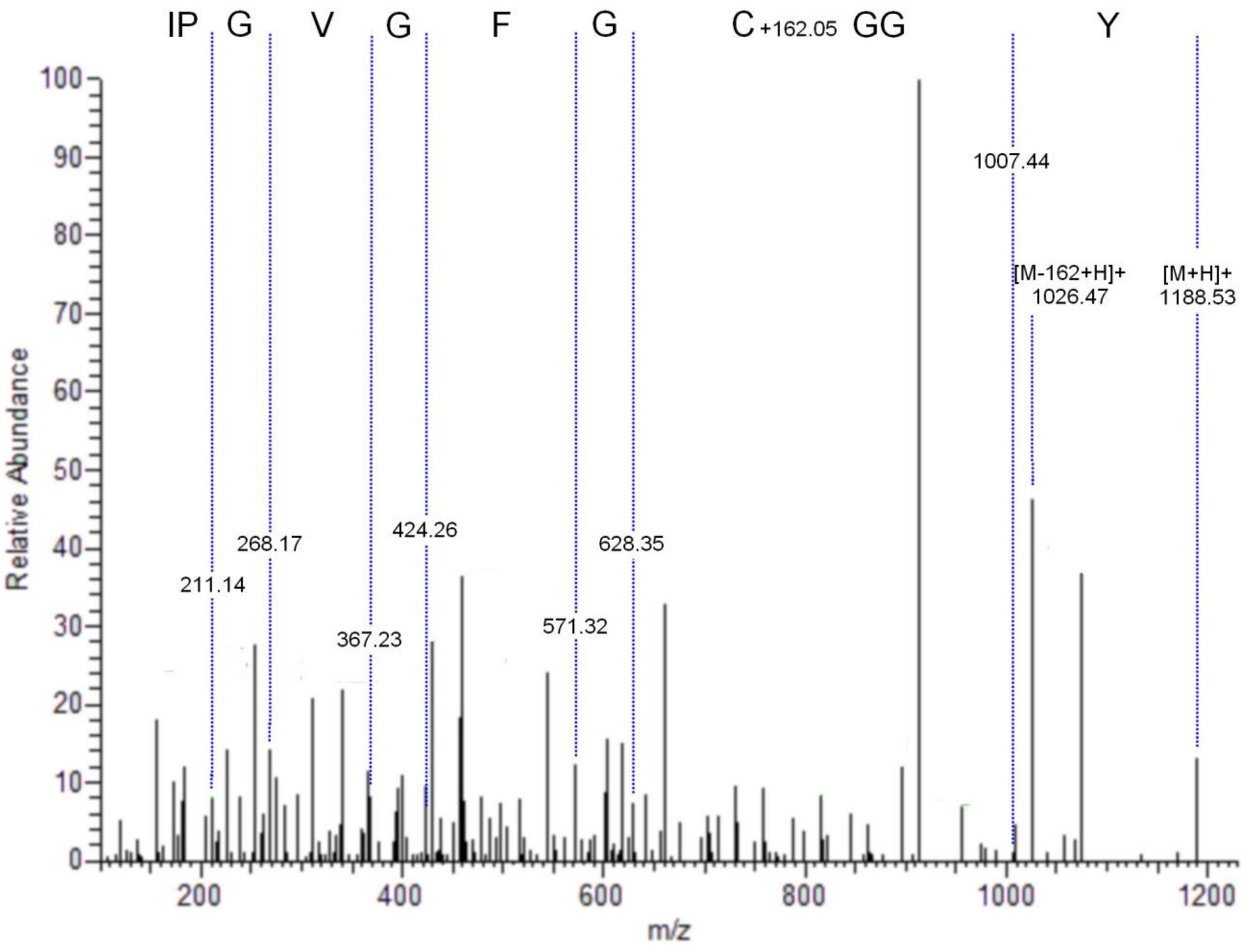

Supplementary Figure 4. ESI-Q-MSMS analysis of fragment IPGVGFGCGGY with indicated b ions after trial 1. The fragment was derived after pallidocin digestion with chymotrypsin. Theoretical monoisotopic mass $[M+H]^+$ of the sequence IPGVGFGCGGY is 1026.47, observed mass of the fragment is 1188.53. Fragment peaks of the peptide (1188.53) matching theoretical b ion masses of the IPGVGFGCGGY are indicated in the figure. The results confirm that Cys25 has a +162.05 posttranslational modification.

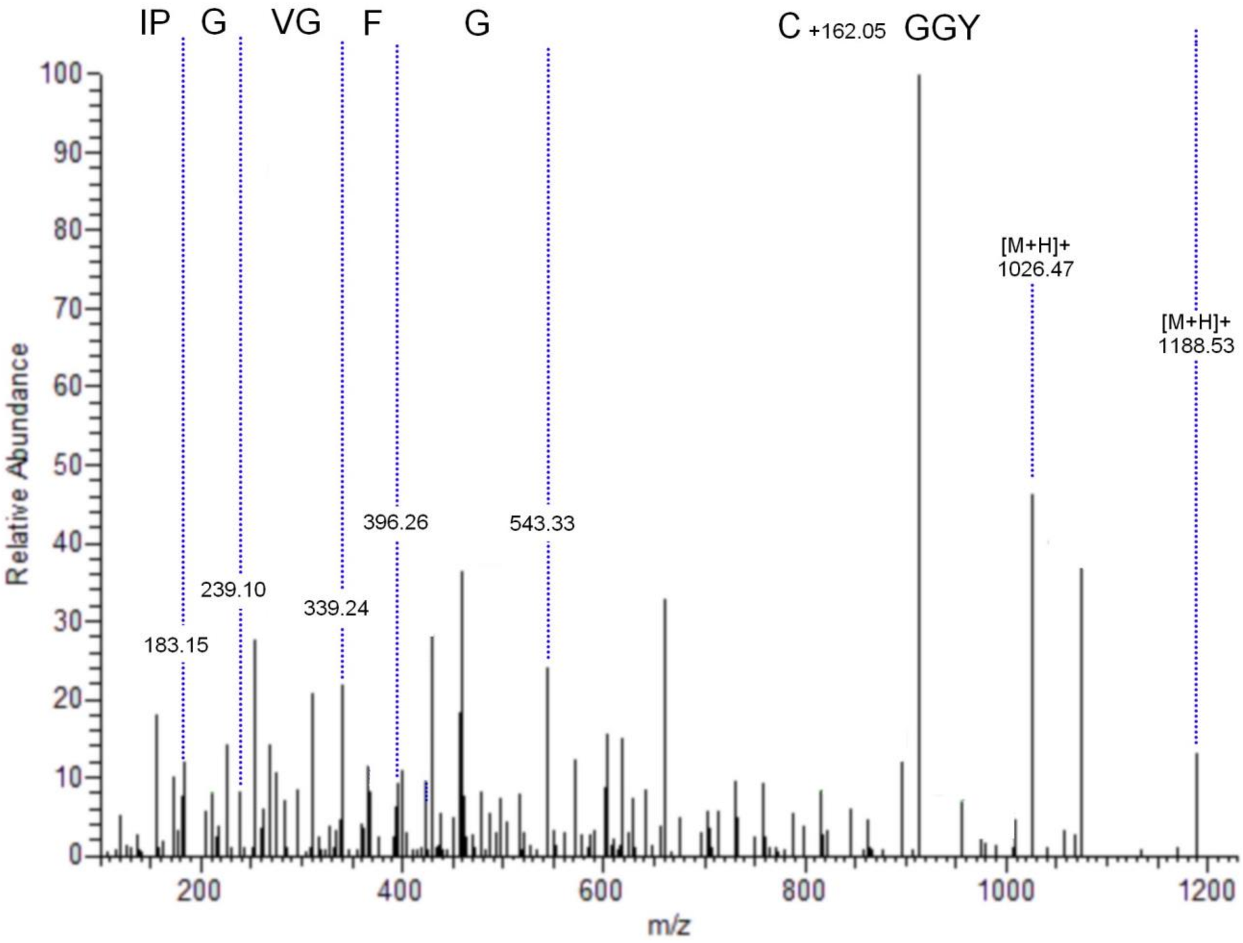

Supplementary Figure 5. ESI-Q-MSMS analysis of fragment IPGVGFGCGGY with indicated a ions after trial 1. The fragment was derived after pallidocin digestion with chymotrypsin. Theoretical monoisotopic mass $[M+H]^+$ of the sequence IPGVGFGCGGY is 1026.47, observed mass of the fragment is 1188.53. Fragment peaks of the peptide (1188.53) matching theoretical a ion masses of the IPGVGFGCGGY are indicated in the figure. The results confirm that Cys25 has a +162.05 posttranslational modification.

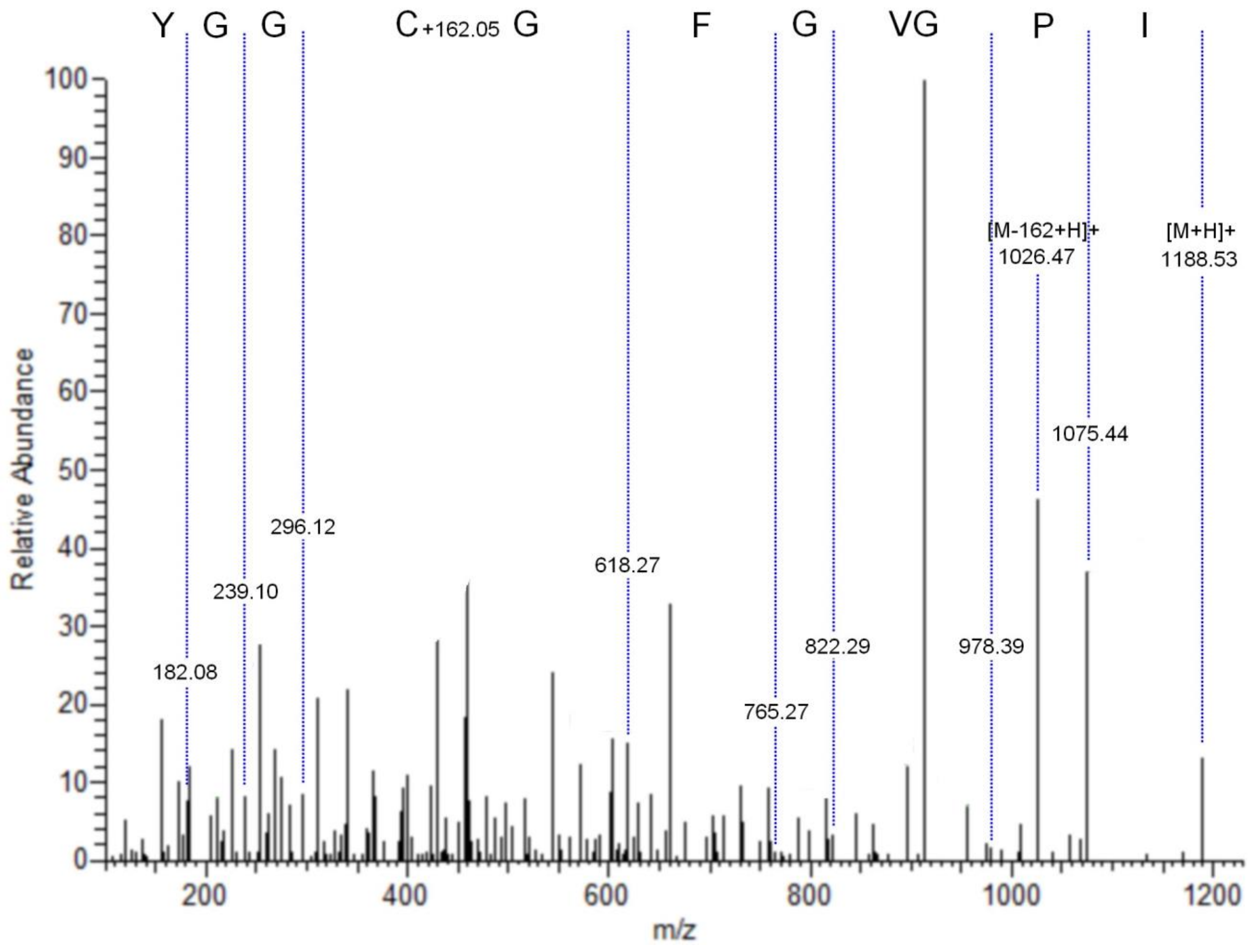

Supplementary Figure 6. ESI-Q-MSMS analysis of fragment IPGVGFGCGGY with indicated y ions after trial 1. The fragment was derived after pallidocin digestion with chymotrypsin. Theoretical monoisotopic mass $[M+H]^+$ of the sequence IPGVGFGCGGY is 1026.47, observed mass of the fragment is 1188.53. Fragment peaks of the peptide (1188.53) matching theoretical y ion masses of the IPGVGFGCGGY are indicated in the figure. The results confirm that Cys25 has a +162.05 posttranslational modification.

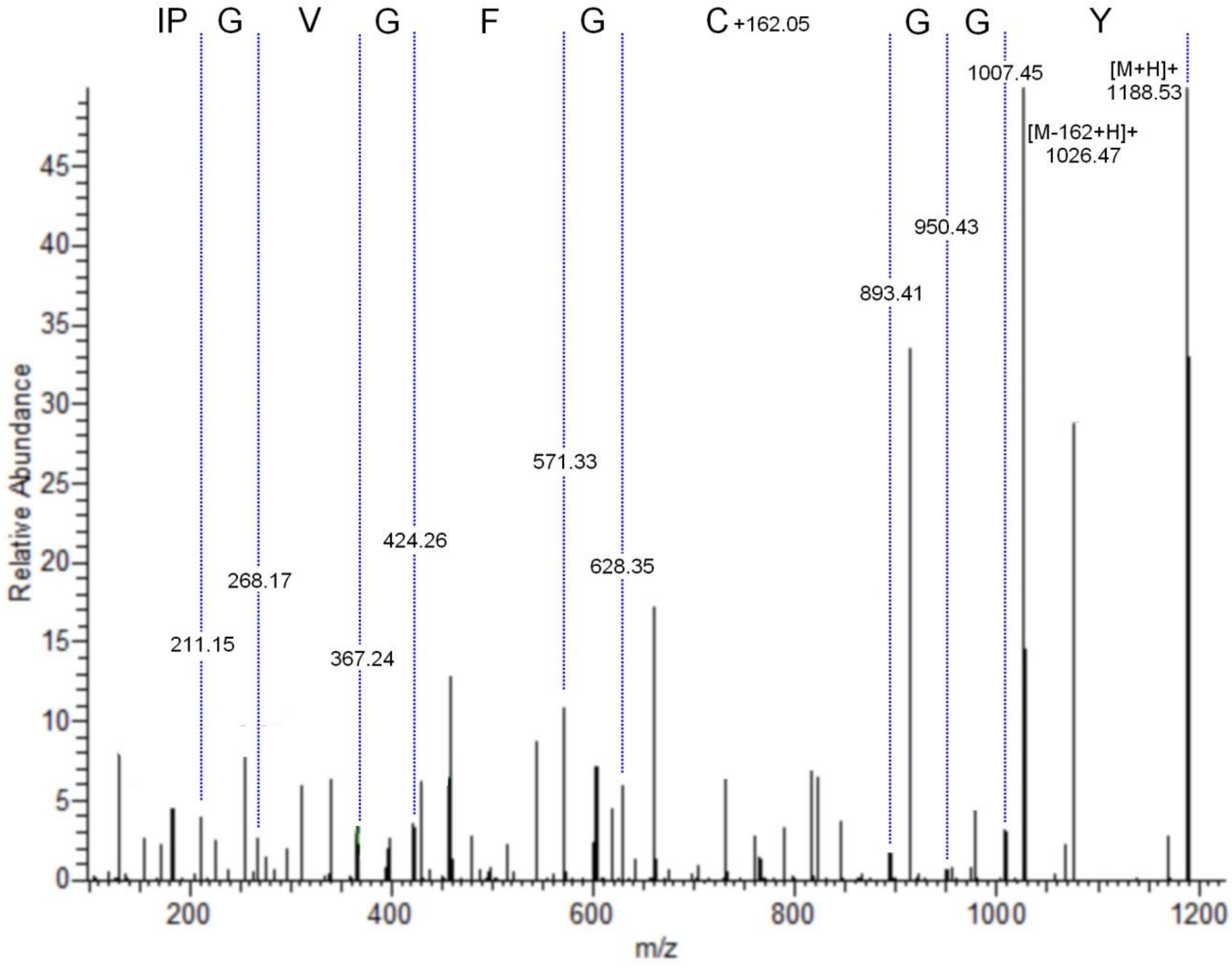

Supplementary Figure 7. ESI-Q-MSMS analysis of fragment IPGVGFGCGGY with indicated b ions after trial 2. The fragment was derived after pallidocin digestion with chymotrypsin. Theoretical monoisotopic mass $[M+H]^+$ of the sequence IPGVGFGCGGY is 1026.47, observed mass of the fragment is 1188.53. Fragment peaks of the peptide (1188.53) matching theoretical b ion masses of the IPGVGFGCGGY are indicated in the figure. The results confirm that Cys25 has a +162.05 posttranslational modification.

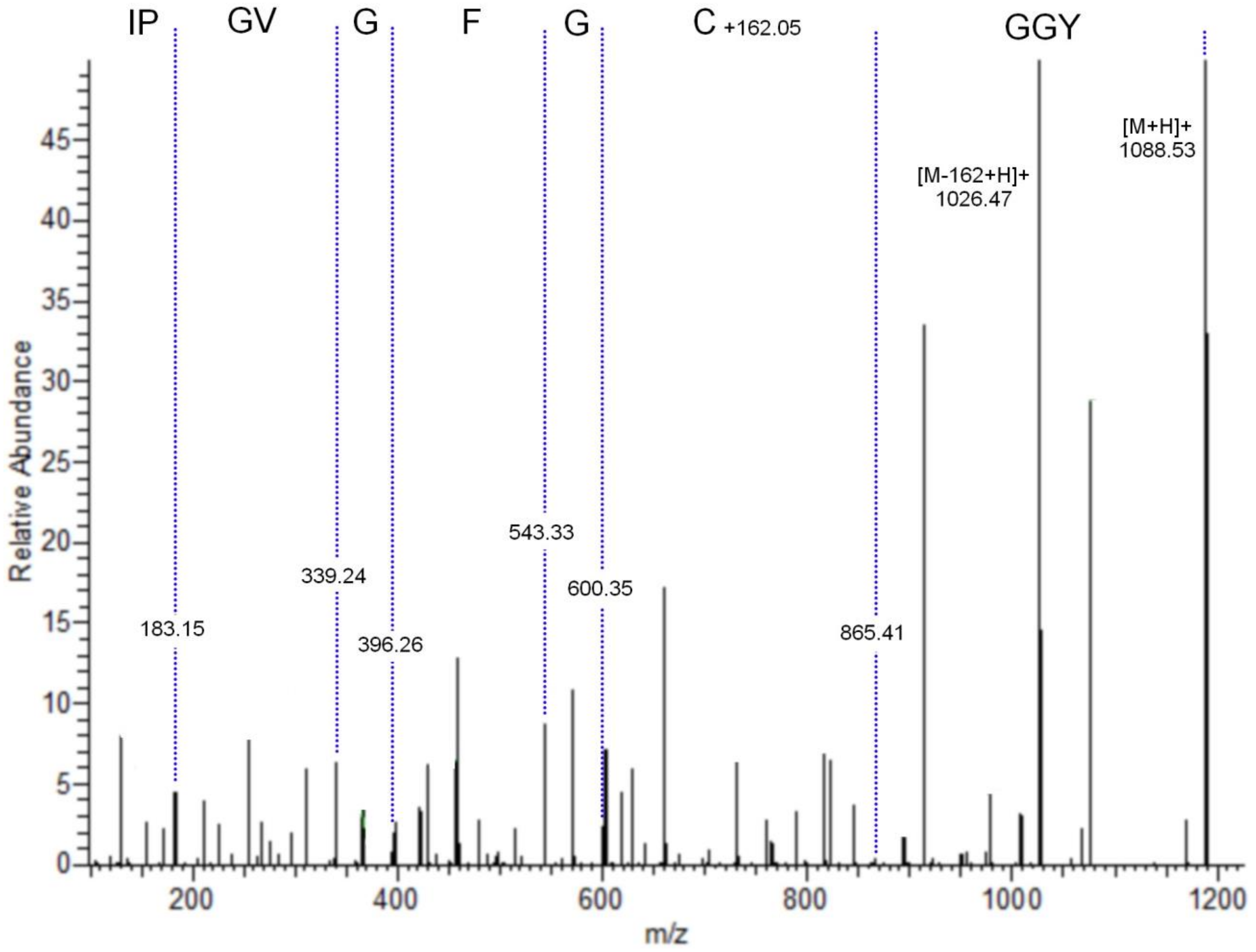

Supplementary Figure 8. ESI-Q-MSMS analysis of fragment IPGVGFGCGGY with indicated ions after trial 2. The fragment was derived after pallidocin digestion with chymotrypsin. Theoretical monoisotopic mass $[M+H]^+$ of the sequence IPGVGFGCGGY is 1026.47, observed mass of the fragment is 1188.53. Fragment peaks of the peptide (1188.53) matching theoretical ion masses of the IPGVGFGCGGY are indicated in the figure. The results confirm that Cys25 has a +162.05 posttranslational modification.

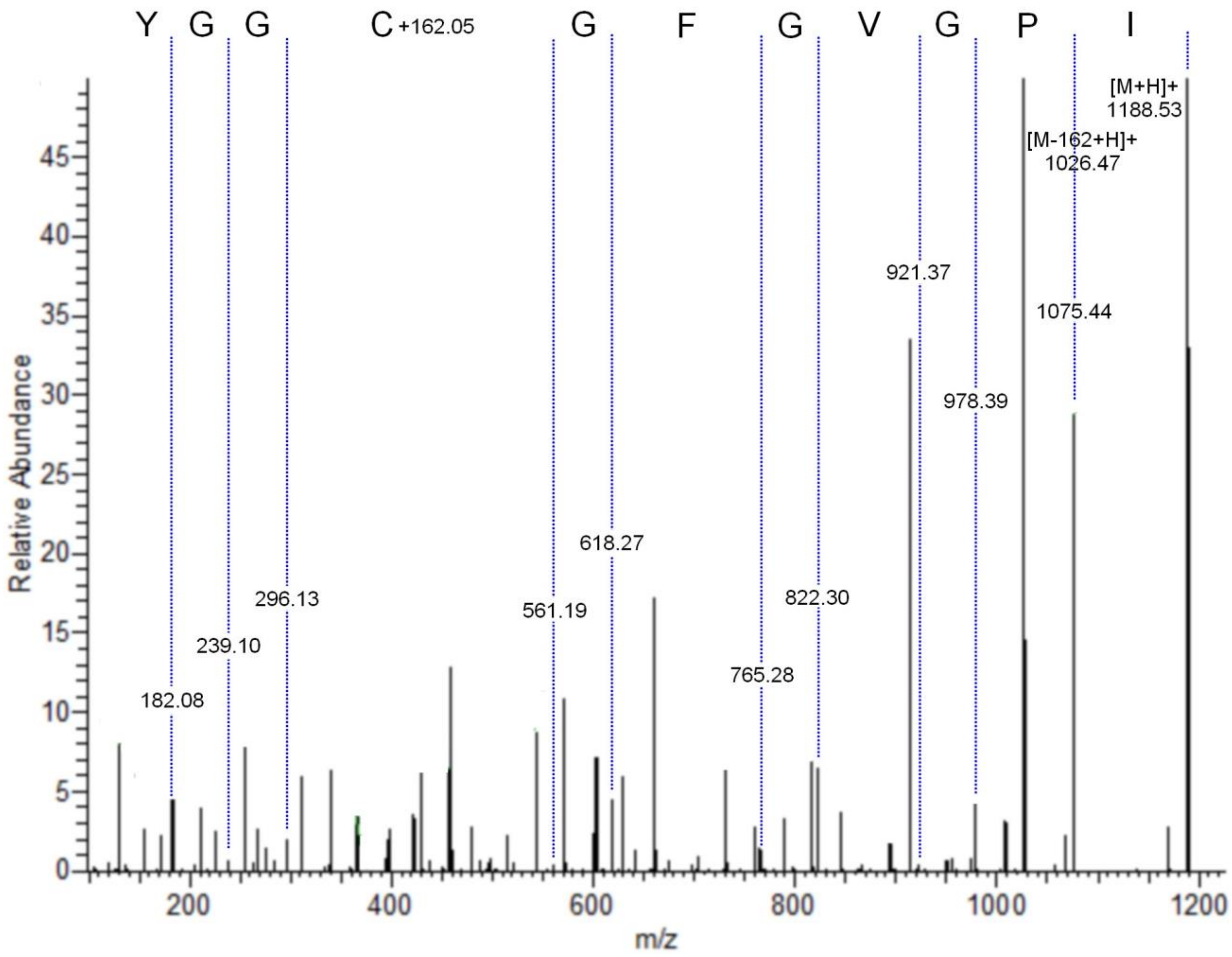

Supplementary Figure 9. ESI-Q-MSMS analysis of fragment IPGVGFGCGGY with indicated y ions after trial 2. The fragment was derived after pallidocin digestion with chymotrypsin. Theoretical monoisotopic mass $[M+H]^+$ of the sequence IPGVGFGCGGY is 1026.47, observed mass of the fragment is 1188.53. Fragment peaks of the peptide (1188.53) matching theoretical y ion masses of the IPGVGFGCGGY are indicated in the figure. The results confirm that Cys25 has a +162.05 posttranslational modification.

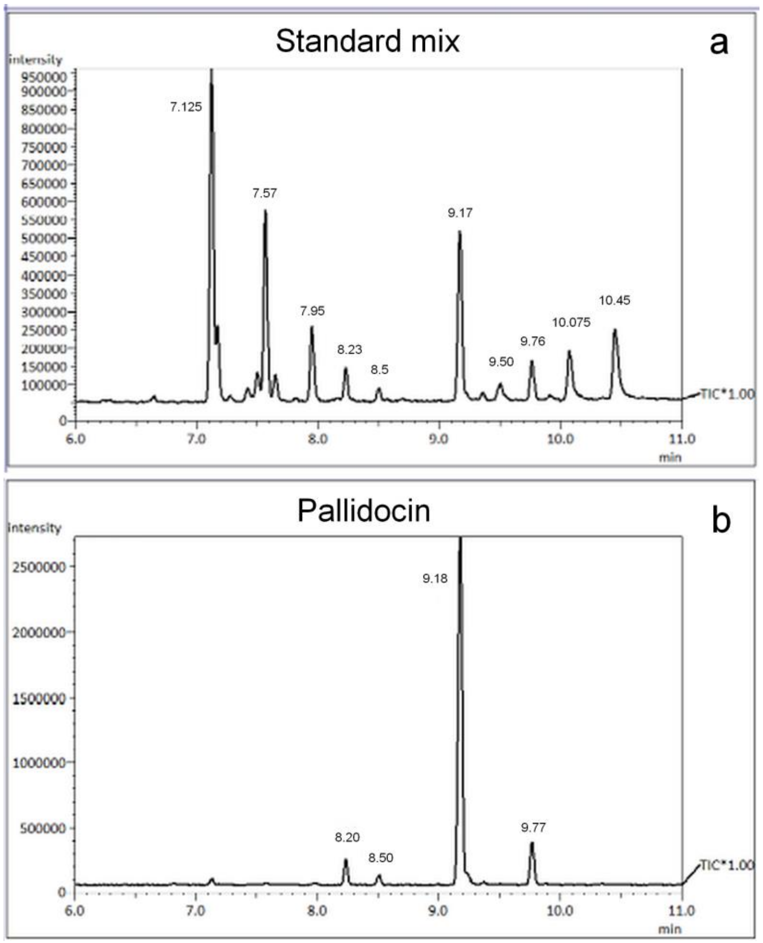

Supplementary Figure 10. Chromatogram of GC-MS analysis. (a) analysis of a standard mixture of sugars. Observed retention time of mannose – 7.125 min; α and β galactose – 7.57 min and 7.95 min; α and β glucose – 8.23 min and 8.5 min; mannitol (internal standard) – 9.17 min; mono-*O*-acetylated mannitol (internal standard) – 9.76 min; α and β N-acetylgalactosamine – 9.5 min and 10.075 min; N-acetylglucosamine – 10.45 min. Analysis of pallidocin is represented in (b).

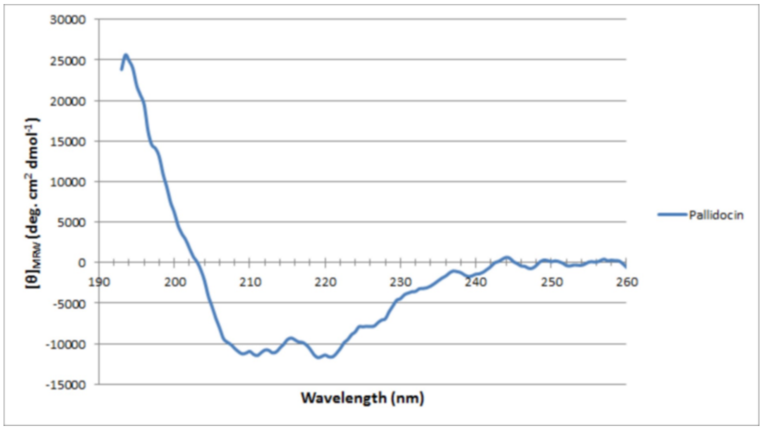

Supplementary Figure 11. Far UV circular dichroism spectroscopy data after analysis of mature pallidocin. The peptide was derived after leader cleavage of pre-Xa-PalA-His-Glc by Factor Xa peptidase. Source data are provided as a Source Data file.

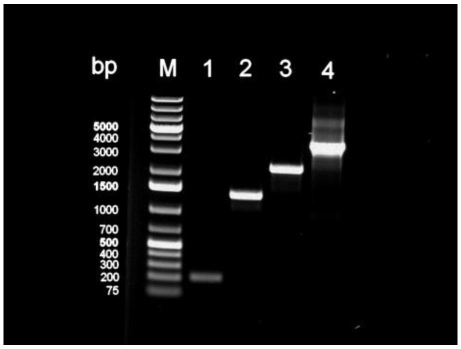

Supplementary Figure 12. DNA electrophoresis of PCR products amplified from constructs coding for pallidocin biosynthetic genes. Bp - base pairs, M - GeneRuler 1 kb Plus DNA Ladder (Thermo Fisher), 1 - *paA-his* amplified from pRSFDuet-1-palA-his, 2 - *palS* amplified from pBAD24-palS, 3 - *palT* amplified from pBAD24-palT, 4 - *palST* amplified from pBAD24-palST. Source data are provided as a Source Data file.

PalA, [M+H]+ avg. - 4064.75 Da, [M+H]+ mono. - 4061.76 Da

GYSAAQCAWMALSCVNYIPGVGFGCGGYSACELYKRYC
1 10 20 30 38

pre-PalA, [M+H]+ avg. - 6804.93 Da, [M+H]+ mono. - 6800.05 Da

MKDLLKELMYEVDLEEMENLQGSGYSAAQCAWMALSCVNYIPGVGFGCGGYSACELYKRYC
1 10 20 30 40 50 60 61

pre-His-Xa-PalA, [M+H]+ avg. - 8487.70 Da, [M+H]+ mono. - 8481.75 Da

MGSSHHHHHHSQDPMKDLLKELMYEVDLEEMENIEGRGYSAAQCAWMALSCVNYIPGVGFGCGGYSACELYKRYC
1 10 20 30 40 50 60 70 75

pre-PalA-His, [M+H]+ avg. - 7879.03 Da, [M+H]+ mono. - 7873.50 Da

MKDLLKELMYEVDLEEMENLQGSGYSAAQCAWMALSCVNYIPGVGFGCGGYSACELYKRYCGGHHHHHHH
1 10 20 30 40 50 60 70

PalA-His, [M+H]+ avg. - 5138.84 Da, [M+H]+ mono. - 5135.22 Da

GYSAAQCAWMALSCVNYIPGVGFGCGGYSACELYKRYCGGHHHHHHH
1 10 20 30 40 47

pre-SunA-His, [M+H]+ avg. - 6805.84 Da, [M+H]+ mono. - 6801.26 Da

MEKLFKEVKLEELENQKGSGLGKAQCAALWLQCASGGTIGCGGGAVACQNYRQFCRHHHHHH
1 10 20 30 40 50 60 62

pre-Hyp1-His, [M+H]+ avg. - 7917.14 Da, [M+H]+ mono. - 7911.55 Da

MNKNISKLMEEVSVEEMEQLQGKGLSKTQCAWMAASCVNYLPGVPGGFGCGGYEMCKEYKQYCNHHHHHH
1 10 20 30 40 50 60 70

pre-Hyp2-His, [M+H]+ avg. - 8519.43 Da, [M+H]+ mono. - 8513.82 Da

MDNLLREISEEDLELYDGGSGFSSAQCAYFIANCISGVGERRGCGSQQVDCMLARQCRQDQSPPYGGSRPAHHHHHH
1 10 20 30 40 50 60 70 77

Hyp1-His, [M+H]+ avg. - 5371.21 Da, [M+H]+ mono. - 5367.30 Da

MGLSKTQCAWMAASCVNYLPGVPGGFGCGGYEMCKEYKQYCNHHHHHH
1 10 20 30 40 48

Hyp2-His, [M+H]+ avg. - 6370.17 Da, [M+H]+ mono. - 6365.83 Da

MGFSSAQCAYFIANCISGVGERRGCGSQQVDCMLARQCRQDQSPPYGGSRPAHHHHHH
1 10 20 30 40 50 58

Supplementary Figure 13. Peptides sequences and their molecular weights. The masses are provided as average and monoisotopic in reduced peptide form. Amino acids in black determines the core peptide sequence, in brown – the leader peptide sequence, in red – the engineered additional amino acids, in blue – the engineered Factor Xa peptidase recognition site.

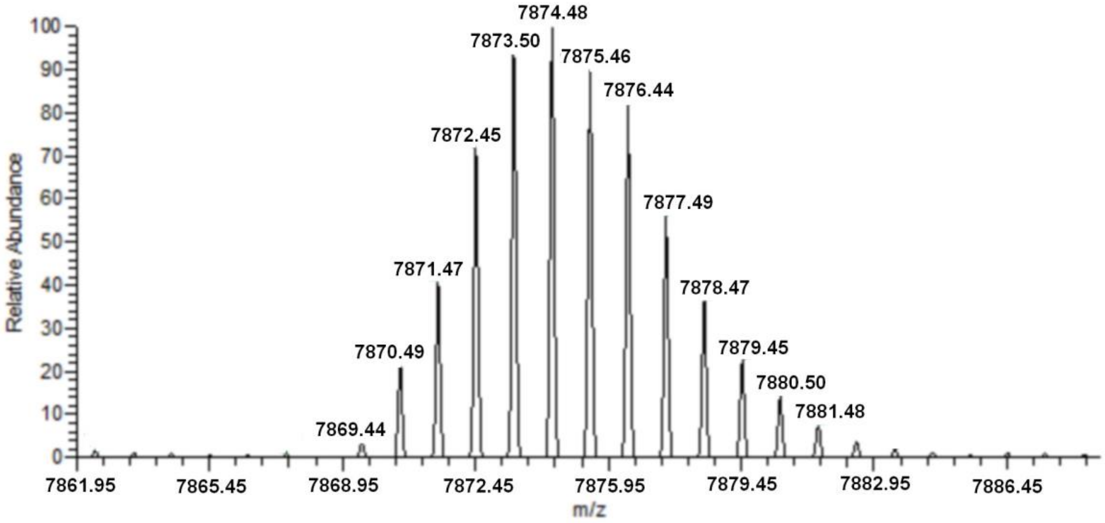

Supplementary Figure 14. LC-ESI-MS analysis of purified pre-PalA-His after individually expressed *palA-his*. Observed monoisotopic mass $[M+H]^+$ of the peptide (7869.44) matches predicted monoisotopic mass $[M+H]^+$ of pre-PalA-His with two disulfide bonds (7869.47).

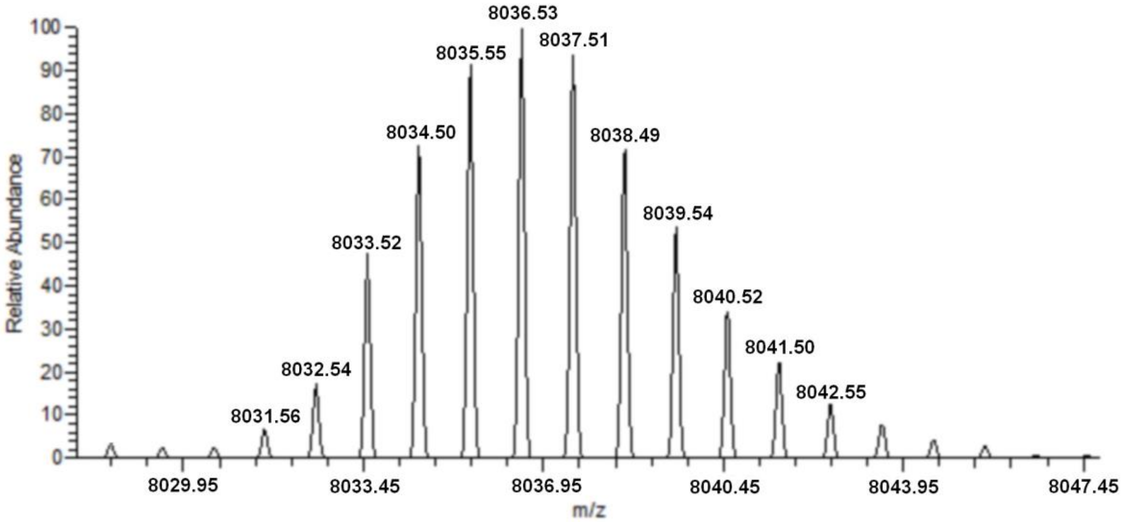

Supplementary Figure 15. LC-ESI-MS analysis of purified pre-PalA-His-Glc after *palA-his* co-expression with *palS*. Observed monoisotopic mass $[M+H]^+$ of the peptide (8031.56) matches predicted monoisotopic mass $[M+H]^+$ of pre-PalA-His-Glc with two disulfide bonds (8031.55).

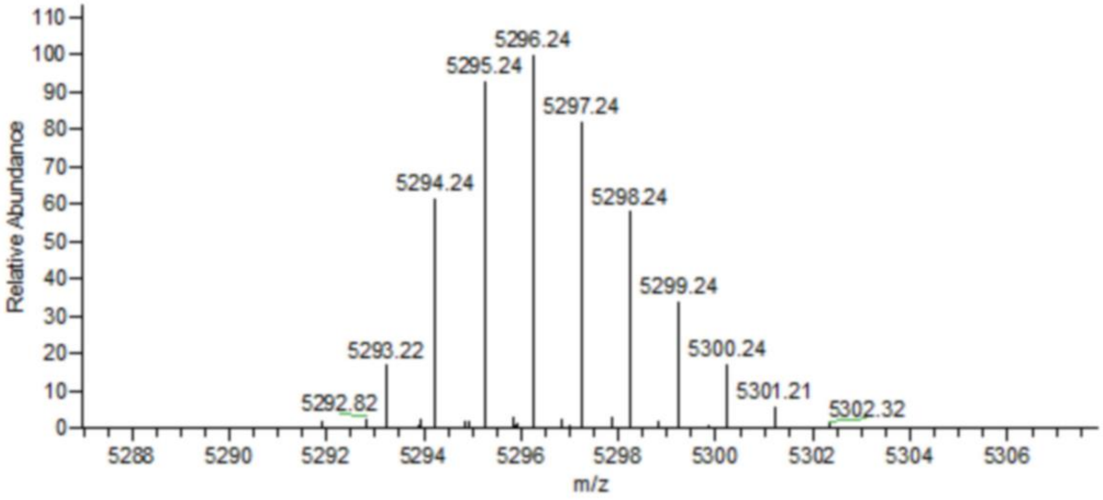

Supplementary Figure 16. LC-ESI-MS analysis of purified PalA-His-Glc after *palA-his* co-expression with *palST*. Observed monoisotopic mass $[M+H]^+$ of the peptide (5293.22) matches predicted monoisotopic mass $[M+H]^+$ of PalA-His-Glc with two disulfide bonds (5293.27), within error (-0.05).

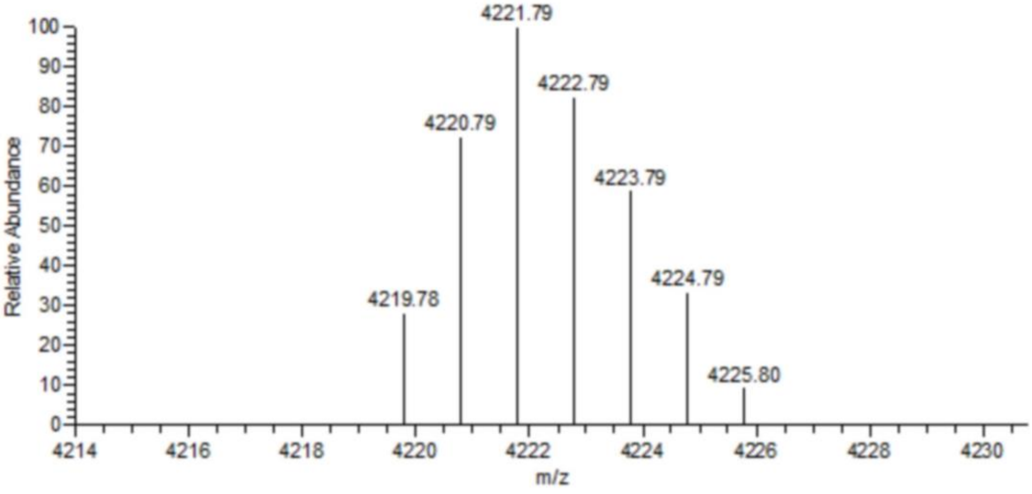

Supplementary Figure 17. Mass spectrometry analysis by LC-ESI-MS of PalA-Glc after *in vitro* leader cleavage of pre-His-Xa-PalA-Glc using Factor Xa peptidase. Predicted monoisotopic mass $[M+H]^+$ of PalA-Glc with two disulfide bonds is 4219.81. Observed monoisotopic mass $[M+H]^+$ is 4219.78. Results confirm that the leader sequence was cleaved, the remaining core peptide is glycosylated and has two disulfide bonds.

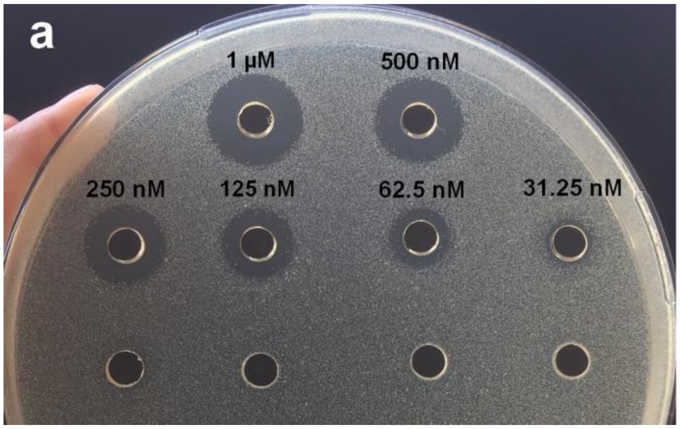

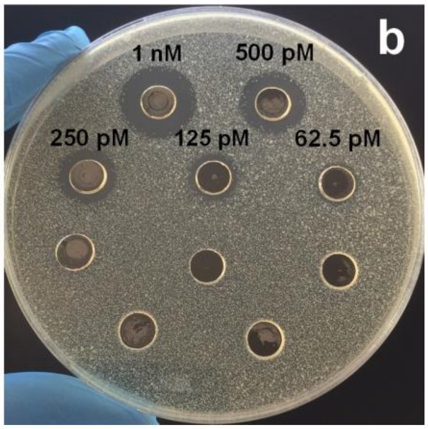

Supplementary Figure 18. Antibacterial activity of pre-His-Xa-PalA-Glc and mature pallidocin assessed by agar well dilutions assay against *P. genomospecies* 1 NUB36187. (a) antibacterial activity of pre-His-Xa-PalA-Glc. 1 µM of peptide in NB medium was diluted two-fold and assessed for activity. Peptide concentration of 31.25 nM still retains the activity. (b) antibacterial activity of mature pallidocin, which was derived after leader cleavage of pre-His-Xa-PalA-Glc. 1 nM of peptide in NB medium was diluted two-fold and assessed for activity. Peptide concentration of 62.5 pM still retains the activity. Results show that the removal of the leader enhances activity approximately 500 times.

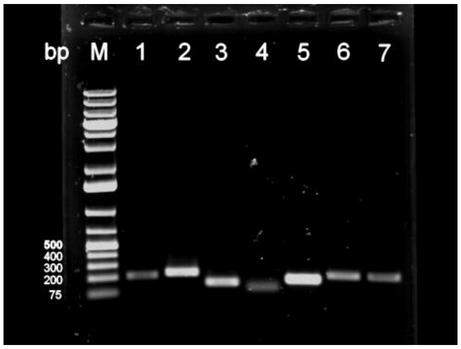

Supplementary Figure 19. DNA electrophoresis of PCR products amplified from constructs coding for precursor or core peptides. Bp - base pairs, M - GeneRuler 1 kb Plus DNA Ladder (Thermo Fisher), 1 - *sunA-his* amplified from pRSFDuet-1-sunA-his, 2 - *hyp2-his* amplified pRSFDuet-1-hyp2-his, 3 - *core_hyp2-his* amplified from pRSFDuet-1-core_hyp2-his, 4 - *core_hyp1-his* amplified from pRSFDuet-1-core_hyp1-his, 5 - *hyp1-his* amplified from pRSFDuet-1-hyp1-his, 6 - *enfA4-9-his* amplified from pRSFDuet-1-enfA4-9-his, 7 - *gccF-his* amplified from pRSFDuet-1-gccF-his. Source data are provided as a Source Data file.

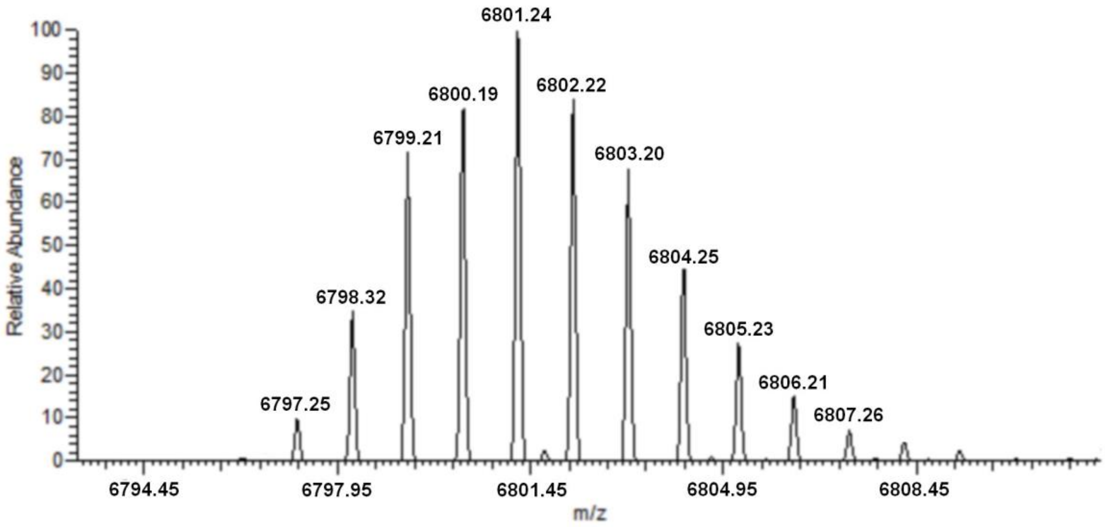

Supplementary Figure 20. LC-ESI-MS analysis of purified pre-SunA-His after individually expressed *sunA-his*. Observed monoisotopic mass $[M+H]^+$ of the peptide (6797.25) matches predicted monoisotopic mass $[M+H]^+$ of pre-SunA-His with two disulfide bonds (6797.23).

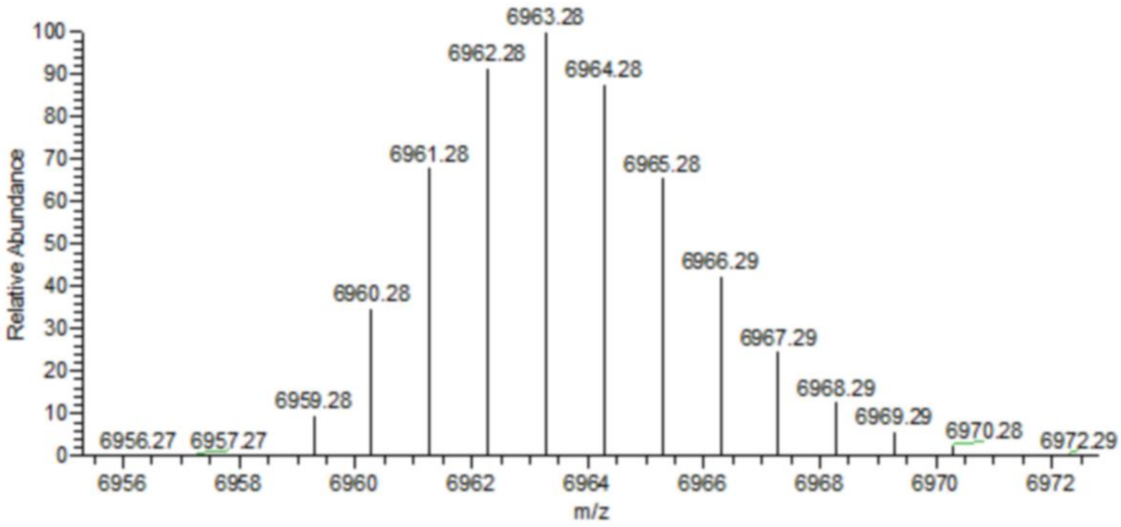

Supplementary Figure 21. LC-ESI-MS analysis of purified pre-SunA-His-Glc after *sunA-his* co-expression with *palS*. Observed monoisotopic mass $[M+H]^+$ of the peptide (6959.28) matches predicted monoisotopic mass $[M+H]^+$ of pre-SunA-His-Glc with two disulfide bonds (6959.31).

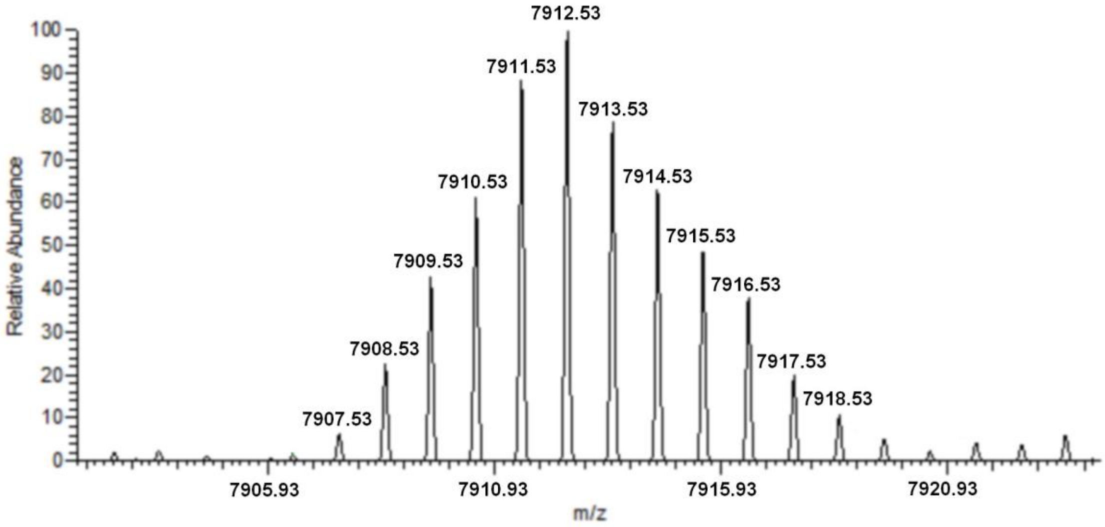

Supplementary Figure 22. LC-ESI-MS analysis of purified pre-Hyp1-His after individually expressed *hyp1-his*. Observed monoisotopic mass $[M+H]^+$ of the peptide (7907.53) matches predicted monoisotopic mass $[M+H]^+$ of pre-Hyp1-His with two disulfide bonds (7907.52).

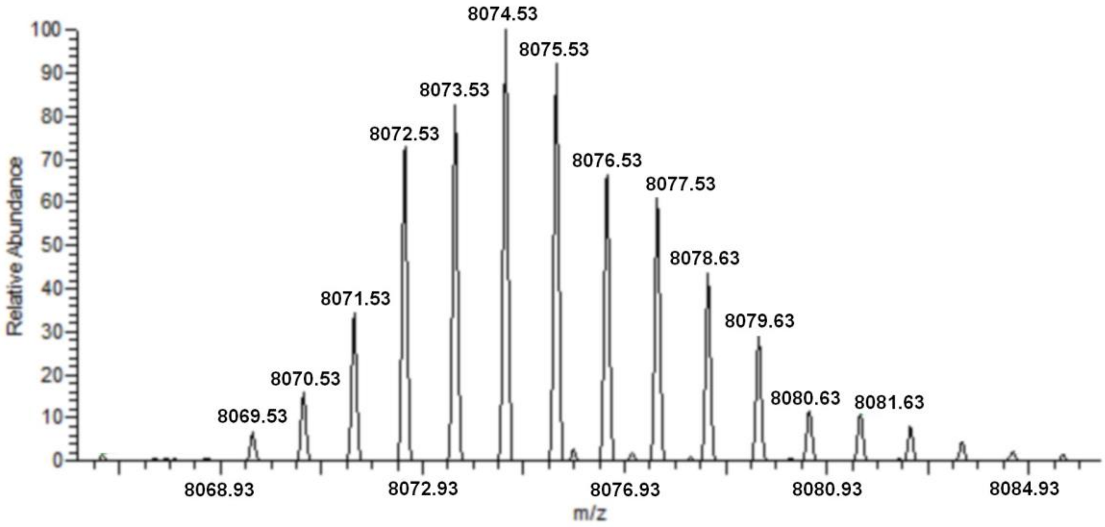

Supplementary Figure 23. LC-ESI-MS analysis of purified pre-Hyp1-His-Glc after *hyp1-his* co-expression with *palS*. Observed monoisotopic mass $[M+H]^+$ of the peptide (8069.53) matches predicted monoisotopic mass $[M+H]^+$ of pre-Hyp1-His-Glc with two disulfide bonds (8069.57).

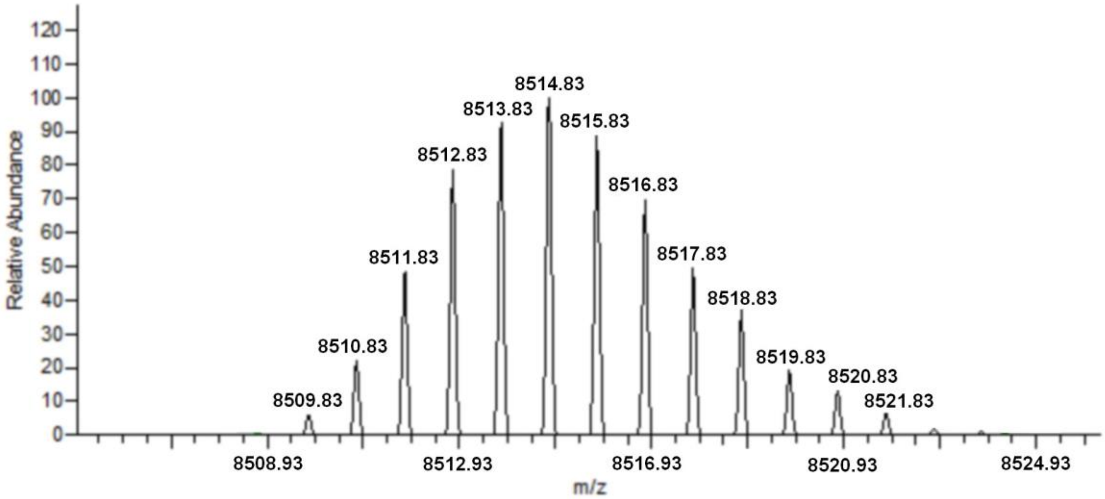

Supplementary Figure 24. LC-ESI-MS analysis of purified pre-Hyp2-His after individually expressed *hyp2-his*. Observed monoisotopic mass $[M+H]^+$ of the peptide (8509.83) matches predicted monoisotopic mass $[M+H]^+$ of pre-Hyp2-His with two disulfide bonds (8509.79).

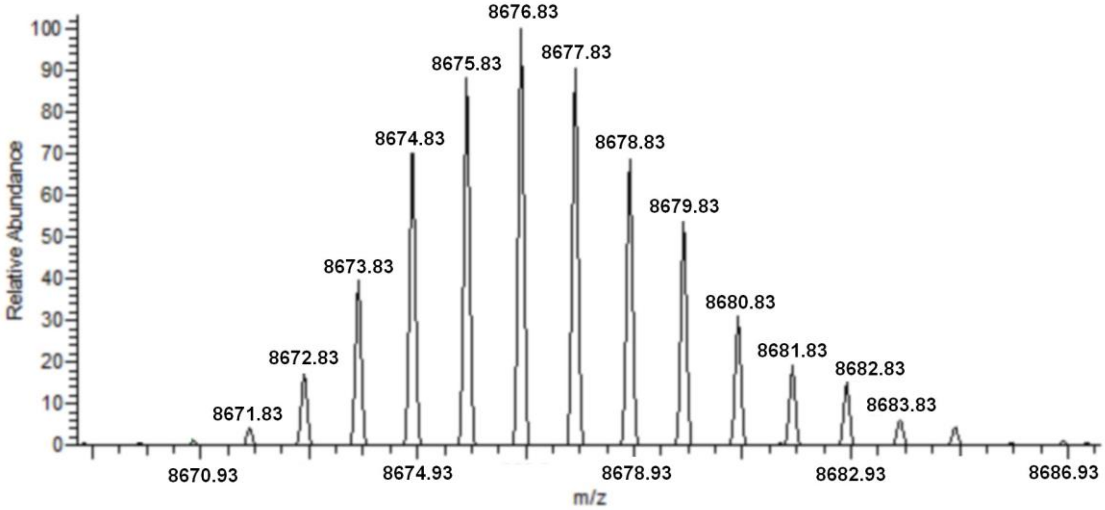

Supplementary Figure 25. LC-ESI-MS analysis of purified pre-Hyp2-His-Glc after *hyp2-his* co-expression with *palS*. Observed monoisotopic mass $[M+H]^+$ of the peptide (8671.83) matches predicted monoisotopic mass $[M+H]^+$ of pre-Hyp2-His-Glc with two disulfide bonds (8671.84).

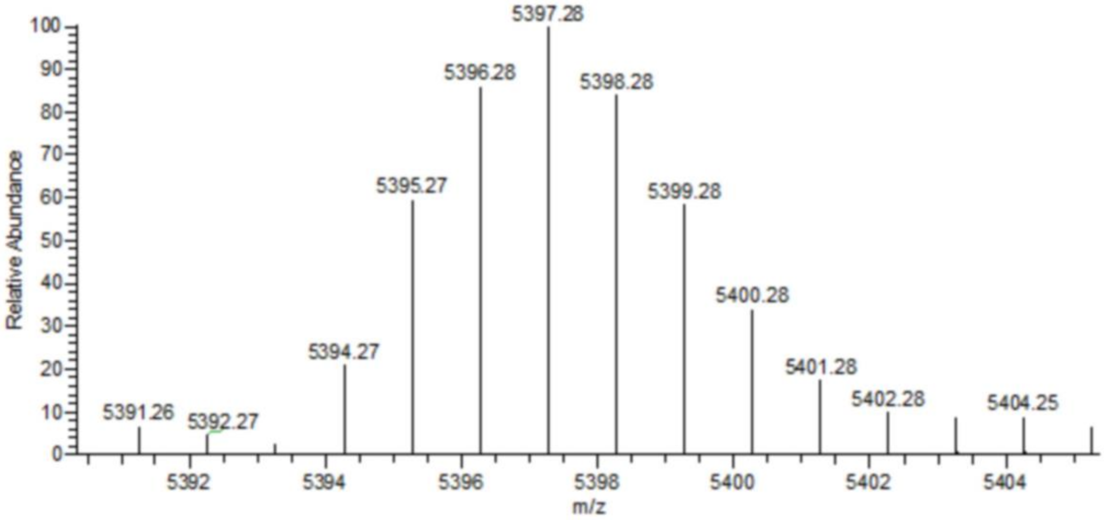

Supplementary Figure 26. LC-ESI-MS analysis of purified Hyp1-His-Glc after *core_hyp1-his* co-expression with *palS*. Observed monoisotopic mass $[M+H]^+$ of the peptide (5394.27) matches predicted monoisotopic mass $[M+H]^+$ of Hyp1-His-Glc core peptide with two disulfide bonds and cleaved Met1 (5394.31), within error (-0.04).

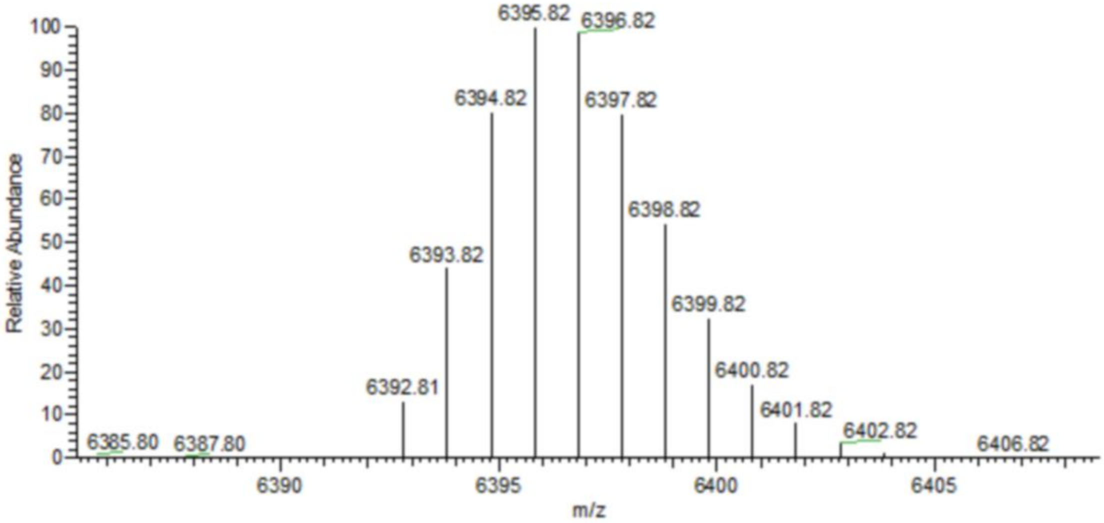

Supplementary Figure 27. LC-ESI-MS analysis of purified Hyp2-His-Glc after *core_hyp2-his* co-expression with *palS*. Observed monoisotopic mass $[M+H]^+$ of the peptide (6392.81) matches predicted monoisotopic mass $[M+H]^+$ of Hyp2-His-Glc core peptide with two disulfide bonds and cleaved Met1 6392.84), within error (-0.03).

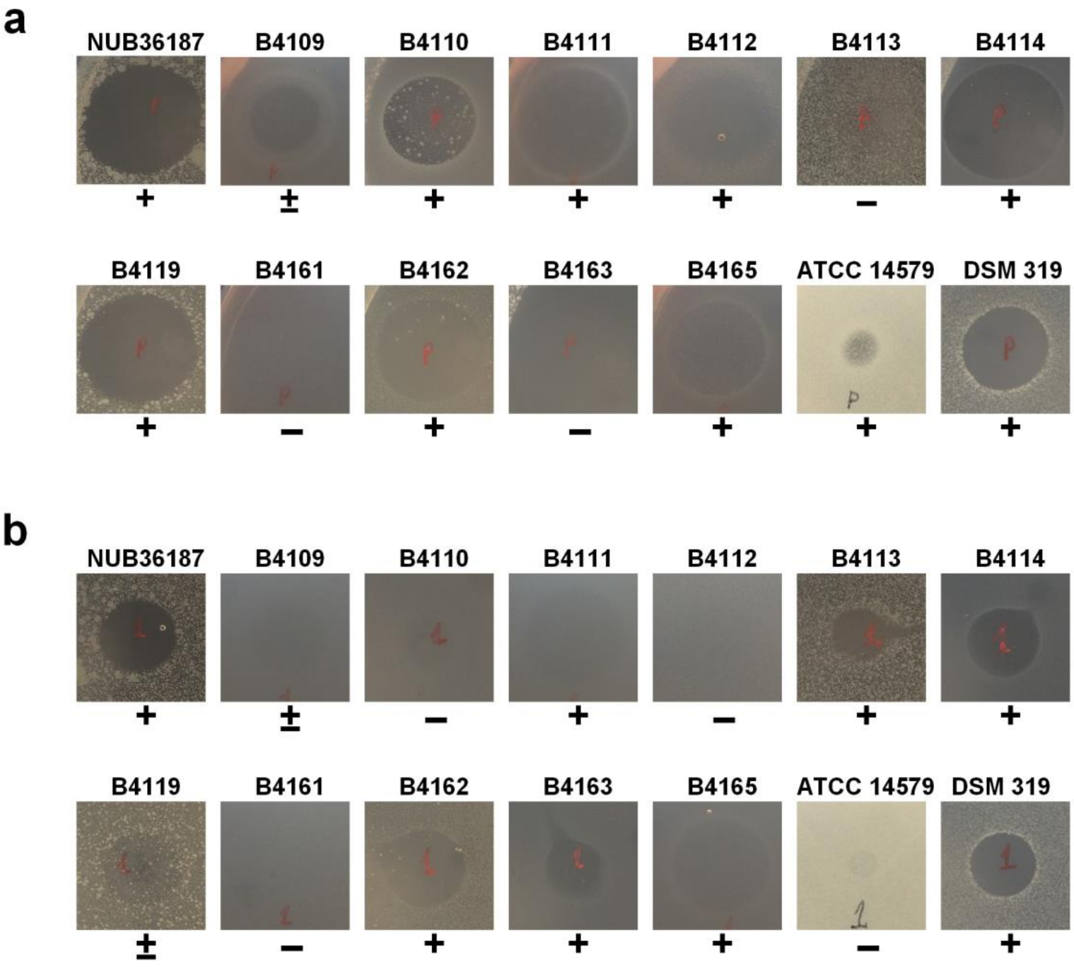

Supplementary Figure 28. Antibacterial activity spectrum of pallidocin and Hyp1. (a) antibacterial activity spectrum of pallidocin. (b) antibacterial activity spectrum of Hyp1-His. Plus indicates clearly visible antibacterial activity of the peptide against the indicator strain, plus with minus indicates very weak activity of the peptide against the indicator strain, minus indicates no activity of the peptide against the indicator strain. A spot on a lawn assay was used for antibacterial activity determination against *Geobacillus stearothermophilus* B4109, B4111, B4112, B4114, B4161, B4163, *Geobacillus* sp. B4113, *Parageobacillus genomospecies* 1 NUB36187, *P. toebii* B4110, B4162, *P. caldoxylosilyticus* B4119, *Caldibacillus debilis* B4165, *Bacillus cereus* ATCC 14579 and *B. megaterium* DSM 319 strains.

Supplementary Table 1. Effects of pH and temperature on the activity of pallidocin.

Temperature/pH treatment	Residual activity after treatment
Room temperature, 24 h.	+++
Room temperature, 10 days.	+++
Room temperature, 30 days	+
60˚C, 3 h.	+++
90˚C, 3 h.	+++
121˚C, 15 min.	++
pH 2, 3 h.	+++
pH 4, 3 h.	+++
pH 6, 3 h.	+++
pH 8, 3 h.	+++
pH 10, 3 h.	+++

Note: "+++" refers to 100% residual activity, "++" refers to 40-60% residual activity, "+" refers to 10-20% residual activity.

Supplementary Table 2. The role of disulfide bonds on activity of pallidocin precursors and core peptides.

Treatment of the peptide	pre-PalA-His		pre-PalA-His-Glc		PalA-His-Glc	
	Antibacterial activity	Cys alkylation	Antibacterial activity	Cys alkylation	Antibacterial activity	Cys alkylation
No treatment	-	-	+	-	+	-
IAA	-	+ (1 Cys)	+	-	+	-
TCEP and IAA	-	+ (5 Cys)	-	+ (4 Cys)	-	+ (4 Cys)

Note: peptides were derived after precursor co-expression with PalS or PalST. pre-PalA-His – pallidocin precursor with leader; pre-PalA-Glc – glycosylated pallidocin precursor with leader; Pal-His-Glc – glycosylated pallidocin core peptide. Legends: "-" peptide is not active or no Cys alkylated, respectively; "+" peptide is active or Cys alkylated, respectively.

Supplementary Table 3. The role of disulfide bonds on activity of Hyp1 precursors and core peptides.

Treatment of the peptide	Hyp1-His		pre-Hyp1-His-Glc		Hyp1-His-Glc	
	Antibacterial activity	Cys alkylation	Antibacterial activity	Cys alkylation	Antibacterial activity	Cys alkylation
No treatment	-	-	+	-	+	-
IAA	-	+ (1 Cys)	+	-	+	-
TCEP and IAA	-	+ (5 Cys)	-	+ (4 Cys)	-	ND

Note: peptides were derived after precursor or core peptide co-expression with PalS. pre-Hyp1-His – Hyp1 precursor with leader; pre-Hyp1-Glc – glycosylated Hyp1 precursor with leader; Hyp1-His-Glc – glycosylated Hyp1 core peptide. Legends: "-" peptide is not active or no Cys alkylated, respectively; "+" peptide is active or Cys alkylated, respectively; ND – not determined.

Supplementary Table 4. The role of disulfide bonds on activity of Hyp2 precursors and core peptides.

Treatment of the peptide	pre-Hyp2-His		pre-Hyp2-His-Glc		Hyp2-His-Glc	
	Antibacterial activity	Cys alkylation	Antibacterial activity	Cys alkylation	Antibacterial activity	Cys alkylation
No treatment	-	-	-	-	-	-
IAA	ND	+ (1 Cys)	ND	-	ND	-
TCEP and IAA	ND	+ (5 Cys)	ND	+ (4 Cys)	ND	+ (4 Cys)

Note: peptides were derived after precursor or core peptide co-expression with PalS. pre-Hyp2-His – Hyp2 precursor with leader; pre-Hyp2-Glc – glycosylated Hyp2 precursor with leader; Hyp2-His-Glc – glycosylated Hyp2 core peptide. Legends: "-" peptide is not active or no Cys alkylated, respectively; "+" peptide is active or Cys alkylated, respectively; ND – not determined.

Supplementary Table 5. MIC values of pallidocin for *Bacillus megaterium* DSM 319 and *Parageobacillus toebii* B4162.

Pallidocin MIC for *Bacillus megaterium* DSM 319

	1	2	3	4	5	6	7	8	9	10	Repli
A	592.1 nmol/L	296.1 nmol/L	148.03 nmol/L	74.0 nmol/L	37.0 nmol/L	18.5 nmol/L	9.3 nmol/L	4.6 nmol/L	2.3 nmol/L	1.2 nmol/L	1
B											
C	592.1 nmol/L	296.1 nmol/L	148.03 nmol/L	74.0 nmol/L	37.0 nmol/L	18.5 nmol/L	9.3 nmol/L	4.6 nmol/L	2.3 nmol/L	1.2 nmol/L	2
D											
E	592.1 nmol/L	296.1 nmol/L	148.03 nmol/L	74.0 nmol/L	37.0 nmol/L	18.5 nmol/L	9.3 nmol/L	4.6 nmol/L	2.3 nmol/L	1.2 nmol/L	3
F											

Pallidocin MIC for *Geobacillus toebii* 4162 strain

	1	2	3	4	5	6	7	8	9	10	Repli
A	4.0 nmol/L	2.0 nmol/L	985.3 pmol/L	492.7 pmol/L	246.3 pmol/L	123.4 pmol/L	61.9 pmol/L	30.8 pmol/L	15.4 pmol/L	7.7 pmol/L	1
B											
C	4.0 nmol/L	2.0 nmol/L	985.3 pmol/L	492.7 pmol/L	246.3 pmol/L	123.4 pmol/L	61.9 pmol/L	30.8 pmol/L	15.4 pmol/L	7.7 pmol/L	2
D											
E	4.0 nmol/L	2.0 nmol/L	985.3 pmol/L	492.7 pmol/L	246.3 pmol/L	123.4 pmol/L	61.9 pmol/L	30.8 pmol/L	15.4 pmol/L	7.7 pmol/L	3
F											
G	4.0 nmol/L	2.0 nmol/L	985.3 pmol/L	492.7 pmol/L	246.3 pmol/L	123.4 pmol/L	61.9 pmol/L	30.8 pmol/L	15.4 pmol/L	7.7 pmol/L	4
H											

Legends: growth inhibition | no growth inhibition

Two-fold dilutions of pallidocin were made in NB medium and dispersed in 96 well plate. The bacterial growth in the wells was evaluated after overnight incubation (see materials and methods) and the lowest bacteriocin concentration inhibiting the growth (MIC) was determined (red). Source data are provided as a Source Data file.

Supplementary Table 6. MIC values of pallidocin for *Geobacillus stearothermophilus* B4114 and *Parageobacillus caldoxylosilyticus* B4119.

Pallidocin MIC for *Geobacillus stearothermophilus* 4114 strain

	1	2	3	4	5	6	7	8	9	10	Replicate
A	4.0 nmol/L	2.0 nmol/L	985.3 pmol/L	492.7 pmol/L	**246.3 pmol/L**	123.4 pmol/L	61.9 pmol/L	30.8 pmol/L	15.4 pmol/L	7.7 pmol/L	1
B											
C	4.0 nmol/L	2.0 nmol/L	985.3 pmol/L	492.7 pmol/L	**246.3 pmol/L**	123.4 pmol/L	61.9 pmol/L	30.8 pmol/L	15.4 pmol/L	7.7 pmol/L	2
D											
E	4.0 nmol/L	2.0 nmol/L	985.3 pmol/L	**492.7 pmol/L**	246.3 pmol/L	123.4 pmol/L	61.9 pmol/L	30.8 pmol/L	15.4 pmol/L	7.7 pmol/L	3
F											

Pallidocin MIC for *Parageobacillus caldoxylosilyticus* 4119 strain

	1	2	3	4	5	6	7	8	9	10	Replicate
A	4.0 nmol/L	2.0 nmol/L	**985.3 pmol/L**	492.7 pmol/L	246.3 pmol/L	123.4 pmol/L	61.9 pmol/L	30.8 pmol/L	15.4 pmol/L	7.7 pmol/L	1
B											
C	4.0 nmol/L	2.0 nmol/L	**985.3 pmol/L**	492.7 pmol/L	246.3 pmol/L	123.4 pmol/L	61.9 pmol/L	30.8 pmol/L	15.4 pmol/L	7.7 pmol/L	2
D											
E	4.0 nmol/L	2.0 nmol/L	**985.3 pmol/L**	492.7 pmol/L	246.3 pmol/L	123.4 pmol/L	61.9 pmol/L	30.8 pmol/L	15.4 pmol/L	7.7 pmol/L	3
F											
G	4.0 nmol/L	2.0 nmol/L	**985.3 pmol/L**	492.7 pmol/L	246.3 pmol/L	123.4 pmol/L	61.9 pmol/L	30.8 pmol/L	15.4 pmol/L	7.7 pmol/L	4
H											

Legends: growth inhibition | no growth inhibition

Two-fold dilutions of pallidocin were made in NB medium and dispersed in 96 well plate. The bacterial growth in the wells was evaluated after overnight incubation (see materials and methods) and the lowest bacteriocin concentration inhibiting the growth (MIC) was determined (red). Source data are provided as a Source Data file.

Supplementary Table 7. MIC values of pallidocin for *Parageobacillus genomospecies* 1 NUB36187.

Pallidocin MIC for *Parageobacillus genomospecies* 1 NUB36187

	1	2	3	4	5	6	7	8	9	10	Replicate
A	1.2 µmol/L	590.0 nmol/L	295.0 nmol/L	147.5 nmol/L	73.8 nmol/L	36.9 nmol/L	18.4 nmol/L	9.2 nmol/L	4.6 nmol/L	2.3 nmol/L	1
B	1.2 nmol/L	576.2 pmol/L	288.1 pmol/L	144.0 pmol/L	72.0 pmol/L	36.0 pmol/L	18.0 pmol/L	9.0 pmol/L	4.5 pmol/L	**2.3 pmol/L**	
C	1.2 µmol/L	590.0 nmol/L	295.0 nmol/L	147.5 nmol/L	73.8 nmol/L	36.9 nmol/L	18.4 nmol/L	9.2 nmol/L	4.6 nmol/L	2.3 nmol/L	2
D	1.2 nmol/L	576.2 pmol/L	288.1 pmol/L	144.0 pmol/L	72.0 pmol/L	36.0 pmol/L	18.0 pmol/L	9.0 pmol/L	4.5 pmol/L	**2.3 pmol/L**	
E	1.2 µmol/L	590.0 nmol/L	295.0 nmol/L	147.5 nmol/L	73.8 nmol/L	36.9 nmol/L	18.4 nmol/L	9.2 nmol/L	4.6 nmol/L	2.3 nmol/L	3
F	1.2 nmol/L	576.2 pmol/L	288.1 pmol/L	144.0 pmol/L	72.0 pmol/L	36.0 pmol/L	**18.0 pmol/L**	9.0 pmol/L	4.5 pmol/L	2.3 pmol/L	

Pallidocin MIC for *Parageobacillus genomospecies* 1 NUB36187

	1	2	3	4	5	6	7	8	9	10	Replicate
A	39.5 nmol/L	19.7 nmol/L	9.9 nmol/L	4.9 nmol/L	2.5 nmol/L	1.2 nmol/L	615.8 pmol/L	307.9 pmol/L	154.2 pmol/L	77.1 pmol/L	1
B	38.6 pmol/L	19.3 pmol/L	9.6 pmol/L	4.8 pmol/L	**2.4 pmol/L**	1.2 pmol/L	601.6 fmol/L	300.8 fmol/L	150.6 fmol/L	75.3 fmol/L	
C	39.5 nmol/L	19.7 nmol/L	9.9 nmol/L	4.9 nmol/L	2.5 nmol/L	1.2 nmol/L	615.8 pmol/L	307.9 pmol/L	154.2 pmol/L	77.1 pmol/L	2
D	38.6 pmol/L	19.3 pmol/L	9.6 pmol/L	**4.8 pmol/L**	2.4 pmol/L	1.2 pmol/L	601.6 fmol/L	300.8 fmol/L	150.6 fmol/L	75.3 fmol/L	
E	39.5 nmol/L	19.7 nmol/L	9.9 nmol/L	4.9 nmol/L	2.5 nmol/L	1.2 nmol/L	615.8 pmol/L	307.9 pmol/L	154.2 pmol/L	77.1 pmol/L	3
F	38.6 pmol/L	19.3 pmol/L	**9.6 pmol/L**	4.8 pmol/L	2.4 pmol/L	1.2 pmol/L	601.6 fmol/L	300.8 fmol/L	150.6 fmol/L	75.3 fmol/L	
G	39.5 nmol/L	19.7 nmol/L	9.9 nmol/L	4.9 nmol/L	2.5 nmol/L	1.2 nmol/L	615.8 pmol/L	307.9 pmol/L	154.2 pmol/L	**77.1 pmol/L**	4
H	38.6 pmol/L	19.3 pmol/L	9.6 pmol/L	4.8 pmol/L	2.4 pmol/L	1.2 pmol/L	601.6 fmol/L	300.8 fmol/L	150.6 fmol/L	75.3 fmol/L	

Legends: growth inhibition | no growth inhibition

Two-fold dilutions of pallidocin were made in NB medium and dispersed in 96 well plate. The bacterial growth in the wells was evaluated after overnight incubation (see materials and methods) and the lowest bacteriocin concentration inhibiting the growth (MIC) was determined (red). Source data are provided as a Source Data file.

Supplementary Table 8. The list of primers.

**Primer name**	**Primer sequence (5' to 3')**
F-PalA-USER	ATTCACCAUGAAAGATTTATTAAAAGAATTAATGTATGAG
R-PalA-USER	AAACAGCCUCGGTATATCGGTTTCTTTTTTTCATG
F-pBAD-USER	AGGCTGTTUTGGCGGATGAG
R-pBAD-USER	ATGGTGAAUTCCTCCTGCTAG
F-PalA-BspHI	ATTCGTCATGAAAGATTTATTAAAAGAATTAATGTATGAG
R-PalA-HindIII	TTTTAAAGCTTAACAATAACGTTTATACAATTCGC
F-PalS-In-Fusion	CAGGAGGAATTCACCATGGGGAACTTAAGAGATTTTTATCAAC
R-PalS-In-Fusion	CAAAACAGCCAAGCTTGTTAAATTTTATTTATACTATCTATAAAACTTAATAGATTATC
F-PalT-In-Fusion	CAGGAGGAATTCACCATGATTTTAAGGAAATTTGCACATGTAAGAC
R-PalT-In-Fusion	CAAAACAGCCAAGCTTGTCATAGGACCTCTACCTCCAGATTTC
F-BdbA	AGTGCTATATTTTTAACACTTGGAG
R-BdbA	ATCTTTAAGCATTTCTTCTGTTATTACTTC
F-BdbB	AATTTCTTTGATTGCTTTAATTAAGAGAG
R-BdbB	CTGCTGCACTAAAATATTATTTACACTC
F-PalA-His	CATCATCATCATCATTAAGCTTGCGGCCGCATAATG
R-PalA-His	ATGATGTCCTCCACAATAACGTTTATACAATTCGCAAG
F-SunA-BamHI	AAGGAGGGATCCGATGGAGAAGCTGTTCAAAGAAGTGAAGC
R-SunA-HindIII	CTAGATAAGCTTAACGGCAAAATTGACGGTAGTTCTG
F-GccF	ACCATGAGCAAGCTGGTGAAAACCCTG
R-GccF	TGGTGGCAGTGGTAGCTGCTGCTG
F-EnfA4-9	ATGGGTAACAGCATCCTGAACAAGATGACC
R-EnfA4-9	CAGGTACGGTTGGTTTGGCCT
R-Leaderless	CATGGTATATCTCCTTATTAAAGTTAAACAAAATTATTTCTACAGGG
F-Hyp1-Leaderless	GGTCTGAGCAAAACCCAATGC
R-Hyp1	ATAATAAAGCTTAATTGCAGTATTGTTTATACTCTTTGCACATTTCG
F-Hyp2-Leaderless	GGTTTCAGCAGCGCGCAGT
F-Hyp2	ATGGACAACCTGCTGCGTGAG
R-Hyp2	GCTTTGATCCTGACGGCATTGACG
F-PalA-BamHI	GAAAAGGATCCGATGAAAGATTTATTAAAAGAATTAATGTATGAGGTAGATTTAGAAGA
R-PalA-HindIII[2]	TAATTTAAGCTTAACAATAACGTTTATACAATTCGCAAGCGC
F-PalA-Xa	GGATATTCAGCTGCCCAATGTGCATG
R-PalA-Xa	ACGACCTTCGATATTCTCCATTTCTTCTAAATCTACCTCATAC
F-pBAD24	GATTATTTGCACGGCGTCAC
R-pBAD24	GGCGGATTTGTCCTACTCAG
F-pRSFDuet-1	GGATCTCGACGCTCTCCCT
R-pRSFDuet-1	GATTATGCGGCCGTGTACAA
Pal0	GGATATTCAGCTGCCCAATG
Pal1	AGGAGTGGTTAGTCCTACAG
Pal2	ATAAATCCATCACATGATCATATC
Pal3	ATCTAAATTAAAAAACAATTATAGAAAACG
Pal4	TAGATGCCGGCTTACAAC
Pal5	ACTAGGACAGGCTCAAAGAC
Pal6	GGGTGTCGAAAGGAAAC
