# Supplementary material for: Heterologous biosynthesis and characterization of a glycocin from a thermophilic bacterium
Source: Nat Commun. 2019 Mar 7;10:1115. doi: 10.1038/s41467-019-09065-5 (PMC6405829; doi:10.1038/s41467-019-09065-5)
Supplement: Supplementary file 2 — Peer Review File [file 41467_2019_9065_MOESM2_ESM.pdf]

Reviewers' comments:

Reviewer #1 (Remarks to the Author):

In this manuscript, the authors describe the heterologous biosynthesis and characterisation of a novel glycocin, PalA, from the thermophile Aeribacillus pallidus 8 and have shown that it most likely is glycoactive, i.e. requires a hexose covalently attached to cysteine 35 for fill activity. There are only a few examples of glycocins that have been reported so far, and this paper reports the discovery of another that can be classified as most likely belonging to the sublancin class. Furthermore, it reports the heterologous in vivo synthesis of palA in an E. coli host using its own biosynthetic gene cluster. It is therefore represents a significant advance in glycocin research. However, I feel that there is a lack of detail provided in the methods and results section, and believe more evidence needs to be provided for some of the experimental results (which appear to throw some doubt on some results) to be absolutely sure of the conclusions drawn. There is a general lack of rigor in the experimental and result reporting, which may or may not be a result of experimental design. For further improvement, I recommend that the points below be addressed by the authors before the work is published. This may, unfortunately, require extra experimental work to be carried out.

I would like to see more detail in the supplementary information about the cloning of the gene cluster. I would expect to see evidence that all the genes of the cluster are present in the pBAD plasmid by, for example, diagnostic PCR. This would require a list of primers used and a figure of a gel showing fragments of expected sizes. Subsequent experiments producing palA and other bacteriocins using strategies that involve co expression of the bacteriocins with PalS, PalT, or both in pDUET also need evidence to validate the constructs and hence the expression. Protein expression is only validated by antimicrobial activity which for the most part lack the appropriate controls. Most of the material produced using these constructs appears to be in the insoluble pellet which is extracted and refolded. Interestingly according to the supplementary information, the refolding solution contains 6M GdHCl, but no reducing agent which assumes the correct disulfides are formed in the E, coli cells. This is highly unlikely and normal protocol for the method referred to uses reducing agents. Also the peptide concentration measured at 280 using a nano drop is not reliable. The theoretical extinction coefficient for a 1 mg/L solution is 2.569. Was this taken into account? Furthermore this is a most inaccurate method for determining concentrations. A more accurate determination is done using the method of Scopes (Analytical Biochemistry 59, 277-252 (1974). Correct determination of protein concentration will have an enormous impact on the MIC.

In the supplementary information, the description of the purification of the synthesised peptides omits any mention of the soluble peptides. There are no protein gels to show the soluble vs insoluble fractions of recombinant products, and the distribution of the products in these fractions. I feel these would normally be shown, even if the proteins are produced in very low concentrations. In such circumstances, a western blot using anti-his antibodies could be used to confirm protein expression in the different systems and support the mass spectrometry results, or silver or fluorescent stains could be used for detection.

The mass spectrometry analyses in my opinion need revising as they raise many questions that need to be addressed.

Analysis of the PalA product produced by the cluster confirms that a product of the correct mass has been obtained. The identification of cysteine 35 as the site of glycosylation relies on mass spectrometry sequencing of a chymotryptic peptide. The grey shading in Supplementary figure 2 cannot be easily seen and needs to be darkened. Furthermore, when I looked at the masses that were supposedly identified as fragment ions, I could not distinguish many of them. Why was Proteome Discoverer not used to identify the PTM? Given that a Q Exactive plus was the instrument used I would

like to see an analysis that provides some sort of validation of the sequence before this work is accepted for publication.

Furthermore, if this instrument is available, the masses of the other pre-, core, and modified proteins should be measured using it rather than by the Voyager. After all, the recombinant glycocins should be soluble for antimicrobial assays to be carried out, and indeed to function in their physiological environment. The inferior mass accuracy of the latter is not good enough to confidently assess disulfide bond status and would rectify some of the uncertainties raised by the average masses obtained for the other recombinant products. Details of my concerns are as follows:

The differences between predicted and observed average masses in Figures 4-8 cast some doubt on the identification of these compounds. In Figure S4, the average mass observed for pre-PalA-His Glc of 8045.51 Da is 10 Da higher than the calculated mass. This is well outside the mass accuracy claimed for the Voyager (+/- .05 % according to their web site) , and in my opinion casts doubt that this mass is PalA. In Fig S5, (methionine is incorrectly spelt) the observed mass of pre-SunA-His, 6818 Da, compared to the calculated mass of 6800 Da is rationalised as being due to an oxidised methionine, which adds 16 Da to the mass, not the 18 Da observed, which is more likely to be water. The presence of a peak with a mass of 7133 (+ 333 Da) is described as pre-SunA-His with an unknown modification. This is more likely to be another molecule than modified pre-SunA-His (Based on the author's calculations). In Figure S 6, the numbers for the calculated masses are only correct if a 6His tag has been used, rather than the GGHHHHHHHH tag described in the methods. I could not see any mention of a change in this tag for production of hyp-1 and Hyp-2. Furthermore the observed mass is 21 Da higher than the calculated mass which most certainly does not equate to Met oxidation even accounting for the maximum error in the instrument. It is more likely to be a sodiated ion. In Fig S6C, the calculated masses are incorrect. The calculated difference between the glycosylated and non-glycosylated pre-Hyp1-6His- is 145 Da rather than the correct mass difference of 162 Da. In fact, the calculated masses appear to be wrong. I get 7912.03 for the non-glycosylated mass and 8074 .05 for the glycosylated mass, so the observed mass is unlikely to be the molecule of interest. The same pattern is seen in Figure S7 where masses attributed to expected values modified by the oxidation of methionine vary by 14 (methylation) or 15, rather than 16. Figure S8 is a puzzle. According to my calculations the theoretical mass of Hyp1-6His-Glc is 5396.94 Da which is seen as a +1 ion in Fig S8A, The mass of 6392.8 Da in Fig S8B is within error of the theoretical mass of Hyp-2-6His_Glc (6395 14). In other words, the calculated masses are incorrect and the spectra show the expected ions, even although the mass accuracy is much less than expected for this instrument (+/- 1 ppm). Yet these are described as being formylated or dimethylated.(Where is the proof?)

Is there a reason why none of this characterisation was done on the more accurate ESI instrument? To confirm helical content requires characterisation of the secondary structures of the molecule. This can be done with low volumes (`100 microlitres) of protein solution using Far UV CD and a sandwich cell. As it seems possible to obtain enough palA from the pre-his-Xa-PalA-Glc construct to carry ou such an analysi, this should be done to confirm the assumptions made about the structure and the effects of glycosylation or lack of it. I also note there is no characterisation (mass spectrometry, SDS PAGE, or evidence of activity) provided for this product.

In the section, describing the application of the PalS and PalT enzymes for the biosynthesis of other glycocins the reader is referred to Fig 2b rather than Fig 2.

In lines 200 and 202, pre-palA-His should be added to the list.

Line 210 states a His tag was added to the C-termini of core –Hyp-1 and core-Hyp-2. A 6-His tag is not specified for Hyp-1 and Hyp-2 in contrast to the tag for PalA, which is specified in the supplementary material as a GGHHHHHHHH tag. Yet the calculated masses include a 6-His tag. This is

confusing and needs to be made clear. One way of doing this would be to show the primers for each construct with the flanking amino acid sequences.

Lines 224-5. There could be other reasons for lack of activity. The most likely being miss-folding of the peptide due to the free thiol at position 35. This will lead to peptide aggregation, interaction with other peptides/proteins in the media, and non-native disulfides, all of which will abolish activity. One way to check that the molecules have the correct secondary structure is to run a far UV CD spectra as mentioned above. This has to be done before such assumptions are made.

Line 281. This categorisation was proposed in reference 8 (sup info) and should be referenced.

The MIC determination is unsatisfactory, as it shows no evidence of how the measurements were done. Was the plate read continuously during the 18 hours? If so at what time intervals. How many replicates were done? Were controls using the same concentrations of ACN and TFA in the media included? Why are there no growth curves included in the supplementary information. These concerns must be addressed before the MIC value can be accepted.

There are many typographical and grammatical errors in the text that need to be corrected before the manuscript can be accepted. I have not tabled these but the script needs careful editing.

Reviewer #2 (Remarks to the Author):

In this manuscript, Kaunietis et. al were able to characterize the biosynthesis of a new ribosomally synthesized and post-translationally modified peptide, pallidocin. Pallidocin belongs to the glycocin RiPP family. This peptide contains the post-translational modifications characteristic of this RiPP family, disulfide bonds and S-glycosylation. Different to other glycocins, pallidocin is produced by a thermophilic organism and shows antimicrobial activity against thermophilic bacteria, a property the authors recognize could find utility to prevent contamination of bioreactors operated at high temperatures. In addition to providing structural characterization of pallidocin, the authors were able to identify its biosynthetic gene cluster by expressing this peptide and its tailoring enzymes heterologously in E. coli. The substrate tolerance of the glycosyltransferase PalS was determined and using the precursor peptide palA (pallidocin precursor peptide) the authors were able to identify two additional glycocins from different organisms. The work also identified the importance glycosylation and disulfide bond formation has on the biological activity of pallidocin. This work expands the number of glycocins currently characterized emphasizing the amazing capability of microorganisms to produce natural products and it will be of broad interest to scientists. Whereas the data presented by the authors support most of their claims, in some instances I considered the need for further explanations or inclusion of some additional data to further strengthen their conclusions and statements. In order to improve the readability and strengthen the manuscript, this reviewer provides these suggestions for the authors:

1. Line 12: “modified with a” I think it reads better as “modified with an”

2. Line 17: spell out MIC

3. To me it is not clear from the intro what is the gap in knowledge the authors are trying to fill. I think it will help the readers if the authors explicitly state what is currently unknown and how their research answered some of those questions.

4. Line 31: I am not sure how this sentence fits into the overall architecture of the paragraph. The

way that is written does not convey a clear message as to how the thermophilic bacteria helps in biofuel production. Is it by producing bacteriocins thus killing competing organisms? If it is, I think it has to be clearly stated to avoid confusion by the reader. For example, other alternatives for the beneficial use of thermophilic bacteria could be because they are able to produce more biofuel metabolites, or because you can operate reactors at higher temperatures. Then after this sentence, they can mention the need for discovery of bacteriocins from thermophiles, giving a nice segway into the need of the research and as to why they decided to characterize this cluster from the many other clusters available in the databases. Such explanation will help the reader understand why they choose the current organism for their studies, an explanation that is currently not present in the manuscript.
5. From the text, it is not apparent the reason why the authors choose to pursue identification of bacteriocins in Aeribacillus pallidus. I think it will help the reader if the authors provide an explanation as to why they analyzed that genome vs other organisms.

6. Figure 1. Legend. Change "biosynthesis" for "biosynthetic"

7. Line 81. For consistency in the RiPP nomenclature, I suggest changing "pre-peptide" to "precursor peptide" See Arnison et al. 2013 Nat. Prod. Rep. 108-160.

8. Line 82. Change "SunS-family" to "Suns-like family" Change "SunT-superfamily" to SunT-like superfamily

9. Line 84. Change "DsbB disulfide" to "DsbB-like disulfide"

10. Line 87-88. I think it is important for the authors to specify that the pal operon was cloned under an Ara promoter and that the RBS of the plasmid was used. Since they are cloning the whole operon it might not be clear to the reader as to whether or not they also utilized endogenous promoters and RBS present in the pal sequence for expression in E. coli. They have an explanation on the supplementary information but I feel that information should be included in this sentence in the main text. With this explanation, the following sentence where they describe using arabinose for pal expression is better understood.

11. Line 97. When referring to the masses of the compounds I think it will help if the reader explicitly state what [M+H] species they are detecting (i.e. single charged, doubly charged etc) That way the reader will know what is the actual mass of the compound. As right now I believe all of their masses are [M+H] species but this is not clarified in the manuscript. For example, the actual predicted monoisotopic mass of the unmodified PalA core peptide is 4060.76. This is important because some of the post-translational modifications they see (disulfide bond formation) are only 2 Da in change.

12.Lines-103-107. Supplementary figure 2. Please change the orientation of the figure horizontally. Based on sequence similarity to previous glycocin precursor peptides it is very likely that indeed the site for glucose attachment is Cy25. However, the data that they refer to in the manuscript supporting that claim is not conclusive and is not sufficient to assign with certainty that the attachment of glucose occurs at Cys25. My reasoning is as follows: Whereas they indeed identified a fragment that contains a post-translational modification of +162 the individual diagnostic ions b7, b8, b9, y5, y4, y3 that will indeed confirm the site of modification to be Cy25 are either not present or their signal is less than 15%, making it hard for the reader to assess whether the signals are indeed above noise. Some of the corresponding signals are not labeled in the mass spec as well. I think labeling the b and y ions on the mass spectrum will help the reader. In addition, I believe the table they show with the predicted masses for the b ions is not accurate. The b ions up to b7 should have masses corresponding to no post-translational modification since the modification is predicted to occur Cys25. The first b ion that

will carry such modification will be b8. As such the masses in the table corresponding to b-162 and b up to b7 should be the same. This is currently not the case. Overall, while I don't disagree with the authors that indeed the modification occurs at Cys25 I think the data does not show this conclusively and instead I think the authors need to reword the manuscript to show that they were able to identify a minimal fragment carrying the +162 and that based on sequence conservation to other glycocin and glycosyltransferases it is very likely the site of attachment is Cys25.

13. Line 106 "the moiety attached to the Cys25 residue is glucose" Since all these studies where performed in E. coli how do we know the final product produced by the natural producer contains glucose as well? I think the authors need to comment on the possibility of being a different sugar since the product is not being isolated from the natural producer and as such we don't know the real identity of the final product.

14. The authors made statements describing the pH and thermal stability of pallidocin. I think it will be useful for the reader if the stability of pallidocin is compared to other glycocins.

15. line 143. The authors mention they saw an increase in mass of 234 and 228 when they treated their peptides with TCEP and IAA suggesting 4 alkylation events. I was not clear why the difference in both masses? Shouldn't they be the same increase in mass? Why there is a +6Da difference in one of the peptides? I think the authors need to comment on this. Is this based on the error of the instrument? Also, the result of this experiment suggests they were obtaining a compound with the disulfide bonds in place after coexpression in E. coli. Given the reducing environment in the cytoplasm of this organism, I think the authors should comment as to why they think they were able to get the disulfide installed if that was the case. Were these peptides obtained from the coexpression of PalS and PalT or when they expressed the whole cluster? I think that distinction might also help further understand their statements.

16. Line 161-162. I think it will help the reader if the authors could include a figure showing the size of the inhibition radius for both compounds (peptide with leader and without leader)on their agar diffusion assays. Also please include the name of the strain used to measure sensitivity. I am not familiar with the measurements the authors provide of 160 AU/mL and -320 AU/mL. I think it will help the reader if they give a brief explanation of what they mean.

17. Lines 166-190. The authors used the precursor peptide palA as a query to look for other glycocin molecules. They discuss the identification of two putative glycocins but they don't discuss the genomic context of those peptides. Are there SunS like enzymes encoded in the vicinity? I think it will help if the authors further expand to include a picture of the gene cluster and to discuss if there are coding genes for tailoring enzymes next to the genes coding for those peptides. This is important because, for instance, the ability of PalS to modify these peptides could be because either it has a high degree of sequence similarity to the native glycosyltransferase of the Hyp1/2 system, or because PalS has increase substrate tolerance and could be useful for combinatorial biosynthesis approaches. If PalS and the glycosyltransferase from Hy1 share a high degree of similarity perhaps might not be that surprising that PalS can modify Hyp1/2

18. Lines 192-225. The authors mention that glycosylation plays a crucial role in the antimicrobial activity of Hyp1 and Hyp2. However, I believe they don't mention anything about the state of the disulfide bonds present in those molecules. For palA it was shown that indeed disulfide bond formation was important for activity. I will like to know if the same is true for Hyp1 and Hyp2. Another possible explanation for their result is that the oxidation state between unmodified Hyp1/Hyp2 and unmodified

leaderless Hyp1/Hyp2 is different leading to their difference in activity. I think further clarification from the authors is needed.

19. Line 214. How do the authors know that the antimicrobial activity is due to the glycosylation or due to the presence of disulfide bonds on these molecules? I think the authors need to further clarify if when they coexpressed HypA peptides if they are able to obtain the disulfide linkages installed. See comment before

20. Line 239-245. The authors mention MIC values for pallidocin but there is no data associated with those values. This reviewer believes that having the graphs used to calculate MIC in either the main text or supplementary material will help the reader.

21. Line 252-253. I believe this statement is too strong and too bold. While the authors are able to express the whole gene cluster in E. coli, the authors failed to recognize and acknowledge another study were the genes involved in sublancin biosynthesis were expressed in E coli leading to sublancin production in E.coli, hence not being the first (ACS Chem. Biol., 2017, 12 (12), pp 2965–2969). The authors could argue that there were the first one to clone and express the whole gene of a glycocin in a heterologous host, but if we consider the final result, which is the production of a glyccocin in E coli, I believe the other study needs to be acknowledge. I also think further clarification on this statement is needed from the authors.

Overall I think with these additional suggestions the manuscript will be strengthened.

Reviewer #3 (Remarks to the Author):

What are the major claims of the paper?
In their manuscript Kaunietis et al., describe the discovery of a new ribosomally synthesized and post-translationally modified peptide (RiPP): pallidocin. Pallidocin belongs to the small class of glycocins and has been heterologously expressed in E. coli. Furthermore two other gene clusters encoding new glycocins have been identified in two Bacillus strains and have been co expressed with pallidocin enzymes to produce glycocins in vivo.

The manuscript provides interesting findings for the scientific community with some insights into the biosynthesis of glycocins. However, some improvements could be done concerning the structure and presentation of the data. It took me some time and efforts to understand the novelty of the scientific data presented in the manuscript. Maybe that could be addressed by pointing out more clearly the results in the manuscript compared to past studies. There exists, for example, a working in vitro system to produce glycocins (Oman et al., Nat Chem Biol. 2011 Feb; 7(2): 78–80.) What exactly is the advantage of the in vivo system (maybe yields, or handling)? Also, there are established in vivo expression system established for other RiPP classes auch as lantibiotics (Caetano et. al., Appl Environ Microbiol. 2011 Jul; 77(14): 5023–5026), microviridins (Weiz et al., Chem Biol. 2011 Nov 23;18(11):1413-21), and cyanobactins (Schmidt et al., Proc Natl Acad Sci U S A. 2005 May 17;102(20):7315-20).How unique is the expression system presented here? What new things can we learn regarding the biosynthesis of glycocins, especially the promiscuity of PalS compared to SunS? It would be interesting to see how common sublancin type glycocins are in the bacterial kingdom?

Furthermore, I am not sure how to interpret the results of the co-expression of the core_hip genes with palS (page 11). According to the authors both peptides showed antibacterial activity even when not glycosylated. The authors suggested that the glycosylation rate of the peptides were too low for detection. As far as I understand MS is very sensitive and glycosylation patterns relatively easy to

detect. Could there be another explanation?

Minor points:
The scientific community calls these peptides RiPPs. Is there any reason the authors don't use this term?

It would be interesting to know more details about the specific activity of pallidocins against thermophilic bacteria. Any reason how the mode of action is there?

P5 l94/94: It seems unusual to me that the yield is enough for activity screening and structure elucidation but not for quantification at 280 nm.

The authors talk always about proposed structures. How sure are you about the exact structure of the compound?

Reviewer #1:

In this manuscript, the authors describe the heterologous biosynthesis and characterisation of a novel glycocin, PalA, from the thermophile Aeribacillus pallidus 8 and have shown that it most likely is glycoactive, i.e. requires a hexose covalently attached to cysteine 35 for fill activity. There are only a few examples of glycocins that have been reported so far, and this paper reports the discovery of another that can be classified as most likely belonging to the sublancin class. Furthermore, it reports the heterologous in vivo synthesis of palA in an E. coli host using its own biosynthetic gene cluster. It is therefore represents a significant advance in glycocin research.

However, I feel that there is a lack of detail provided in the methods and results section, and believe more evidence needs to be provided for some of the experimental results (which appear to throw some doubt on some results) to be absolutely sure of the conclusions drawn. There is a general lack of rigor in the experimental and result reporting, which may or may not be a result of experimental design. For further improvement, I recommend that the points below be addressed by the authors before the work is published. This may, unfortunately, require extra experimental work to be carried out.

1. I would like to see more detail in the supplementary information about the cloning of the gene cluster. I would expect to see evidence that all the genes of the cluster are present in the pBAD plasmid by, for example, diagnostic PCR. This would require a list of primers used and a figure of a gel showing fragments of expected sizes. Subsequent experiments producing palA and other bacteriocins using strategies that involve co expression of the bacteriocins with PalS, PalT, or both in pDUET also need evidence to validate the constructs and hence the expression.

   Answer to reviewer

   We have been using a conventional strategy for the cloning part – a colony PCR and full DNA sequencing methods to check the constructs. DNA sequencing of the constructs is reliable and a common method for validation of constructs and the subsequent expression. We think that the latter method is more accurate than a diagnostic PCR. We updated the Methods section in the Supplementary Information, and included more detailed information about the cloning. We indicated which primers were used for

colony PCR and DNA sequencing. Also all the primers are now provided in Supplementary Table 5.

2. Protein expression is only validated by antimicrobial activity which for the most part lack the appropriate controls.

Answer to reviewer

Expression of the precursor peptide was demonstrated by purification of His-tagged peptides using highly selective Ni^{2+} affinity chromatography (IMAC) and RP-HPLC chromatography methods, and subsequently identification of the purified peptide by MALDI-TOF mass spectrometry.

Regarding PalS and PalT: expression of these proteins was confirmed by observation of modifications in the purified His-tagged precursor peptide. In case of expression of PalS, the precursor was glycosylated – the mass of the peptide was increased accordingly. In case of PalT expression, the precursor lost its leader sequence – the mass of the peptide was decreased accordingly. For these experiments the appropriate control is the expression of only precursor peptide; other kinds of controls seem meaningless in this case. Analyses by MALDI-TOF of these purified unmodified (control) and modified precursors were provided before and now can be found in the new updated Supplementary Figures 7-9, 11-16.

Regarding the activity controls: they were provided in Table 1, where it is indicated that nonmodified glycocin precursors show no activity. Controls of expression or activity using noninduced E. coli or E. coli without a plasmid are meaningless. No peptide can be produced and purified by Ni^{2+} affinity chromatography (IMAC) and RP-HPLC chromatography methods and subsequently be identified by MALDI-TOF mass spectrometry, or evaluated for activity.

For more complete data and results we now include new Figure 3 of activity assays of unmodified (control) and modified peptides.

3. Most of the material produced using these constructs appears to be in the insoluble pellet which is extracted and refolded. Interestingly according to the supplementary information, the refolding solution contains 6M GdHCl, but no reducing agent which assumes the correct disulfides are formed in the E. coli cells. This is highly unlikely and normal protocol for the method referred to uses reducing agents.

Answer to reviewer

We have followed similar methods and experimental design as described in a study on sublancin 168 (Nature Chemical Biology, 7(2): 78-80, 2011). This group also did not use any reducing agents for purification of sublancin precursor peptide from the insoluble fraction. They did not provide experimental data if disulfide bonds were formed in the peptide after extraction from E. coli. After the purification and in vitro glycosylation (reduced conditions) of the peptide they did a chemical oxidative folding in vitro.
When synthesized peptides are located in the cytoplasm the formation of disulfide bonds is unlikely because of the reducing environment. During the purification process the oxidative folding in the peptides could occur spontaneously by air oxidation. This explains why our purified glycosylated peptides are oxidatively folded and active. Only when the precursor peptide PalA and peptidase/ABC transporter PalT are co-expressed, the synthesized peptide should be transported to the periplasm by PalT, where the oxidative folding could take place because of the oxidative environment.
The Gua-HCl dissolved peptides of the insoluble fraction, which we assume were formed not by disulfide bonds between the peptides, but most probably by hydrophobic interactions between the peptides.

4. Also the peptide concentration measured at 280 using a nano drop is not reliable. The theoretical extinction coefficient for a 1 mg/L solution is 2.569. Was this taken into account? Furthermore this is a most inaccurate method for determining concentrations. A more accurate determination is done using the method of Scopes (Analytical Biochemistry 59, 277-252 (1974). Correct determination of protein concentration will have an enormous impact on the MIC.

Answer to reviewer

We have measured the concentrations of peptides using the molar absorptivity (extinction coefficient) at 280 nm, which was calculated directly from the sequence of the peptide. This method offers high specificity, as it arises strictly from Trp and Tyr residues and to a small extent from disulfide bonds (Protein Science, 22(6): 851-858, 2013). 5 Tyr, 1 Trp and 2 disulfide bonds are present in mature pallidocin, which let us to calculate specific molar absorptivity (extinction coefficient) of the peptide at 280 nm = 13200 M^{-1} cm^{-1} (0.1% solution (1 g/L) = 3.25). This approach let us to avoid measurement errors, which might be caused by DNA, RNA or other contaminations in the sample.

In response to your comments we also did measurements of absorption at 205 nm (calculated specific molar absorptivity (extinction coefficient) at 205 nm = 171330 M^{-1} cm^{-1}). The result indicated the same quantity of the peptide as was determined after measurements at 280 nm.

Changes in supplementary information

The quantity of purified peptide was measured by NanoPhotometer N60 (Implen). The molar absorptivity (extinction coefficient) was calculated (at 280 nm – 13200 M^{-1} cm^{-1}, at 205 nm – 171330 M^{-1} cm^{-1}) based on the peptide sequence. Calculations were performed by a web tool at http://spin.niddk.nih.gov/clore[1]. Determined quantity of the peptide by measurements either at 280 or 205 nm was the same.

5. In the supplementary information, the description of the purification of the synthesized peptides omits any mention of the soluble peptides. There are no protein gels to show the soluble vs insoluble fractions of recombinant products, and the distribution of the products in these fractions. I feel these would normally be shown, even if the proteins are produced in very low concentrations. In such circumstances, a western blot using anti-his antibodies could be used to confirm protein expression in the different systems and support the mass spectrometry results, or silver or fluorescent stains could be used for detection.

Answer to reviewer

We have followed very similar methods and experimental design as described in the analysis of sublancin 168 where they extracted synthesized sublancin precursor peptide from the insoluble fraction as well (Nature Chemical Biology, 7(2): 78-80, 2011). Moreover, our aim was to purify synthesized peptides for the subsequent characterization by mass spectrometry and antibacterial activity assessment. Results of mass spectrometry analysis of the purified peptides (His-tagged) are clear and require no support by other methods, like SDS-PAGE. Thus, the distribution of synthesized peptides in soluble and insoluble fractions would not give us essential information about them. The peptides are present in the soluble and insoluble fractions. In the end, after the purification by highly selective Ni^{2+} affinity chromatography and RP-HPLC, most of the peptides were extracted from the insoluble fraction. We did not see any reason to purify and analyze peptides from the soluble fractions further, because of very low yields.

The mass spectrometry analyses in my opinion need revising as they raise many questions that need to be addressed.
Analysis of the PalA product produced by the cluster confirms that a product of the correct mass has been obtained. The identification of cysteine 35 as the site of glycosylation relies on mass spectrometry sequencing of a chymotryptic peptide.

6. The grey shading in Supplementary figure 2 cannot be easily seen and needs to be darkened.

   Answer to reviewer

   We have included new Supplementary Figure 3 to represent these data.

7. Furthermore, when I looked at the masses that were supposedly identified as fragment ions, I could not distinguish many of them. Why was Proteome Discoverer not used to identify the PTM? Given that a Q Exactive plus was the instrument used I would like to see an analysis that provides some sort of validation of the sequence before this work is accepted for publication.

   Answer to reviewer

   We redesigned the figure that represents MS/MS results and highlighted the fragment ions, which are essential for the conclusion that Cys25 has a posttranslational modification. To supplement and consolidate the results confirming that Cys25 has a +162,05 Da posttranslational modification, we repeated the analysis (trial 2) by MS/MS. We have included a new Supplementary Figure 3 to represent these data.

8. Furthermore, if this instrument is available, the masses of the other pre-, core, and modified proteins should be measured using it rather than by the Voyager. The inferior mass accuracy of the latter is not good enough to confidently assess disulfide bond status and would rectify some of the uncertainties raised by the average masses obtained for the other recombinant products. Details of my concerns are as follows:

   a) The differences between predicted and observed average masses in Figures 4-8 cast some doubt on the identification of these compounds. In Figure S4, the average mass observed for pre-PalA-His-Glc of 8045.51 Da is 10 Da higher than the calculated mass. This is well outside the mass accuracy claimed for the Voyager (+/- .05 % according to their web site), and in my opinion casts doubt that this mass is PalA.

   b) In Fig S5, (methionine is incorrectly spelt) the observed mass of pre-SunA-His, 6818 Da, compared to the calculated mass of 6800 Da is rationalised as being due to an

oxidised methionine, which adds 16 Da to the mass, not the 18 Da observed, which is more likely to be water. The presence of a peak with a mass of 7133 (+ 333 Da) is described as pre-SunA-His with an unknown modification. This is more likely to be another molecule than modified pre-SunA-His (Based on the author's calculations).

c) In Figure S 6, the numbers for the calculated masses are only correct if a 6His tag has been used, rather than the GGHHHHHHH tag described in the methods. I could not see any mention of a change in this tag for production of hyp-1 and Hyp-2. Furthermore the observed mass is 21 Da higher than the calculated mass which most certainly does not equate to Met oxidation even accounting for the maximum error in the instrument. It is more likely to be a sodiated ion. In Fig S6C, the calculated masses are incorrect. The calculated difference between the glycosylated and non-glycosylated pre-Hyp1-6His- is 145 Da rather than the correct mass difference of 162 Da. In fact, the calculated masses appear to be wrong. I get 7912.03 for the non-glycosylated mass and 8074 .05 for the glycosylated mass, so the observed mass is unlikely to be the molecule of interest.

d) The same pattern is seen in Figure S7 where masses attributed to expected values modified by the oxidation of methionine vary by 14 (methylation) or 15, rather than 16.

Is there a reason why none of this characterization was done on the more accurate ESI instrument?

Answers to reviewer (questions 8a-d)

The primary aim of this analysis was not identification of disulfide bonds, but identification of posttranslational modifications (glucosylation) and leader cleavage performed by PalS and PalT. In this case the mass accuracy of the instrument Voyager is certainly suitable. The differences between calculated masses and observed masses of the purified peptides do not contradict our conclusion that PalS is glycosyltransferase that links the glucose to glycocin precursors.

Mass differences might be caused by adducts (methionine oxidation – 16 Da, methylation – 14 Da, water – 18 Da, Na^+ – 23 Da) in the peptide. Moreover, there might be mass variations because of inaccuracy of the instrument. Because all of that it is difficult to precisely determine the mass of the peptides. **To leave no doubts about the masses of the peptides and their modifications, we have now synthesized the peptides**

again, and in addition to MALDI-TOF MS analysis we performed the LC-ESI-MS analysis which gives more accuracy. New data are represented in new Supplementary Figures 7, 8, 11-16. In addition, to aid theoretical mass calculations of the His-tagged peptides and to avoid confusions, we included new Supplementary Figure 6 with peptide sequences and calculated theoretical masses.

We agree with the reviewer that our represented results of mass spectrometry (MALDI-TOF MS) were not suitable for identification of disulfide bonds in the peptides. For this reason the presence of disulfide bonds in the pre-PalA-His-Glc and PalA-His-Glc were identified by treatment with TCEP and IAA, and subsequent analysis by mass spectrometry. These results were already represented in section "*The role of disulfide bonds on antibacterial activity of the posttranslationally modified pallidocin precursor*". Moreover, we did additional experiments: we treated Hyp1 and Hyp2 peptides with TCEP and IAA to determine if disulfide bonds are present in these peptides. These new results are included in the section mentioned above and new updated Supplementary Tables 2-4.

The His7-tag (GGHHHHHHH) was used only for PalA peptide.

Precursor peptides: pre-SunA-His, pre-GccF, pre-EnfA4-9, pre-Hyp1-His and pre-Hyp2-His, had His6-tag (HHHHHH).

Changes in Supplementary Information

The His7-tag (GGHHHHHHH) was used only for the PalA peptide.

Precursor peptides: pre-SunA-His, pre-GccF, pre-EnfA4-9, pre-Hyp1-His and pre-Hyp2-His, had His6-tag (HHHHHH).

e) Figure S8 is a puzzle. According to my calculations the theoretical mass of Hyp1-6His-Glc is 5396.94 Da which is seen as a +1 ion in Fig S8A, The mass of 6392.8 Da in Fig S8B is within error of the theoretical mass of Hyp-2-6His_Glc (6395 14). In other words, the calculated masses are incorrect and the spectra show the expected ions, even although the mass accuracy is much less than expected for this instrument (+/-1 ppm). Yet these are described as being formylated or dimethylated.(Where is the proof?)

Answer to reviewer

Our theoretical mass calculations included Met1 in the sequence. We did not notice that both core peptides: Hyp1-His-Glc and Hyp2-His-Glc, have lost the Met1 (-131,2 Da). As the reviewer mentioned above, in fact the observed masses match the masses of glycosylated (+162,05 Da) core peptides with two disulfide bonds (-4 Da).

Changes in Supplementary Information

Description of Supplementary Figure 17. Calculated mass of Hyp1-His core peptide with two disulfide bonds – 5366,13 Da; glycosylated Hyp1-His-Glc core peptide with two disulfide bonds – 5528,18 Da; glycosylated Hyp1-His-Glc core peptide with two disulfide bonds and with cleaved Met1 (-131,2 Da) – 5396,98 Da. (a) Observed mass $[M+H]^+$ – 5397,3 Da represents Hyp1-His-Glc core peptide with two disulfide bonds, with cleaved Met1 and within an error (+0,32 Da).

Calculated mass of Hyp2-His core peptide with two disulfide bonds – 6365,08 Da; glycosylated Hyp2-His-Glc core peptide with two disulfide bonds – 6527,13 Da; glycosylated Hyp2-His-Glc core peptide with two disulfide bonds and with cleaved Met1 (-131,2 Da) – 6395,93. (b) Observed mass $[M+H]^+$ – 6395,82 Da which represents Hyp2-His-Glc core peptide with two disulfide bonds, with cleaved Met1 and within an error (-0,11 Da).

9) To confirm helical content requires characterisation of the secondary structures of the molecule. This can be done with low volumes (`100 microlitres) of protein solution using Far UV CD and a sandwich cell. As it seems possible to obtain enough palA from the pre-his-Xa-PalA-Glc construct to carry out such an analysis, this should be done to confirm the assumptions made about the structure and the effects of glycosylation or lack of it. I also note there is no characterisation (mass spectrometry, SDS PAGE, or evidence of activity) provided for this product.

Answer to reviewer

The characterization (mass spectrometry and activity assay) of pre-His-Xa-PalA-Glc and PalA-Glc, which was derived after leader cleavage by Factor Xa peptidase, are provided in new Supplementary Figure 9, 10. We also did CD spectrometry and included the results in new Supplementary Figure 5.

Changes in Results section

Based on the analysis by the secondary structure prediction tool PSIPRED[2] pallidocin has two α-helices (Figure 2). Far-UV CD spectra analysis of mature pallidocin (Supplementary

Figure 5) revealed that the peptide contains substantial amounts of helical structure, judging from the pattern of the spectra from 193 to 240 nm. Similar spectra patterns were observed for sublancin[3–5] and glycocin F[6], also. Estimate of the secondary structure content, made by the method of Raussens et al.[7], predicted predominantly helical structure, with an estimate of 47% helix. The peptide was also estimated to contain 13% β-turn and 11% β-sheet structure.

10) In the section, describing the application of the PalS and PalT enzymes for the biosynthesis of other glycocins the reader is referred to Fig 2b rather than Fig 2.

Corrected.

11) In lines 200 and 202, pre-palA-His should be added to the list.

Corrected.

12) Line 210 states a His tag was added to the C-termini of core –Hyp-1 and core-Hyp-2. A 6-His tag is not specified for Hyp-1 and Hyp-2 in contrast to the tag for PalA, which is specified in the supplementary material as a GGHHHHHHH tag. Yet the calculated masses include a 6-His tag. This is confusing and needs to be made clear. One way of doing this would be to show the primers for each construct with the flanking amino acid sequences.

Corrected.

13) Lines 224-5. There could be other reasons for lack of activity. The most likely being miss-folding of the peptide due to the free thiol at position 35. This will lead to peptide aggregation, interaction with other peptides/proteins in the media, and non-native disulfides, all of which will abolish activity. One way to check that the molecules have the correct secondary structure is to run a far UV CD spectra as mentioned above. This has to be done before such assumptions are made.

Answer to reviewer

In contrast to previous work on sublancin[1], a new published study showed that non glycosylated and oxidatively folded core peptide of sublancin has the same topology of disulfide bonds as the native mature sublancin. In fact, the previous assumptions that the free thiol of unmodified Cys disrupts the formation of the correct disulfide bridges by thiol-disulfide exchange, and the blocked Cys residue can aid to form correct disulfide bonds between four free Cys residues[1], were incorrect. For this reason we assume that nonglycosylated PalA, Hyp1 and Hyp2 core peptides have correct disulfide bonds, also.

We cannot do far UV CD spectrometry analysis of Hyp1-His(-Glc) or Hyp2-His(-Glc) core peptides because glycosylated and nonglycosylated peptides are not well enough separated by our RP-HPLC system. The complex mixture of glycosylated and nonglycosylated peptides in the sample may influence the reliability of far UV CD spectrometry analysis results.

14) Line 281. This categorisation was proposed in reference 8 (sup info) and should be referenced.

Corrected.

15) The MIC determination is unsatisfactory, as it shows no evidence of how the measurements were done. Was the plate read continuously during the 18 hours? If so at what time intervals. How many replicates were done? Were controls using the same concentrations of ACN and TFA in the media included? Why are there no growth curves included in the supplementary information. These concerns must be addressed before the MIC value can be accepted.

Answer to reviewer

The bacterial growth was monitored at 55°C, and this temperature is not applicable in automatic plate readers. It is not possible to use automatic plate reader and continuously measure the absorbance in the plate at high temperatures. Moreover, the measurements should be performed without a plate lid, because of water condensation on it at high temperature; it would result in nonsterile conditions. Negative controls contained the same concentrations of ACN, TFA and pallidocin as well as nondiluted samples. Positive controls did not include ACN and TFA in the medium.

Now we have repeated positive controls with ACN and TFA included in the medium. It did not influence the results.

We have provided a new Supplementary Figure 18 with final evaluations of the growth (made by OD measurements) in the plates.

Changes in Supplementary Information

Positive controls – 150 µL mixture of NB medium with the sensitive strain (5×10^5 CFU/mL), and negative controls – 150 µL mixture of NB medium with 5 µL of pallidocin solution (1 ng/µL in 50 % ACN and 0.1 % TFA), were prepared and dispersed in the same 96 well plates. The plate with a lid was placed in a plastic box (12 cm x 20 cm x 6 cm) with a wet paper towel to keep high humidity and prevent medium evaporation at high

temperature. The plate was incubated in a thermoshaker for 18 hours at 55˚C and 250 RPM. After incubation the growth of bacteria was evaluated by a plate reader and visually. The analyses were performed in triplicate.

It should be noted that because of the high mutation rate and emergence of resistant mutants in some wells in the plate, the calculation of average MIC from three replicates are prone to variation. Final MIC was determined by the lowest amount of pallidocin required to inhibit cell growth in a well. Because bacteria were grown at 55°C, it was not possible to use a plate reader at this condition as the instrument is not suited for measurements at high temperatures.

16) There are many typographical and grammatical errors in the text that need to be corrected before the manuscript can be accepted. I have not tabled these but the script needs careful editing.

Answer to reviewer

We have corrected these in the revised manuscript.

Reviewer #2 (Remarks to the Author):

In this manuscript, Kaunietis et. al were able to characterize the biosynthesis of a new ribosomally synthesized and post-translationally modified peptide, pallidocin. Pallidocin belongs to the glycocin RiPP family. This peptide contains the post-translational modifications characteristic of this RiPP family, disulfide bonds and S-glycosylation. Different to other glycocins, pallidocin is produced by a thermophilic organism and shows antimicrobial activity against thermophilic bacteria, a property the authors recognize could find utility to prevent contamination of bioreactors operated at high temperatures. In addition to providing structural characterization of pallidocin, the authors were able to identify its biosynthetic gene cluster by expressing this peptide and its tailoring enzymes heterologously in E. coli. The substrate tolerance of the glycosyltransferase PalS was determined and using the precursor peptide palA (pallidocin precursor peptide) the authors were able to identify two additional glycocins from different organisms. The work also identified the importance glycosylation and disulfide bond formation has on the biological activity of pallidocin. This work expands the number of glycocins currently characterized emphasizing the amazing capability of microorganisms to produce natural products and it will be of broad interest to scientists. Whereas the data presented by the authors support

most of their claims, in some instances I considered the need for further explanations or inclusion of some additional data to further strengthen their conclusions and statements. In order to improve the readability and strengthen the manuscript, this reviewer provides these suggestions for the authors:

17) Line 12: “modified with a” I think it reads better as “modified with an”

Corrected.

18) Line 17: spell out MIC

Corrected.

19) To me it is not clear from the intro what is the gap in knowledge the authors are trying to fill. I think it will help the readers if the authors explicitly state what is currently unknown and how their research answered some of those questions.

Changes in Introduction section

To date only two bacteriocins, *i.e.* geobacillin I and geobacillin II, produced by the thermophilic bacteria *Geobacillus thermodenitrificans* NG80-2, are well characterized[8–10]. Other bacteriocin-like antibacterial compounds from thermophilic microorganisms have been described at much less detail[11–17]. These reasons prompted us to find and to study new bacteriocins of this group of microorganisms. Thus, we have chosen the thermophilic *Aeribacillus pallidus* 8 strain that was previously isolated from soil above oil wells in Lithuania[18]. Previous studies have shown that this strain secretes an antibacterial compound that is active against other thermophilic bacteria. Unfortunately purification of this compound and identification of its amino acid sequence were not successful[18,19]. In this study we have identified genes in the genome of *A. pallidus* 8 that encode a biosynthetic machinery of a novel bacteriocin – pallidocin, which belongs to a small class of glycocins. This is the first case where a glycocin is produced by a thermophilic bacterium. Here, for the first time we present the functional expression of the whole biosynthetic gene cluster of a glycocin in Gram-negative *Escherichia coli*, which facilitates further engineering and mechanistic studies.

Previously, full maturation of recombinant glycocins was only reported *in vitro* for thurandacin and sublancin. Glycosylation and leader cleavage was performed enzymatically, followed by chemical oxidative folding[20,21]. The *in vitro* experiments limit the yield of the end product, are time consuming and expensive. Recently, a system was developed for the heterologous expression of sublancin in *E. coli* SHuffle T7 Express cells

that *in vivo* installs the glycosylation and oxidative folding following a single *in vitro* step of proteolytic leader cleavage[4]. SHuffle T7 Express strain expresses the disulfide bond isomerase DsbC, aiding oxidative folding of proteins in citoplasm[22]. Here we demonstrate a different *in vivo* heterologous expression system to produce completely mature novel glycocins in *E. coli* BL21(DE3), evading the *in vitro* chemical and enzymatical steps.

20) Line 31: I am not sure how this sentence fits into the overall architecture of the paragraph. The way that is written does not convey a clear message as to how the thermophilic bacteria helps in biofuel production. Is it by producing bacteriocins thus killing competing organisms? If it is, I think it has to be clearly stated to avoid confusion by the reader. For example, other alternatives for the beneficial use of thermophilic bacteria could be because they are able to produce more biofuel metabolites, or because you can operate reactors at higher temperatures. Then after this sentence, they can mention the need for discovery of bacteriocins from thermophiles, giving a nice segway into the need of the research and as to why they decided to characterize this cluster from the many other clusters available in the databases. Such explanation will help the reader understand why they choose the current organism for their studies, an explanation that is currently not present in the manuscript.

Changes in Introduction section

Thermophilic bacteria have shown a great potential in biofuel production because of their higher metabolic rate and enzyme stability at elevated temperatures. Moreover, growth at high temperature facilitates recovery of volatile products, like ethanol[23], and reduces requirement for cooling. Thermophilic fermentations are less prone to contaminations by mesophiles, although there are still risks that bioreactors will be contaminated by other thermophiles[24,25]. In addition, contamination by thermophiles is also a problem in production of dairy products[26]. This shows the need of discovery of new natural compounds which have activity against thermophilic bacteria.

To date only two bacteriocins: geobacillin I and geobacillin II, produced by the thermophilic bacteria *Geobacillus thermodenitrificans* NG80-2, are well characterized[8–10]. Other bacteriocin-like antibacterial compounds from thermophilic microorganisms have been described at much less detail[11–17].

21) From the text, it is not apparent the reason why the authors choose to pursue identification of bacteriocins in Aeribacillus pallidus. I think it will help the reader if the authors provide an explanation as to why they analyzed that genome vs other organisms.

Changes in Introduction section

See the answer to question 19.

22) Figure 1. Legend. Change "biosynthesis" for "biosynthetic"

Corrected.

23) Line 81. For consistency in the RiPP nomenclature, I suggest changing "pre-peptide" to "precursor peptide" See Arnison et al. 2013 Nat. Prod. Rep. 108-160.

Corrected.

24) Line 82. Change "SunS-family" to "Suns-like family" Change "SunT-superfamily" to SunT-like superfamily

Corrected.

25) Line 84. Change "DsbB disulfide" to "DsbB-like disulfide"

Corrected.

26) Line 87-88. I think it is important for the authors to specify that the pal operon was cloned under an Ara promoter and that the RBS of the plasmid was used. Since they are cloning the whole operon it might not be clear to the reader as to whether or not they also utilized endogenous promoters and RBS present in the pal sequence for expression in E. coli. They have an explanation on the supplementary information but I feel that information should be included in this sentence in the main text. With this explanation, the following sentence where they describe using arabinose for pal expression is better understood.

Corrected.

27) Line 97. When referring to the masses of the compounds I think it will help if the reader explicitly state what [M+H] species they are detecting (i.e. single charged, doubly charged etc) That way the reader will know what is the actual mass of the compound. As right now I believe all of their masses are [M+H] species but this is not clarified in the manuscript. For example, the actual predicted monoisotopic mass of the unmodified PalA core peptide is 4060.76. This is important because some of the post-translational modifications they see (disulfide bond formation) are only 2 Da in change.

Corrected.

28) Lines-103-107. Supplementary figure 2. Please change the orientation of the figure horizontally. Based on sequence similarity to previous glycocin precursor peptides it is very likely that indeed the site for glucose attachment is Cy25. However, the data that they refer to in the manuscript supporting that claim is not conclusive and is not sufficient to assign with certainty that the attachment of glucose occurs at Cys25. My reasoning is as follows: Whereas they indeed identified a fragment that contains a post-translational modification of +162 the individual diagnostic ions b7, b8, b9, y5, y4, y3 that will indeed confirm the site of modification to be Cy25 are either not present or their signal is less than 15%, making it hard for the reader to assess whether the signals are indeed above noise. Some of the corresponding signals are not labeled in the mass spec as well. I think labeling the b and y ions on the mass spectrum will help the reader. In addition, I believe the table they show with the predicted masses for the b ions is not accurate. The b ions up to b7 should have masses corresponding to no post-translational modification since the modification is predicted to occur Cys25. The first b ion that will carry such modification will be b8. As such the masses in the table corresponding to b-162 and b up to b7 should be the same. This is currently not the case. Overall, while I don't disagree with the authors that indeed the modification occurs at Cys25 I think the data does not show this conclusively and instead I think the authors need to reword the manuscript to show that they were able to identify a minimal fragment carrying the +162 and that based on sequence conservation to other glycocin and glycosyltransferases it is very likely the site of attachment is Cys25.

Answer to reviewer

See the answer to question 7.

29) Line 106 "the moiety attached to the Cys25 residue is glucose" Since all these studies where performed in E. coli how do we know the final product produced by the natural producer contains glucose as well? I think the authors need to comment on the possibility of being a different sugar since the product is not being isolated from the natural producer and as such we don't know the real identity of the final product.

Changes in Discussion section

In vitro studies on glycosylation of sublancin precursor have demonstrated that S-glycosyltransferase has a relaxed substrate specificity. It is able to attach other sugars: xylose, mannose, N-acetylglucosamine or galactose, as well. The native glycopeptide

sublancin purified from *B. subtilis* contains glucose[21]. We do not know which sugar would be present in native pallidocin if the peptide was derived from *A. pallidus* 8. We can assume that native pallidocin has an S-linked glucose as well, as this sugar was found in recombinant pallidocin produced by *E. coli*.

30) The authors made statements describing the pH and thermal stability of pallidocin. I think it will be useful for the reader if the stability of pallidocin is compared to other glycocins.

Changes in Discussion section

To our knowledge only sublancin and enterocin F4-9 have been properly characterized for their stability. The stability of sublancin is decreased by 50% after 30 min incubation at 70°C temperature. Sublancin is not very stable at acidic conditions, after incubation at pH 2 and 3 for 30 min it retains only 20% and 40% of its activity, respectively[3], while enterocin F4-9 after incubation at 80°C for 15 min retains its full activity only at pH values from 2 to 8. After incubation at 100°C for 15 min, enterocin F4-9 retains its full activity only at pH 4. Its activity is completely lost after incubation at 121°C as well as at pH 10[27]. Comparing to sublancin and enterocin F4-9, pallidocin is much more stable at high temperatures. Its activity decreases 50% only after 15 min incubation at 121 °C and is completely stable at 90°C for 3 h. In contrast to sublancin and enterocin F4-9, pallidocin retains its full activity at acidic and basic conditions (pH 2-10).

31) Line 143. The authors mention they saw an increase in mass of 234 and 228 when they treated their peptides with TCEP and IAA suggesting 4 alkylation events. I was not clear why the difference in both masses? Shouldn't they be the same increase in mass? Why there is a +6Da difference in one of the peptides? I think the authors need to comment on this. Is this based on the error of the instrument? Also, the result of this experiment suggests they were obtaining a compound with the disulfide bonds in place after coexpression in E. coli. Given the reducing environment in the cytoplasm of this organism, I think the authors should comment as to why they think they were able to get the disulfide installed if that was the case. Were these peptides obtained from the coexpression of PalS and PalT or when they expressed the whole cluster? I think that distinction might also help further understand their statements.

Changes in Results section

These peptides were derived after co-expression of *palA-his* with *palS* or *palST*.

Expected mass increment for one alkylated Cys is 57 Da, for four alkylated Cys – 228 Da. After all, despite the observed mass difference (6 Da), which is outside the accuracy claimed for the instrument (4 Da), it is obvious that the disulfide bonds were reduced and all free Cys residues were alkylated.

Changes in Discussion section

Oxidative folding of the peptide in the cytoplasm is unlikely. The formation of structural disulfide bonds in *E. coli* appears to be strictly segregated according to subcellular compartmentalization[28]. Because the reducing environment is necessary for enzymatic activity of glycosyltransferases[21] most probably PalS glycosylates peptides in the cytoplasm. When the whole gene cluster *pal* is expressed the synthesized glycosylated precursor peptide should be transported to the periplasm by PalT, where the oxidative folding could take place. After the precursor peptide co-expression with PalS these bonds could be formed spontaneously by air oxidation[29,30] during peptide extraction and the purification process. In case of precursor peptide co-expression with PalST disulfide bonds in the glycosylated core peptide could be formed in the periplasm or spontaneously by air oxidation[29,30] during the peptide extraction and purification process.

32) Line 161-162. I think it will help the reader if the authors could include a figure showing the size of the inhibition radius for both compounds (peptide with leader and without leader)on their agar diffusion assays. Also please include the name of the strain used to measure sensitivity. I am not familiar with the measurements the authors provide of 160 AU/mL and -320 AU/mL. I think it will help the reader if they give a brief explanation of what they mean.

Answer to reviewer

Previous estimates of the activities were not very accurate. Now we included new Supplementary Figure 9, 10 of activity assessment assay that shows a dilution factors which still give antimicrobial activity. The measurements of inhibition zones are not accurate method for evaluation and comparison of bacteriocin antimicrobial activities.

Changes in Results section

Activities of the glycosylated precursor peptide with leader and mature pallidocin were compared by the agar well diffusion assay using *P. genomospecies* 1 NUB36187 as a sensitive strain. The assay shows the highest serial two-fold dilution of bacteriocin

sample, which still displays antibacterial activity. Results show that pre-His-Xa-PalA-Glc had approximately 500 times lower activity than and the mature pallidocin.

33) Lines 166-190. The authors used the precursor peptide palA as a query to look for other glycocin molecules. They discuss the identification of two putative glycocins but they don’t discuss the genomic context of those peptides. Are there SunS like enzymes encoded in the vicinity? I think it will help if the authors further expand to include a picture of the gene cluster and to discuss if there are coding genes for tailoring enzymes next to the genes coding for those peptides. This is important because, for instance, the ability of PalS to modify these peptides could be because either it has a high degree of sequence similarity to the native glycosyltransferase of the Hyp1/2 system, or because PalS has increase substrate tolerance and could be useful for combinatorial biosynthesis approaches. If PalS and the glycosyltransferase from Hy1 share a high degree of similarity perhaps might not be that surprising that PalS can modify Hyp1/2.

Changes in Results section

Genome analysis of *Bacillus megaterium* BHG1.1 and *Bacillus* sp. JCM 19047 by the BAGEL4 tool did not find any gene clusters related to bacteriocin biosynthesis. However, BLASTp analysis of genomic context of *hyp1* revealed a gene cluster coding for a putative glycocin biosynthetic machinery (Fig. 4). Genes in the cluster alongside the Hyp1 precursor gene encodes for: Hyp1S protein with up to 50% sequence similarity to SunS-like family peptide S-glycosyltransferases; Hyp1T protein with up to 68% sequence similarity to SunT-like superfamily peptidase domain-containing ABC transporters; Trx protein with up to 69% sequence similarity to thioredoxin-like superfamily proteins and DsbB protein with up to 74% sequence similarity to DsbB-like superfamily disulfide bond formation proteins B. Meanwhile, BLASTp analysis of genomic context of *hyp2* revealed a gene cluster coding for a putative glycocin biosynthetic machinery (Fig. 4). Genes in the cluster alongside the Hyp2 precursor gene encode the Hyp2S protein with up to 42% sequence similarity to SunS-like family peptide S-glycosyltransferases; Hyp2T protein with up to 40% sequence similarity to SunT-like superfamily peptidase domain-containing ABC transporters and the Trx protein with up to 43% sequence similarity to thioredoxin-like superfamily proteins. Two putative novel glycocin precursors *i.e.* Hyp1 and Hyp2 were investigated and examined further for possible posttranslational modifications by the biosynthetic machinery of pallidocin.

The 42%, 50% and 53% sequence similarities of Hyp1S, Hyp2S and SunS, respectively, to the PalS S-glycosyltransferase show quite surprisingly that PalS modifies heterologous substrates.

Description of Figure 4. The predicted biosynthetic gene clusters of glycocins Hyp1 and Hyp2. Hyp1 biosynthetic gene cluster is encoded in the genome of *Bacillus megaterium* BHG1.1, is 5013 bp in length and encodes for proteins: Hyp1, Hyp1S, Hyp1T, Trx, DsbB and Hyp1U. The Hyp2 biosynthetic gene cluster is encoded in the genome of *Bacillus* sp. JCM 19047, is 3932 bp in length and encodes for proteins: Hyp2, Hyp2T, Trx and Hyp2S.

34) Lines 192-225. The authors mention that glycosylation plays a crucial role in the antimicrobial activity of Hyp1 and Hyp2. However, I believe they don't mention anything about the state of the disulfide bonds present in those molecules. For palA it was shown that indeed disulfide bond formation was important for activity. I will like to know if the same is true for Hyp1 and Hyp2. Another possible explanation for their result is that the oxidation state between unmodified Hyp1/Hyp2 and unmodified leaderless Hyp1/Hyp2 is different leading to their difference in activity. I think further clarification from the authors is needed.

Answer to reviewer

As we mentioned in the manuscript, unmodified precursors pre-Hyp1-His and pre-Hyp2-His did not show antibacterial activity as well as unmodified Hyp1-His and Hyp2-His core peptides derived after expression of only core peptide genes. Our theoretical mass calculations of the core peptides included Met1 in the sequences. We did not notice before that both peptides represented in mass spectrometry: Hyp1-His-Glc and Hyp2-His-Glc, have lost the Met1 (-131.2 Da). It means that the observed masses match the masses of glycosylated (+162 Da) core peptides of Hyp1 and Hyp2. We did an additional experiment to show the state of disulfide bonds in glycosylated and nonglycosylated Hyp1 Hyp2 peptides. We have shown that after the disruption of disulfide bonds in the pre-Hyp1-His-Glc and Hyp1-His-Glc core peptide the antibacterial activity is abolished. Also, by using IAA/TCEP assays we show that pre-Hyp1-His-Glc and Hyp-His-Glc have disulfide bonds.

Moreover, we found out that our previous results showing that Hyp2-His-Glc core peptide has antibacterial activity is false. We assume that during purification of Hyp2-His core peptide we contaminated the sample with Hyp1-His-Glc core peptide, which

retained in the column after previous purification. We repeated experiments and we found out that Hyp2-His-Glc core peptide has no antibacterial activity.

35) Line 214. How do the authors know that the antimicrobial activity is due to the glycosylation or due to the presence of disulfide bonds on these molecules? I think the authors need to further clarify if when they coexpressed HypA peptides if they are able to obtain the disulfide linkages installed. See comment before.

Answer to reviewer

See the answer to question 34

36) Line 239-245. The authors mention MIC values for pallidocin but there is no data associated with those values. This reviewer believes that having the graphs used to calculate MIC in either the main text or supplementary material will help the reader.

Answer to reviewer

See the answer to question 15.

37) Line 252-253. I believe this statement is too strong and too bold. While the authors are able to express the whole gene cluster in E. coli, the authors failed to recognize and acknowledge another study were the genes involved in sublancin biosynthesis were expressed in E coli leading to sublancin production in E.coli, hence not being the first (ACS Chem. Biol., 2017, 12 (12), pp 2965–2969). The authors could argue that there were the first one to clone and express the whole gene of a glycocin in a heterologous host, but if we consider the final result, which is the production of a glyccocin in E coli, I believe the other study needs to be acknowledge. I also think further clarification on this statement is needed from the authors.

Changes in Discussion section

Here, we show for the first time that the whole glycocin biosynthetic gene cluster, derived from a thermophilic bacterium, can be cloned and functionally expressed in a heterologous host – *E. coli* BL21(DE3). Surprisingly, mature bacteriocin (glycosylated, oxidatively folded and leaderless) from Gram-positive bacteria could be synthesized and secreted by this Gram-negative host.

Previously, only full maturation of recombinant glycocins was reported *in vitro* for thurandacin and sublancin. Glycosylation and leader cleavage was performed enzymatically, followed by chemical oxidative folding[20,21]. The *in vitro* experiments limit the yield of the end product, are time consuming and expensive. Recently, a system was

developed for the heterologous expression of sublancin in *E. coli* SHuffle T7 Express cells that *in vivo* installs the glycosylation and oxidative folding following a single *in vitro* step of proteolytic leader cleavage[4]. SHuffle T7 Express strain expresses the disulfide bond isomerase DsbC, aiding oxidative folding of proteins in the cytoplasm[22]. We have included this information and reference to the manuscript. Here we demonstrate a different *in vivo* heterologous expression system for completely mature novel glycocins in *E. coli* BL21(DE3), evading the *in vitro* chemical and enzymatical steps.

With the help of PalS and PalT we can synthesize completely mature and active pallidocin, which is glycosylated, oxidatively folded and leaderless. Because pallidocin glycosyltransferase has a flexible substrate selectivity we propose that PalS could be a good tool for *in vivo* biosynthesis and screening of novel glycocins, as we showed with Hyp1 and Hyp2. This approach demonstrates that after *in vivo* peptide glycosylation the disulfide bonds most probably are formed spontaneously during the purification process. It means that the *in vitro* chemical oxidative folding is not absolutely necessary. Moreover, the *in vivo* glycosylation of core peptides evades the enzymatical leader cleavage.

Overall I think with these additional suggestions the manuscript will be strengthened.
Manuel A Ortega
Thank you for the very helpful comments.

Reviewer #3 (Remarks to the Author):
What are the major claims of the paper?
In their manuscript Kaunietis et al., describe the discovery of a new ribosomally synthesized and post-translationally modified peptide (RiPP): pallidocin. Pallidocin belongs to the small class of glycocins and has been heterologously expressed in E. coli. Furthermore two other gene clusters encoding new glycocins have been identified in two Bacillus strains and have been co expressed with pallidocin enzymes to produce glycocins in vivo.
The manuscript provides interesting findings for the scientific community with some insights into the biosynthesis of glycocins. However, some improvements could be done concerning the structure and presentation of the data. It took me some time and efforts to understand the novelty of the scientific data presented in the manuscript. Maybe that could be

addressed by pointing out more clearly the results in the manuscript compared to past studies.

38) There exists, for example, a working in vitro system to produce glycocins (Oman et al., Nat Chem Biol. 2011 Feb; 7(2): 78–80.) What exactly is the advantage of the in vivo system (maybe yields, or handling)? Also, there are established in vivo expression system established for other RiPP classes auch as lantibiotics (Caetano et. al., Appl Environ Microbiol. 2011 Jul; 77(14): 5023–5026), microviridins (Weiz et al., Chem Biol. 2011 Nov 23;18(11):1413-21), and cyanobactins (Schmidt et al., Proc Natl Acad Sci U S A. 2005 May 17;102(20):7315-20). How unique is the expression system presented here? What new things can we learn regarding the biosynthesis of glycocins, especially the promiscuity of PalS compared to SunS? It would be interesting to see how common sublancin type glycocins are in the bacterial kingdom?

Answer to reviewer

See the answers to question 19, 33, 37.

39) Furthermore, I am not sure how to interpret the results of the co-expression of the core_hip genes with palS (page 11). According to the authors both peptides showed antibacterial activity even when not glycosylated. The authors suggested that the glycosylation rate of the peptides were too low for detection. As far as I understand MS is very sensitive and glycosylation patterns relatively easy to detect. Could there be another explanation?

Answer to reviewer

See the answer to question 34

Minor points:

40) The scientific community calls these peptides RiPPs. Is there any reason the authors don't use this term?

Changes in Introduction section

Ribosomally synthesized and post-translationally modified peptides (RiPPs) are produced in all three domains of life. Part of the RiPPs overlap with a group of antibacterial peptides produced by bacteria, and this group historically is designated as *bacteriocins*[31–33]. We could also use RiPPs; no reason to avoid it.

41) It would be interesting to know more details about the specific activity of pallidocins against thermophilic bacteria. Any reason how the mode of action is there?

Answer to reviewer

It is the first report on glycocin's antibacterial activity against thermophilic bacteria. The mode of action is not revealed against neither these thermophilic, neither other bacteria. However, studies on sublancin and glycocin F have reported that a specific phosphoenolpyruvate:sugar phosphotransferase system (PTS) of *Bacillus* sp. bacteria is an important factor affecting their antibacterial activities (It is mentioned in introduction).

42) P5 l94/94: It seems unusual to me that the yield is enough for activity screening and structure elucidation but not for quantification at 280 nm.

Answer to reviewer

As we showed, the activity of pallidocin is extremely high. It means that the bacteriocin is active at a very low concentration. Quantification by abortion at 280 nm depends on composition of amino acids of the peptide, the absorptivity (extinction coefficient) of these amino acids, on the sensitivity of the machine and on the quantity of the peptide in a solution. These reasons limit the lowest concentration of the peptide that can be quantified. The extinction coefficient of pallidocin, which is calculated based on the amino acid composition, is relatively low. This is why quantity of pallidocin was sufficient for antibacterial activity, mass spectrometry but not for quantification by absorption at 280 nm.

43) The authors talk always about proposed structures. How sure are you about the exact structure of the compound?

Answer to reviewer

All glycocins characterized to date have 5 Cys residues in the core peptide and form two disulfide bonds between them. Pallidocin, Hyp1 and Hyp2 core peptides have also 5 Cys residues. We did additional experiments and showed that these peptides are oxidatively folded. As mentioned in our manuscript, the secondary structure prediction tool PSIPRED[2] proposes that two α-helices are present in the PalA, Hyp1 and Hyp2 core peptides. Also, we did additional experiment – CD spectrometry of pallidocin and confirmed that pallidocin has α-helices. Moreover, the prediction that four out of five Cys residues reside in these helical structures is consistent with the NMR structures of

sublancin 168 and glycocin F. The latter glycocins have also two α-helices nested with two disulfide bonds[34,35].

References

1. Wang, H. & van der Donk, W. A. Substrate Selectivity of the Sublancin S-Glycosyltransferase. *J. Am. Chem. Soc.* **133,** 16394–16397 (2011).
2. Buchan, D. W. A., Minneci, F., Nugent, T. C. O., Bryson, K. & Jones, D. T. Scalable web services for the PSIPRED Protein Analysis Workbench. *Nucleic Acids Res.* **41,** W349–W357 (2013).
3. Ji, S., Li, W., Baloch, A., Wang, M. & Cao, B. Improved production of sublancin via introduction of three characteristic promoters into operon clusters responsible for this novel distinct glycopeptide biosynthesis. *Microb. Cell Fact.* **14,** 17 (2015).
4. Biswas, S., Garcia De Gonzalo, C. V., Repka, L. M. & van der Donk, W. A. Structure–Activity Relationships of the S-Linked Glycocin Sublancin. *ACS Chem. Biol.* **12,** 2965–2969 (2017).
5. Hsieh, Y. S. Y. *et al.* Synthesis of the Bacteriocin Glycopeptide Sublancin 168 and S -Glycosylated Variants. *Org. Lett.* **14,** 1910–1913 (2012).
6. Stepper, J. *et al.* Cysteine S -glycosylation, a new post-translational modification found in glycopeptide bacteriocins. *FEBS Lett.* **585,** 645–650 (2011).
7. Raussens, V., Ruysschaert, J.-M. & Goormaghtigh, E. Protein concentration is not an absolute prerequisite for the determination of secondary structure from circular dichroism spectra: a new scaling method. *Anal. Biochem.* **319,** 114–121 (2003).
8. Garg, N., Goto, Y., Chen, T. & van der Donk, W. A. Characterization of the stereochemical configuration of lanthionines formed by the lanthipeptide synthetase GeoM. *Biopolymers* **106,** 834–842 (2016).
9. Garg, N. *et al.* Mode of action and structure–activity relationship studies of geobacillin I. *J. Antibiot. (Tokyo).* **67,** 133–136 (2014).
10. Garg, N., Tang, W., Goto, Y., Nair, S. K. & van der Donk, W. A. Lantibiotics from Geobacillus thermodenitrificans. *Proc. Natl. Acad. Sci.* **109,** 5241–5246 (2012).
11. Martirani, L., Varcamonti, M., Naclerio, G. & De Felice, M. Purification and partial characterization of bacillocin 490, a novel bacteriocin produced by a thermophilic

strain of Bacillus licheniformis. *Microb. Cell Fact.* **1,** 1 (2002).

12. Abdel-Mmohsein, H. S., Sasaki, T., Tada, C. & Nakai, Y. Characterization and partial purification of a bacteriocin-like substance produced by thermophilic Bacillus licheniformis H1 isolated from cow manure compost. *Anim. Sci. J.* **82,** 340–351 (2011).
13. Korenblum, E. *et al.* Production of antimicrobial substances by Bacillus subtilis LFE-1, B. firmus H2O-1 and B. licheniformis T6-5 isolated from an oil reservoir in Brazil. *J. Appl. Microbiol.* **98,** 667–675 (2005).
14. Cladera-Olivera, F., Caron, G. R. & Brandelli, A. Bacteriocin-like substance production by Bacillus licheniformis strain P40. *Lett. Appl. Microbiol.* **38,** 251–256 (2004).
15. Sharp RJ, Bingham AHA, Comer MJ, Atkinson A. Partial characterization of a bacteriocin (Thermocin) from Bacillus stearothermophilus RS93. *J. Gen. Microbiol.* 449–451 (1979).
16. Yule, R. & Barridge, B. D. Isolation and characterization of a bacteriocin produced by Bacillus stearothermophilus strain NU-10. *Can. J. Microbiol.* **22,** 1743–50 (1976).
17. Shafia, F. Thermocins of Bacillus stearothermophilus. *J. Bacteriol.* **92,** 524–5 (1966).
18. Pranckute, R. *et al.* Differences of antibacterial activity spectra and properties of bacteriocins, produced by Geobacillus sp. bacteria isolated from different environments. *J. Microbiol. Biotechnol. Food Sci.* **05,** 155–161 (2015).
19. Pranckutė, R. *Evaluation of Lactic Acid and Thermophilic Bacteria Antibacterial Activity and Compatibility with Prebiotic*. (2017).
20. Wang, H. *et al.* The Glycosyltransferase Involved in Thurandacin Biosynthesis Catalyzes Both O- and S-Glycosylation. *J. Am. Chem. Soc.* **136,** 84–87 (2014).
21. Oman, T. J., Boettcher, J. M., Wang, H., Okalibe, X. N. & van der Donk, W. A. Sublancin is not a lantibiotic but an S-linked glycopeptide. *Nat. Chem. Biol.* **7,** 78–80 (2011).
22. Lobstein, J. *et al.* SHuffle, a novel Escherichia coli protein expression strain capable of correctly folding disulfide bonded proteins in its cytoplasm. *Microb. Cell Fact.* **11,** 753 (2012).
23. Mark, A. & Payton. Production of ethanol by thermophilic bacteria. *Trends in Bioteehnology* **2,** 153–158 (1984).
24. Ahmad, A. *et al.* A Genome Scale Model of Geobacillus thermoglucosidasius (C56-YS93) reveals its biotechnological potential on rice straw hydrolysate. *J. Biotechnol.* **251,** 30–37 (2017).

25. Jiang, Y. *et al.* State of the art review of biofuels production from lignocellulose by thermophilic bacteria. *Bioresour. Technol.* **245,** 1498–1506 (2017).
26. Burgess, S. A., Lindsay, D. & Flint, S. H. Thermophilic bacilli and their importance in dairy processing. *Int. J. Food Microbiol.* **144,** 215–225 (2010).
27. Maky, M. A. *et al.* Enterocin F4-9, a Novel O -Linked Glycosylated Bacteriocin. *Appl. Environ. Microbiol.* **81,** 4819–4826 (2015).
28. Berkmen, M. Production of disulfide-bonded proteins in Escherichia coli. *Protein Expr. Purif.* **82,** 240–251 (2012).
29. Wu, X. *et al.* Optimal Cleavage and Oxidative Folding of α-Conotoxin TxIB as a Therapeutic Candidate Peptide. *Mar. Drugs* **11,** 3537–3553 (2013).
30. Lin, C. C.-J. & Chang, J.-Y. Pathway of Oxidative Folding of Secretory Leucocyte Protease Inhibitor: An 8-Disulfide Protein Exhibits a Unique Mechanism of Folding †. *Biochemistry* **45,** 6231–6240 (2006).
31. Alvarez-Sieiro, P., Montalbán-López, M., Mu, D. & Kuipers, O. P. Bacteriocins of lactic acid bacteria: extending the family. *Appl. Microbiol. Biotechnol.* **100,** 2939–2951 (2016).
32. Cotter, P. D., Ross, R. P. & Hill, C. Bacteriocins - a viable alternative to antibiotics? *Nat. Rev. Microbiol.* **11,** 95–105 (2013).
33. Arnison, P. G. *et al.* Ribosomally synthesized and post-translationally modified peptide natural products: overview and recommendations for a universal nomenclature. *Nat. Prod. Rep.* **30,** 108–160 (2013).
34. Garcia De Gonzalo, C. V., Zhu, L., Oman, T. J. & van der Donk, W. A. NMR Structure of the S-Linked Glycopeptide Sublancin 168. *ACS Chem. Biol.* **9,** 796–801 (2014).
35. Venugopal, H. *et al.* Structural, Dynamic, and Chemical Characterization of a Novel S-Glycosylated Bacteriocin. *Biochemistry* **50,** 2748–2755 (2011).

Reviewers' comments:

Reviewer #1 (Remarks to the Author):

Review of resubmitted paper.

While the authors have made some improvements to this manuscript and added evidence from new experiments, in my opinion there are still many questions that need to be answered before the work can be accepted for publication.

In answer to my comment 1. I acknowledge that sequencing is the ultimate confirmation of successful cloning and expression of genes, but no sequences were provided. While there is evidence for the successful expression of the main products in E. coli, there is only circumstantial evidence, (the production of an active product of approximately the correct mass) that all the genes in the pallidocin cluster were expressed. I do not think it unreasonable to expect this. A single gel with all the gene products would be appropriate. The extra material added to the methods section of the supplementary information does go some way to overcome my concerns.

In answer to point 2, the controls I was concerned about were the controls that have now been shown in figure 3.

3. I accept the rebuttal.

4. This question was answered and appropriate changes made to the supplementary information.

5. I agree that the authors have shown that they did produce an antimicrobial compound that is modified by a hexose attached to cysteine 25. I am less convinced about the disulfide bonds as the mass spectrometry results do not provide unequivocal confirmation of the presence of 2 bonds in all spectra.

6 and 7. These new figures (3) show the fragmentation, but are not all that convincing to my mind as the fragment ion s appear to be of very low intensity, with many more intense ions not being identified. As similar results have been obtained in two separate trials, the evidence of glycosylation of Cys 25 is supported, but the spectra are very noisy, with poor S/N for most of the fragment ions of interest. The ions should be labelled as b, y and a ions.

Questions 8 a-d
Firstly, the authors are not correct in stating that disulfide bond formation is not a PTM. It is a PTM, and for the glycocins a very important one. I think they have shown that PalA, Hyp1 and Hyp 2 are indeed modified by a hexose, and they have identified this hexose as glucose making PalA a sublancin-like glycocin. Firstly, the addition of Figure 6 is very helpful to the reader and helps with interpretation of the mass spectrometry results. The only problem is these are the average masses, and are referred to as [M+H]+ ions in all the mass spectra. If this was the case they are not correctly referred to on the spectra. I applaud the authors providing ESI spectra collected on a Q Exactive, but to be absolutely sure of the conclusions drawn they should be showing the monoisotopic mass of one of the characteristic ions of the compound. (Refer to Stepper at al 2011.) What is shown here, I think are deconvoluted spectra giving M, not M+H, but I can’t be sure as no details have been given as to how the data have been processed, or indeed of the conditions used for data collection and fragmentation. This must be done before the paper can be accepted for publication. Similarly, was the

Voyager in reflectron or linear mode? And how was the data processed if the former. What was the mass range what were the details of data collection?

There are several other glaring errors. All masses have the decimal point replaced with a comma making the masses nonsensical.

Supplementary Figure 7.
The difference of 3 amu between the glycosylated and non-glycosylated pre-palA-His-Glc –(2 SS) is put down as machine error. It could equally represent the formation of only 1 disulfide or even none. Furthermore, the adducts listed at the bottom of the figure have the wrong masses: Na +23, Met oxidation +16, methylation +14

Supplementary Figure 8
Repeat of Figure 7 on a more accurate ESI instrument.

The average mass of pre-palA-His is given as 7877.93, yet the mass observed is 7874.48. This gives a mass difference of 3.45, which although near to 4 is not accurate. In (b) the addition of 162.05 is correct, showing PalA is glycosylated with a hexose. Fig 8c shows the correct mass for the mature PalA. However, no mention is made of the large peak at about 8500 amu?
Supplementary Figure 9. Again the observed average mass of pre-His-Xa-palA-Glc-is calculated to be 8644.59, on the TOF (should it be 8645.59 for [M+H]+) which is the average mass less 4H for the disulfide bonds. What is observed is a mass of 8516 said to be the expected average mass less the mass of the first Met, 131.2 amu. However the mass difference is 128.55 amu. Clearly not methionine unless the 2 disulfide bonds are not formed (more likely). (b) and (c), are said to represent the mature pallidocin molecule and both spectra have a peak at ~4221 amu. Yet the mass given for the peptide in Figure 6 is 5137.77. Bearing in mind that including the tag, there are 48 amino acids and using the average amino acid mass of 110, we would expect a mass of ~ 5280, so 5137.77 seems reasonable. If the peptide loses 4 amu because of the formation of 2 disulfide bonds, then gains 162.05 due to glycosylation, we would expect 5295.77 amu. If the N-terminal Met is missing, the expected mass would be 5164.57. If the tag does not include a GG, there would be a further loss of 114 to give ~5050. Clearly none of these masses match the observed mass of 4221.

Supplementary Figure 11.
The authors checked their system using the sublancin model using MALDI. The mass of 6834.81 is claimed to represent pre-SunA-his with 2 methionine oxidations. Figure 6 shows that pre-SunA-His does not have 2 methionines, only a single one. Again there is a difference of 2 amu between the observed and calculated masses of the unmodified pre-sunA-His.

Supplementary Figure 12.
While these ESI spectra convincingly show the modification of SunA by PalS there is once more doubt about the formation of disulfides with a mass difference of 3 rather than the 4 expected for the formation of 2 disulfide bonds.

Supplementary Figure 13.
The mass of 7934.69 represents a mass difference of 18, not 23 amu thus the addition of water rather than Na. Again, justifying the increase in mass of 16 as Met oxidation without evidence is risky. Sequencing using MSMS would clarify these modifications.
Supplementary Figure 14. Repeat of the analysis in Fig 13 confirms the addition of a hexose, but indicates the presence of only a single disulfide (7916-7914) and (8076-8074)
9., Figure 2 does not reflect the assignment of secondary structure, and it is not an output from

PSIPRED. As far as I can see it is modelled on sequence homology to sublancin. However it is good to see a far UV CD spectrum.

Questions 10-12. Points raised have been corrected.

Questions 13.I take the authors point. But the question of the number of disulfide present can be accurately determined using monoisotopic mass spectrometry. However the reduction/alkylation experiments reported in Supplementary tables 2-4 confirm the presence of disulfide bonds.

Questions 14-15 have been addressed and I am happy with the changes.

Question 16. The article still needs a very careful edit as there are many grammatical errors, especially in the supplementary info. I

Overall the authors have answered many of my questions and have amended both the main paper and the supplementary information. However there are careless mistakes in the mass spectrometry evidence, enough to make me doubt this evidence, and I do not recommend publication until these are fixed.

Reviewer #2 (Remarks to the Author):

The authors have addressed the comments made by the reviewers. However, I do have a couple of suggestions:

1. On several occasions instead of the word "cytoplasm" the authors used "citoplasm" not sure if its a spelling error or regional use.

2. Upon the addition of the new data on the antimicrobial activity of modified Hyp1 and Hyp2, the authors did not include within the discussion section as to why Hyp2 does not show antimicrobial activity but Hyp1 does. I think it will be interesting if the authors explore the differences between these peptides that might explain the differences in biological activities.

3. On the figure legend of several ms spectra, the authors used the phrase" within an error" and I think it should read "within error"

As it is written the work presented here seems interesting and in my opinion will be of broad interest to the RiPP and natural product community.

Reviewer #3 (Remarks to the Author):

My concerns have been adequately addressed. The manuscript reads much better now.

Reviewers' comments

Reviewer #1 (Remarks to the Author)

Reviewer:
While the authors have made some improvements to this manuscript and added evidence from new experiments, in my opinion there are still many questions that need to be answered before the work can be accepted for publication.

Comment 1. In answer to my comment 1 (rebuttal 1), I acknowledge that sequencing is the ultimate confirmation of successful cloning and expression of genes, but no sequences were provided. While there is evidence for the successful expression of the main products in E. coli, there is only circumstantial evidence, (the production of an active product of approximately the correct mass) that all the genes in the pallidocin cluster were expressed. I do not think it unreasonable to expect this. A single gel with all the gene products would be appropriate. The extra material added to the methods section of the supplementary information does go some way to overcome my concerns.
Author's answer:
In addition to the DNA sequencing of the constructs with cloned fragments in the vectors, that was discussed and explained before in Materials and Methods of Supplementary Information, we now provide extra evidence that validates the constructs. As reviewer #1 requested in comment 1 of the 1st and 2nd revision of manuscript, we did a PCR that confirms the constructs *e.g.* that all genes of the *pal* gene cluster are present in constructed pBAD24-pal vector. These data are provided in the new Supplementary Figs. 1, 7 and 13.

Reviewer:
Comment 2. In answer to point 2 (rebuttal 1), the controls I was concerned about were the controls that have now been shown in figure 3.
Author's answer:
Reviewer is satisfied with the improvements.

Reviewer:
Comment 3. I accept the rebuttal (question 3 in rebuttal 1).
Author's answer:
Reviewer is satisfied with the improvements.

Reviewer:

Comment 4. This question (4) was answered and appropriate changes made to the supplementary information.

Author's answer:

Reviewer is satisfied with the improvements.

Reviewer:

Comment 5 (questions 6 and 7 in rebuttal 1). I agree that the authors have shown that they did produce an antimicrobial compound that is modified by a hexose attached to cysteine 25. I am less convinced about the disulfide bonds as the mass spectrometry results do not provide unequivocal confirmation of the presence of 2 bonds in all spectra.

Author's answer:

The disulfide bonds of mature pallidocin without His-tag (PalA-Glc), which was derived after expression of the whole biosynthetic *pal* gene cluster, was confirmed by high resolution LC-ESI-MS and these results are clear and unequivocal (Supplementary Fig. 3).

In addition, experiments with reduction/alkylation confirmed the presence of two disulfide bonds in the His-tagged peptides. Moreover, the reduction and alkylation of Cys resulted in the loss of antibacterial activity confirming the importance of the disulfide bonds. These results are represented in Supplementary Tables 2, 3 and 4. We included new Supplementary Figs. 10, 15, 17, 19 and 20 representing LC-ESI-MS results with isotopic masses, which confirm that the synthesized peptides are with two disulfide bonds.

Reviewer:

Comments 6 and 7 (questions 6 and 7 in rebuttal 1). These new figures (3) show the fragmentation, but are not all that convincing to my mind as the fragment ion s appear to be of very low intensity, with many more intense ions not being identified. As similar results have been obtained in two separate trials, the evidence of glycosylation of Cys 25 is supported, but the spectra are very noisy, with poor S/N for most of the fragment ions of interest. The ions should be labelled as b, y and a ions.

Author's answer:

As the reviewer requested, ions: **b**, **a** and **y,** are now labelled and represented in new Supplementary Fig. 4. We are happy that the reviewer is convinced with the additional evidence we provided before (trial 2) that the Cys25 is glycosylated. Unfortunately, we could not get better spectra with lower noise (S/N). Some other unlabeled peaks can represent fragment ions without glucose as the sugar may be detached during the fragmentation. Similar MS/MS spectra

patterns have been observed in analysis of sublancin (Nature Chemical Biology, 7(2):78-80, 2011).

Reviewer:

Comment 8 (questions 8 a-d in rebuttal 1). Firstly, the authors are not correct in stating that disulfide bond formation is not a PTM. It is a PTM, and for the glycocins a very important one.

I think they have shown that PalA, Hyp1 and Hyp 2 are indeed modified by a hexose, and they have identified this hexose as glucose making PalA a sublancin-like glycocin. Firstly, the addition of Figure 6 is very helpful to the reader and helps with interpretation of the mass spectrometry results. The only problem is these are the average masses, and are referred to as [M+H]+ ions in all the mass spectra. If this was the case they are not correctly referred to on the spectra. I applaud the authors providing ESI spectra collected on a Q Exactive, but to be absolutely sure of the conclusions drawn they should be showing the monoisotopic mass of one of the characteristic ions of the compound. (Refer to Stepper at al 2011.) What is shown here, I think are deconvoluted spectra giving M, not M+H, but I can’t be sure as no details have been given as to how the data have been processed, or indeed of the conditions used for data collection and fragmentation. This must be done before the paper can be accepted for publication.

Similarly, was the Voyager in reflectron or linear mode? And how was the data processed if the former. What was the mass range what were the details of data collection?

There are several other glaring errors. All masses have the decimal point replaced with a comma making the masses nonsensical.

Author’s answer:

We agree that disulfide bond formation is a PTM. This statement is now corrected accordingly in the descriptions of all Supplementary Figures. In addition, we have replaced decimal separators to the points in the text.

All the masses (monoisotopic and average) are now recalculated as $[M+H]^+$ ions and added to the new Supplementary Fig 8. The MALDI-TOF-MS spectra are represented as $[M+H]^+$ ions now. We have removed all the text about the average masses from the descriptions of Supplementary Figures representing LC-ESI-MS spectra. As the reviewer suggested before, to be absolutely sure about the masses of peptides and conclusions we make, now we focus only on the monoisotopic masses. We admit that our evaluations and interpretations of average masses in the LC-ESI-MS spectra were not correct and we thank the referee for pointing this out.

New figures of the LC-ESI-MS spectra represented as $[M+H]^+$, and also as $[M+H]^{+7}$ or $[M+H]^{+10}$ ions, are included now. This is due to the deconvolution algorithm did not work very well for larger peptides/proteins and the software could not recognize the first isotope correctly because the intensity was low.

More details about conditions of mass spectrometry analysis are now provided in Supplementary Information file in the section of *Mass spectrometry analysis.*

In addition to the new Supplementary Figures of LC-ESI-MS analysis that confirm the presence of disulfide bonds in the peptides, we note that these bonds were also confirmed by an alternative method – reduction/alkylation of Cys prior the MALDI-TOF-MS analysis. These results are represented in Supplementary Tables 2, 3 and 4. However, the fact that reducing agents abolish the activity of our product is convincing evidence that shows that the disulfides are there and necessary for activity, as they are also in other glycocins.

Reviewer:

Supplementary Figure 7. The difference of 3 amu (+2.21 Da) between the glycosylated and non-glycosylated pre-palA-His-Glc –(2 SS) is put down as machine error. It could equally represent the formation of only 1 disulfide or even none.

Furthermore, the adducts listed at the bottom of the figure have the wrong masses: Na +23, Met oxidation +16, methylation +14.

Author's answer:

The masses of adducts in the figures have been corrected.

Supplementary Figure 7 represented analysis by MALDI-TOF-MS. The accuracy for the machine is >0.05% of sample mass. This resolution is not good enough for disulfide bond confirmation. MALDI-TOF-MS was used as an initial screening and inspection method to observe the glycosylation and the leader cleavage of the peptides. The disulfide bonds in the peptides were confirmed by reduction/alkylation of Cys prior the MALDI-TOF-MS analysis, these results are represented in Supplementary Tables 2, 3 and 4. Using this method similar mass variations were observed as in the analysis of sublancin (Journal of American Chemical Society, 133(41):16394-7, 2011.).

Reviewer:

Supplementary Figure 8. Repeat of Figure 7 on a more accurate ESI instrument. The average mass of pre-palA-His is given as 7877.93, yet the mass observed is 7874.48. This gives a mass difference of 3.45, which although near to 4 is not accurate. In (b) the addition of 162.05 is correct, showing PalA is glycosylated with a hexose. Fig 8c shows the correct mass for the mature PalA. However, no

mention is made of the large peak at about 8500 amu?

Author's answer:

The large peak in the Figure 8c represents unknown compound with a mass of approximately 8465.19 Da. It could be a residual peptide in the column, which could appear there from a previous run and analysis on the LC-ESI-MS machine. Nevertheless, our interest is the peak with a mass of approximately 5296.24 Da. We note that these data are now represented in new Supplementary Fig. 10 with isotopic masses, and it confirms the presence of disulfide bonds in the peptides. We note that these bonds were also confirmed by an alternative method – reduction/alkylation of Cys prior the MALDI-TOF-MS analysis. These results are represented in Supplementary Tables 2, 3 and 4. However, the fact that reducing agents abolish the activity of our product is convincing evidence that shows that the disulfides are there and necessary for activity, as they are also in other glycocins.

Reviewer:

Supplementary Figure 9. Again the observed average mass of pre-His-Xa-palA-Glc-is calculated to be 8644.59, on the TOF (should it be 8645.59 for [M+H]+) which is the average mass less 4H for the disulfide bonds. What is observed is a mass of 8516 said to be the expected average mass less the mass of the first Met, 131.2 amu. However the mass difference is 128.55 amu. Clearly not methionine unless the 2 disulfide bonds are not formed (more likely).

Author's answer:

Supplementary Figures 9a and 9b represent analysis by MALDI-TOF-MS. As mentioned before, the accuracy for the machine is >0.05% of sample mass. The observed mass difference of +2.65 Da should be tolerated as the machine error. Using this method similar mass variations were observed in analysis of sublancin (Journal of American Chemical Society, 133(41):16394-7, 2011.).

Analysis represented in Supplementary Figure 9a was performed to select elution fractions with the glycosylated precursor peptide (pre-His-Xa-PalA-Glc) after RP-HPLC analysis, and to inspect the purity of the peptide for a subsequent experiments. Importantly, the final product after the leader cleavage – mature pallidocin (PalA-Glc, without His-tag), was analyzed by LC-ESI-MS (Supplementary Figure 9c) and the obtained ion mass matched the expected monoisotopic mass of mature pallidocin with two disulfide bonds. This figure is now represented in the new Supplementary Figure 11.

Reviewer:

Supplementary Figure 9 (b) and (c), are said to represent the mature pallidocin molecule and both spectra have a peak at ~4221 amu. Yet the mass given for the peptide in Figure 6 is 5137.77. Bearing in mind that including the tag, there are 48 amino acids and using the average amino acid mass of 110, we would expect a

mass of ~ 5280, so 5137.77 seems reasonable. If the peptide loses 4 amu because of the formation of 2 disulfide bonds, then gains 162.05 due to glycosylation, we would expect 5295.77 amu. If the N-terminal Met is missing, the expected mass would be 5164.57. If the tag does not include a GG, there would be a further loss of 114 to give ~5050. Clearly none of these masses match the observed mass of 4221.

Author's answer:

The reviewer is confused between peptides PalA-His-Glc (mature pallidocin core peptide with His-tag in C-terminus, $[M+H]^+$ avg. 5296.89 Da) and PalA-Glc (mature pallidocin core peptide without His-tag, $[M+H]^+$ avg. 4222.8 Da). The glycosylated precursor peptide pre-His-Xa-PalA-Glc has a His-tag in the N-terminus, which was later cleaved together with the leader sequence. The synthesis of pallidocin in this approach resulted in PalA-Glc – the mature pallidocin without His-tag (4222.8 Da); it is the same peptide as been derived after the expression of the whole pallidocin biosynthetic gene cluster. This figure is now represented in the new Supplementary Figure 11.

Amino acid sequences of PalA-His and pre-His-Xa-PalA are represented in the Supplementary Figure 8. This figure also indicates the positions of His-tag, leader and core peptide sequences.

Reviewer:

Supplementary Figure 11. The authors checked their system using the sublancin model using MALDI. The mass of 6834.81 is claimed to represent pre-SunA-his with 2 methionine oxidations. Figure 6 shows that pre-SunA-His does not have 2 methionines, only a single one. Again there is a difference of 2 amu between the observed and calculated masses of the unmodified pre-sunA-His.

Author's answer:

Supplementary Figure 11 represented analysis by MALDI-TOF-MS. As mentioned before, the accuracy for the machine is >0.05% of sample mass. The mass of main peak ($[M+H]^+$ – 6802.81 Da) in the Supplementary Figure 11a match the mass of pre-SunA-His with two disulfide bonds ($[M+H]^+$ avg. – 6801.84 Da) with variation of +0.97 Da, which can be accepted as the machine error.

The mass of the second lower intensity peak ($[M+H]^+$ – 6834.54 Da) match the mass of the same peptide with adducts. The difference of +32.70 Da between theoretical ($[M+H]^+$ avg. – 6801.84 Da) and observed ($[M+H]^+$ – 6834.54 Da) masses might be caused by: water (+18 Da) and methionine oxidation (+16 Da) including machine error (-1.30 Da). The reviewer is correct, the pre-SunA-His has only one Met, so there can be only one Met oxidation. It is complicated to determine precise masses of the peptides because of mass variations introduced due to instrument inaccuracy or adducts in the peptides. These data are represented in the new Supplementary Fig. 14.

Reviewer:

Supplementary Figure 12. While these ESI spectra convincingly show the modification of SunA by PalS there is once more doubt about the formation of disulfides with a mass difference of 3 rather than the 4 expected for the formation of 2 disulfide bonds.

Author's answer:

As the reviewer suggested above, to be absolutely sure about the masses of peptides and conclusions we make, we focus only on monoisotopic masses, now. We admit that our evaluations and interpretations of average masses in LC-ESI-MS spectra were incorrect. The new Supplementary Fig. 15 with isotopic masses confirms the presence of disulfide bonds.

Reviewer:

Supplementary Figure 13. The mass of 7934.69 represents a mass difference of 18, not 23 amu thus the addition of water rather than Na. Again, justifying the increase in mass of 16 as Met oxidation without evidence is risky. Sequencing using MSMS would clarify these modifications.

Author's answer:

Supplementary Fig. 13 represented analysis by MALDI-TOF-MS. The reviewer is correct, the mass difference between theoretical ($[M+H]^+$ - 7917.14 Da) and observed ($[M+H]^+$ - 7934.69 Da) masses is +17.55 Da. It might represent the addition of water (+18 Da) with a variation (-0.45 Da). On the other hand, the observed mass matches the peptide with two disulfide bonds (-4 Da) and sodium adduct (+22 Da) with a variation (-0.45 Da). As mentioned before, the accuracy for the machine is >0.05% of sample mass, and using this method it is complicated to accurately determine the masses of peptides. These data are now represented in new Supplementary Fig. 16.

Reviewer:

Supplementary Figure 14. Repeat of the analysis in Fig 13 confirms the addition of a hexose, but indicates the presence of only a single disulfide (7916-7914) and (8076-8074).

Author's answer:

As the reviewer suggested above, to be absolutely sure about the masses of peptides and conclusions we make, we focus only on monoisotopic masses, now. We admit that our evaluations and interpretations of average masses in LC-ESI-MS spectra were incorrect. The new Supplementary Fig. 17 with monoisotopic masses confirms the presence of disulfide bonds.

Moreover, two disulfide bonds were confirmed by an alternative method: reduction/alkylation of Cys prior the MALDI-TOF analysis, these results are represented in Supplementary Table 3.

Reviewer:

Comment 9. Figure 2 does not reflect the assignment of secondary structure, and it

is not an output from PSIPRED. As far as I can see it is modelled on sequence homology to sublancin. However it is good to see a far UV CD spectrum.

Author's answer:

The Figure 2 is corrected now, and represents secondary structure of pallidocin, which is based and modeled according to PSIPRED calculations.

Reviewer:

Comment 10. Questions 10-12 in rebuttal 1. Points raised have been corrected.

Author's answer:

Reviewer is satisfied with improvements.

Reviewer:

Comment 11. Questions 13 in rebuttal 1.I take the authors point. But the question of the number of disulfide present can be accurately determined using monoisotopic mass spectrometry. However the reduction/alkylation experiments reported in Supplementary tables 2-4 confirm the presence of disulfide bonds.

Author's answer:

The new Supplementary Figures of LC-ESI-MS analysis represents monoisotopic masses, which confirms the presence of disulfide bonds in the peptides, now. In addition, two disulfide bonds in all the peptides were confirmed by an alternative method: reduction/alkylation of Cys prior the MALDI-TOF analysis, these results are represented in Supplementary Tables 2, 3 and 4. Moreover, the latter results showed the importance of these bonds for antibacterial activity.

Reviewer:

Comment 12. Questions 14-15 in rebuttal 1 have been addressed and I am happy with the changes.

Author's answer:

Reviewer is satisfied with improvements.

Reviewer:

Comment 13. Question 16 in rebuttal 1. The article still needs a very careful edit as there are many grammatical errors, especially in the supplementary info.

Author's answer:

Editing and careful check up was performed.

Reviewer:

Overall the authors have answered many of my questions and have amended both the main paper and the supplementary information. However there are careless mistakes in the mass spectrometry evidence, enough to make me doubt this evidence, and I do not recommend publication until these are fixed.

Reviewer #2 (Remarks to the Author)

Reviewer:

The authors have addressed the comments made by the reviewers. However, I do have a couple of suggestions:

Comment 14. On several occasions instead of the word "cytoplasm" the authors used "citoplasm" not sure if its a spelling error or regional use.

Author's answer:

Corrected.

Reviewer:

Comment 15. Upon the addition of the new data on the antimicrobial activity of modified Hyp1 and Hyp2, the authors did not include within the discussion section as to why Hyp2 does not show antimicrobial activity but Hyp1 does. I think it will be interesting if the authors explore the differences between these peptides that might explain the differences in biological activities.

Author's answer:

Changes in the Discussion section of manuscript:

All *sublancin*-type glycocins, including novel pallidocin, Hyp1 and glycosylated core peptide Hyp2, have relatively rich content of hydrophobic residues in N-terminus, and charged residues in C-terminus. Comparing the core peptides of glycocins, the Hyp2 has relatively long C-terminus "tail", not characteristic to other sublancin-type glycocins, and is relatively rich in charged residues (Glu20, Arg21, Arg22) in the interhelical loop (Fig. 2). These two features or one of them might be the reason why glycosylated Hyp2 core peptide and precursor did not have antibacterial activity against the strains tested. We cannot exclude the possibility that it has a different spectrum of activity, too. This could be the subject for future research on glycocins.

Reviewer:

Comment 16. On the figure legend of several ms spectra, the authors used the phrase" within an error" and I think it should read "within error".

Author's answer:

Corrected.

Reviewer:

As it is written the work presented here seems interesting and in my opinion will be of broad interest to the RiPP and natural product community.

Reviewer #3 (Remarks to the Author)

Reviewer:
My concerns have been adequately addressed. The manuscript reads much better now.

REVIEWERS' COMMENTS:

Reviewer #1 (Remarks to the Author):

Overall the authors have answered all my concerns. The manuscript reads much better now and only needs a few minor modifications for publication as shown in the attached modified rebuttal. I recommend that they remove all the MALDI spectra as they are now redundant. Absolute proof of the PTMs is given by the added ESI Q-Exactive spectra. I recommend that the masses of the various protein products can be calculated from a monoisotopic ion when deconvoluted spectra cannot b calulaed.

Other errors to be corrected are as follows:
Page 10 line 166 Should be supplementary Figure 9d and 10c . There is no 10d
Page 10 Line 176. Why on earth were these masses not measured on the Q-Exactive. These excuses for mass accuracy weaken the paper in my opinion.

Page 14 line 236. Remove Fig 2
Page 14 Line 238 add .... by two disulfide bonds giving rise to the predicted structures for Hyp1 and Hyp2 (Fig 2)
Page 15 Line 256 with 'the two new genes' replace with with the names of the genes.

Page 15, Line 260 . ‘was expresed in E. coli without palS co-expression. The recombinant hyp1-His core peptide’
Page 16 Line 280. A small nigle. I note there is no evidence presented for these clains in the supplementary information.
Page 17 Line 316 Reads better as ‘In vitro studies on the glycosylation of the sublancin precursor showed that S-glycosyltransferase…………’

Other minor corrections are noted in the attached document.

Editorial note: The attached document follows on the next page.

Reviewers' comments

Reviewer #1 (Remarks to the Author)

Reviewer:
While the authors have made some improvements to this manuscript and added evidence from new experiments, in my opinion there are still many questions that need to be answered before the work can be accepted for publication.

Comment 1. In answer to my comment 1 (rebuttal 1), I acknowledge that sequencing is the ultimate confirmation of successful cloning and expression of genes, but no sequences were provided. While there is evidence for the successful expression of the main products in E. coli, there is only circumstantial evidence, (the production of an active product of approximately the correct mass) that all the genes in the pallidocin cluster were expressed. I do not think it unreasonable to expect this. A single gel with all the gene products would be appropriate. The extra material added to the methods section of the supplementary information does go some way to overcome my concerns.

Author's answer:
In addition to the DNA sequencing of the constructs with cloned fragments in the vectors, that was discussed and explained before in Materials and Methods of Supplementary Information, we now provide extra evidence that validates the constructs. As reviewer #1 requested in comment 1 of the 1st and 2nd revision of manuscript, we did a PCR that confirms the constructs *e.g.* that all genes of the *pal* gene cluster are present in constructed pBAD24-pal vector. These data are provided in the new Supplementary Figs. 1, 7 and 13.

I am happy with these additional supplementary figures and references to them in the main document

Reviewer:
Comment 2. In answer to point 2 (rebuttal 1), the controls I was concerned about were the controls that have now been shown in figure 3.

Author's answer:
Reviewer is satisfied with the improvements.

Reviewer:
Comment 3. I accept the rebuttal (question 3 in rebuttal 1).

Author's answer:
Reviewer is satisfied with the improvements.

Reviewer:

Comment 4. This question (4) was answered and appropriate changes made to the supplementary information.

Author's answer:

Reviewer is satisfied with the improvements.

Reviewer:

Comment 5 (questions 6 and 7 in rebuttal 1). I agree that the authors have shown that they did produce an antimicrobial compound that is modified by a hexose attached to cysteine 25. I am less convinced about the disulfide bonds as the mass spectrometry results do not provide unequivocal confirmation of the presence of 2 bonds in all spectra.

Author's answer:

The disulfide bonds of mature pallidocin without His-tag (PalA-Glc), which was derived after expression of the whole biosynthetic *pal* gene cluster, was confirmed by high resolution LC-ESI-MS and these results are clear and unequivocal (Supplementary Fig. 3).

In addition, experiments with reduction/alkylation confirmed the presence of two disulfide bonds in the His-tagged peptides. Moreover, the reduction and alkylation of Cys resulted in the loss of antibacterial activity confirming the importance of the disulfide bonds. These results are represented in Supplementary Tables 2, 3 and 4. We included new Supplementary Figs. 10, 15, 17, 19 and 20 representing LC-ESI-MS results with isotopic masses, which confirm that the synthesized peptides are with two disulfide bonds.

These new mass spectra are satisfactory evidence for the author's claims of a **hexose** post translational modification, not a glucose, and the presence of 2 disulfide bonds. However they make the MALDI spectra redundant. I would therefore recommend removing supplementary figures 9, 11 a and b, 14, 16, 18.You may have used these as initial verification of modification, but all they really told you was there was a possibility of modification. These results should not be included in this paper. Population the captions with excuses for inaccuracy does not add to the manuscript, and in my view actually detracts from it. The real proof is in the ESI Spectra and the monoisotopic masses. As the MALDI spectra were used to report the reduced/alkylated masses in supplementary tables 2-4 details of its use can remain in the supplementary methods. The captions of figures 10 a and b need to be modified by removing the note about deconvolution. Deconvolution is not necessary, as calculation of the full mass from these monoisotopic ions is possible and gives 8031.61 (M+H) for pre-palA-His-Hex cf theoretical monoisotopic mass of 8031.58. (10b) and 7869.49 cf 7869.50 for pre-PalA-His (10a)

Reviewer:

Comments 6 and 7 (questions 6 and 7 in rebuttal 1). These new figures (3) show the fragmentation, but are not all that convincing to my mind as the fragment ion s appear to be of very low intensity, with many more intense ions not being identified. As similar results have been obtained in two separatetrials, the evidence of glycosylation of Cys 25 is supported, but the spectra are very noisy, with poor S/N for most of the fragment ions of interest. The ions should be labelled as b, y and a ions.

Author's answer:

As the reviewer requested, ions: **b**, **a** and **y,** are now labelled and represented in new Supplementary Fig. 4. We are happy that the reviewer is convinced with the additional evidence we provided before (trial 2) that the Cys25 is glycosylated. Unfortunately, we could not get better spectra with lower noise (S/N). Some other unlabeled peaks can represent fragment ions without glucose as the sugar may be detached during the fragmentation. Similar MS/MS spectra patterns have been observed in analysis of sublancin (Nature Chemical Biology, 7(2):78-80, 2011).

I am happy with the labeling of these ions. Perhaps a table of the ions found as is produced by proteome discoverer or mascot would be informative. I accept that the authors could not obtain better spectra.

Reviewer:

Comment 8 (questions 8 a-d in rebuttal 1). Firstly, the authors are not correct in stating that disulfide bond formation is not a PTM. It is a PTM, and for the glycocins a very important one.

I think they have shown that PalA, Hyp1 and Hyp 2 are indeed modified by a hexose, and they have identified this hexose as glucose making PalA a sublancin-like glycocin. Firstly, the addition of Figure 6 is very helpful to the reader and helps with interpretation of the mass spectrometry results. The only problem is these are the average masses, and are referred to as [M+H]+ ions in all the mass spectra. If this was the case they are not correctly referred to on the spectra. I applaud the authors providing ESI spectra collected on a Q Exactive, but to be absolutely sure of the conclusions drawn they should be showing the monoisotopic mass of one of the characteristic ions of the compound. (Refer to Stepper at al 2011.) What is shown here, I think are deconvoluted spectra giving M, not M+H, but I can't be sure as no details have been given as to how the data have been processed, or indeed of the conditions used for data collection and fragmentation. This must be done before the paper can be accepted for publication.

Similarly, was the Voyager in reflectron or linear mode? And how was the data processed if the former. What was the mass range what were the details of data

collection?

There are several other glaring errors. All masses have the decimal point replaced with a comma making the masses nonsensical.

Author's answer:

We agree that disulfide bond formation is a PTM. This statement is now corrected accordingly in the descriptions of all Supplementary Figures. In addition, we have replaced decimal separators to the points in the text.

All the masses (monoisotopic and average) are now recalculated as $[M+H]^+$ ions and added to the new Supplementary Fig 8. The MALDI-TOF-MS spectra are represented as $[M+H]^+$ ions now. We have removed all the text about the average masses from the descriptions of Supplementary Figures representing LC-ESI-MS spectra. As the reviewer suggested before, to be absolutely sure about the masses of peptides and conclusions we make, now we focus only on the monoisotopic masses. We admit that our evaluations and interpretations of average masses in the LC-ESI-MS spectra were not correct and we thank the referee for pointing this out.

I am happy with these changes

New figures of the LC-ESI-MS spectra represented as $[M+H]^+$, and also as $[M+H]^{+7}$ or $[M+H]^{+10}$ ions, are included now. This is due to the deconvolution algorithm did not work very well for larger peptides/proteins and the software could not recognize the first isotope correctly because the intensity was low.

Mentioned above you do not have to show deconvoluted spectra. It is possible to calculate the mass of the parent ion if you have the monoisotoipc mass and charge of a single ion .

More details about conditions of mass spectrometry analysis are now provided in Supplementary Information file in the section of *Mass spectrometry analysis.*

In addition to the new Supplementary Figures of LC-ESI-MS analysis that confirm the presence of disulfide bonds in the peptides, we note that these bonds were also confirmed by an alternative method – reduction/alkylation of Cys prior the MALDI-TOF-MS analysis. These results are represented in Supplementary Tables 2, 3 and 4. However, the fact that reducing agents abolish the activity of our product is convincing evidence that shows that the disulfides are there and necessary for activity, as they are also in other glycocins.

I am happy with these revisions.

Reviewer:

Supplementary Figure 7. The difference of 3 amu (+2.21 Da) between the glycosylated and non-glycosylated pre-palA-His-Glc –(2 SS) is put down as machine error. It could equally represent the formation of only 1 disulfide or even none.

Furthermore, the adducts listed at the bottom of the figure have the wrong masses:

Na +23, Met oxidation +16, methylation +14.

Author's answer:

The masses of adducts in the figures have been corrected.

Supplementary Figure 7 represented analysis by MALDI-TOF-MS. The accuracy for the machine is >0.05% of sample mass. This resolution is not good enough for disulfide bond confirmation. MALDI-TOF-MS was used as an initial screening and inspection method to observe the glycosylation and the leader cleavage of the peptides. The disulfide bonds in the peptides were confirmed by reduction/alkylation of Cys prior the MALDI-TOF-MS analysis, these results are represented in Supplementary Tables 2, 3 and 4. Using this method similar mass variations were observed as in the analysis of sublancin (Journal of American Chemical Society, 133(41):16394-7, 2011.).

Supplementary figure 7 is now a DNA gel.

Reviewer:

Supplementary Figure 8. Repeat of Figure 7 on a more accurate ESI instrument. The average mass of pre-palA-His is given as 7877.93, yet the mass observed is 7874.48. This gives a mass difference of 3.45, which although near to 4 is not accurate. In (b) the addition of 162.05 is correct, showing PalA is glycosylated with a hexose. Fig 8c shows the correct mass for the mature PalA. However, no mention is made of the large peak at about 8500 amu?

Author's answer:

The large peak in the Figure 8c represents unknown compound with a mass of approximately 8465.19 Da. It could be a residual peptide in the column, which could appear there from a previous run and analysis on the LC-ESI-MS machine. Nevertheless, our interest is the peak with a mass of approximately 5296.24 Da. We note that these data are now represented in new Supplementary Fig. 10 with isotopic masses, and it confirms the presence of disulfide bonds in the peptides. We note that these bonds were also confirmed by an alternative method – reduction/alkylation of Cys prior the MALDI-TOF-MS analysis. These results are represented in Supplementary Tables 2, 3 and 4. However, the fact that reducing agents abolish the activity of our product is convincing evidence that shows that the disulfides are there and necessary for activity, as they are also in other glycocins.

Supplementary Figure 8 is now a list of the peptide sequences and their theoretical average and monoisotopic masses.

Reviewer:

Supplementary Figure 9. Again the observed average mass of pre-His-Xa-palA-Glc-is calculated to be 8644.59, on the TOF (should it be 8645.59 for [M+H]+) which is the average mass less 4H for the disulfide bonds. What is observed is a mass of 8516 said to be the expected average mass less the mass of the first Met, 131.2 amu. However the mass difference is 128.55 amu. Clearly not methionine unless the 2 disulfide bonds are not formed (more likely).

Author's answer:

Supplementary Figures 9a and 9b represent analysis by MALDI-TOF-MS. As mentioned before,

the accuracy for the machine is >0.05% of sample mass. The observed mass difference of +2.65 Da should be tolerated as the machine error. Using this method similar mass variations were observed in analysis of sublancin (Journal of American Chemical Society, 133(41):16394-7, 2011.).

Analysis represented in Supplementary Figure 9a was performed to select elution fractions with the glycosylated precursor peptide (pre-His-Xa-PalA-Glc) after RP-HPLC analysis, and to inspect the purity of the peptide for a subsequent experiments. Importantly, the final product after the leader cleavage – mature pallidocin (PalA-Glc, without His-tag), was analyzed by LC-ESI-MS (Supplementary Figure 9c) and the obtained ion mass matched the expected monoisotopic mass of mature pallidocin with two disulfide bonds. This figure is now represented in the new Supplementary Figure 11.

I have already made comments about this. Figure 11a is made redundant by figure 10b and figure 11b by figure 11c. Figures 10a, 10b and 11 c give good evidence and are all that are required.

Reviewer:

Supplementary Figure 9 (b) and (c), are said to represent the mature pallidocin molecule and both spectra have a peak at ~4221 amu. Yet the mass given for the peptide in Figure 6 is 5137.77. Bearing in mind that including the tag, there are 48 amino acids and using the average amino acid mass of 110, we would expect a mass of ~ 5280, so 5137.77 seems reasonable. If the peptide loses 4 amu because of the formation of 2 disulfide bonds, then gains 162.05 due to glycosylation, we would expect 5295.77 amu. If the N-terminal Met is missing, the expected mass would be 5164.57. If the tag does not include a GG, there would be a further loss of 114 to give ~5050. Clearly none of these masses match the observed mass of 4221.

Author's answer:

The reviewer is confused between peptides PalA-His-Glc (mature pallidocin core peptide with His-tag in C-terminus, $[M+H]^+$ avg. 5296.89 Da) and PalA-Glc (mature pallidocin core peptide without His-tag, $[M+H]^+$ avg. 4222.8 Da). The glycosylated precursor peptide pre-His-Xa-PalA-Glc has a His-tag in the N-terminus, which was later cleaved together with the leader sequence. The synthesis of pallidocin in this approach resulted in PalA-Glc – the mature pallidocin without His-tag (4222.8 Da); it is the same peptide as been derived after the expression of the whole pallidocin biosynthetic gene cluster. This figure is now represented in the new Supplementary Figure 11.

Amino acid sequences of PalA-His and pre-His-Xa-PalA are represented in the Supplementary Figure 8. This figure also indicates the positions of His-tag, leader and core peptide sequences.

I don't think I was confused, but the new monoisotopic masses calculated from the Q Exactive orbi provides the appropriate evidence

Reviewer:

Supplementary Figure 11. The authors checked their system using the sublancin model using MALDI. The mass of 6834.81 is claimed to represent pre-SunA-his with 2 methionine oxidations. Figure 6 shows that pre-SunA-His does not have 2 methionines, only a single one. Again there is a difference of 2 amu between the observed and calculated masses of the unmodified pre-sunA-His.

Author's answer:

Supplementary Figure 11 represented analysis by MALDI-TOF-MS. As mentioned before, the accuracy for the machine is >0.05% of sample mass. The mass of main peak ($[M+H]^+$ – 6802.81 Da) in the Supplementary Figure 11a match the mass of pre-SunA-His with two disulfide bonds ($[M+H]^+$ avg. – 6801.84 Da) with variation of +0.97 Da, which can be accepted as the machine error.

The mass of the second lower intensity peak ($[M+H]^+$ – 6834.54 Da) match the mass of the same peptide with adducts. The difference of +32.70 Da between theoretical ($[M+H]^+$ avg. – 6801.84 Da) and observed ($[M+H]^+$ – 6834.54 Da) masses might be caused by: water (+18 Da) and methionine oxidation (+16 Da) including machine error (-1.30 Da). The reviewer is correct, the pre-SunA-His has only one Met, so there can be only one Met oxidation. It is complicated to determine precise masses of the peptides because of mass variations introduced due to instrument inaccuracy or adducts in the peptides. These data are represented in the new Supplementary Fig. 14.

This figure is not necessary in terms of the more accurate maases from the Q-Exactive. I would remove it as all it tells you is that you have a modification. Figure 15 provides the real evidence from this experiment. Again the monoisotopic mass can be calculated from the monoisotopic 7+ ion as 6797.3 compared to the theoretical mass of 6797.26. Delete the NOTE.

Reviewer:

Supplementary Figure 12. While these ESI spectra convincingly show the modification of SunA by PalS there is once more doubt about the formation of disulfides with a mass difference of 3 rather than the 4 expected for the formation of 2 disulfide bonds.

Author's answer:

As the reviewer suggested above, to be absolutely sure about the masses of peptides and conclusions we make, we focus only on monoisotopic masses, now. We admit that our evaluations and interpretations of average masses in LC-ESI-MS spectra were incorrect. The new Supplementary Fig. 15 with isotopic masses confirms the presence of disulfide bonds.

See my comment above. I am happy with this

Reviewer:

Supplementary Figure 13. The mass of 7934.69 represents a mass difference of 18, not 23 amu thus the addition of water rather than Na. Again, justifying the increase

in mass of 16 as Met oxidation without evidence is risky. Sequencing using MSMS would clarify these modifications.

Author's answer:

Supplementary Fig. 13 represented analysis by MALDI-TOF-MS. The reviewer is correct, the mass difference between theoretical ($[M+H]^+$ - 7917.14 Da) and observed ($[M+H]^+$ - 7934.69 Da) masses is +17.55 Da. It might represent the addition of water (+18 Da) with a variation (-0.45 Da). On the other hand, the observed mass matches the peptide with two disulfide bonds (-4 Da) and sodium adduct (+22 Da) with a variation (-0.45 Da). As mentioned before, the accuracy for the machine is >0.05% of sample mass, and using this method it is complicated to accurately determine the masses of peptides. These data are now represented in new Supplementary Fig. 16.

Supplementary figure 16 is redundant and contains a lot of inaccurate masses that have been explained away by not very convincing reasons. It has been superseded by Figure 17 and should be removed

Reviewer:

Supplementary Figure 14. Repeat of the analysis in Fig 13 confirms the addition of a hexose, but indicates the presence of only a single disulfide (7916-7914) and (8076-8074).

Author's answer:

As the reviewer suggested above, to be absolutely sure about the masses of peptides and conclusions we make, we focus only on monoisotopic masses, now. We admit that our evaluations and interpretations of average masses in LC-ESI-MS spectra were incorrect. The new Supplementary Fig. 17 with monoisotopic masses confirms the presence of disulfide bonds.

Moreover, two disulfide bonds were confirmed by an alternative method: reduction/alkylation of Cys prior the MALDI-TOF analysis, these results are represented in Supplementary Table 3.

Happy with figure 17 and Table 3

Reviewer:

Comment 9. Figure 2 does not reflect the assignment of secondary structure, and it

is not an output from PSIPRED. As far as I can see it is modelled on sequence homology to sublancin. However it is good to see a far UV CD spectrum.

Author's answer:

The Figure 2 is corrected now, and represents secondary structure of pallidocin, which is based and modeled according to PSIPRED calculations.

I would like to suggest that you amend both the caption and the text be amended to read "proposed structures of xxxxxxxx based on Psipred predictions from the sequence and the known tertiary structure of Sublancin 168 and GccF".

Psipred tells you where the helices are, but not the connectivity of the disulfides .

Reviewer:

Comment 10. Questions 10-12 in rebuttal 1. Points raised have been corrected.

Author's answer:

Reviewer is satisfied with improvements.

Reviewer:

Comment 11. Questions 13 in rebuttal 1.I take the authors point. But the question of the number of disulfide present can be accurately determined using monoisotopic mass spectrometry. However the reduction/alkylation experiments reported in Supplementary tables 2-4 confirm the presence of disulfide bonds.

Author's answer:

The new Supplementary Figures of LC-ESI-MS analysis represents monoisotopic masses, which confirms the presence of disulfide bonds in the peptides, now. In addition, two disulfide bonds in all the peptides were confirmed by an alternative method: reduction/alkylation of Cys prior the MALDI-TOF analysis, these results are represented in Supplementary Tables 2, 3 and 4. Moreover, the latter results showed the importance of these bonds for antibacterial activity.

Happy with these tables

Reviewer:

Comment 12. Questions 14-15 in rebuttal 1 have been addressed and I am happy with the changes.

Author's answer:

Reviewer is satisfied with improvements.

Reviewer:

Comment 13. Question 16 in rebuttal 1. The article still needs a very careful edit as there are many grammatical errors, especially in the supplementary info.

Author's answer:

Editing and careful check up was performed.
It is much better

Errors to be corrected

Page 10 line 166 Should be supplementary Figure 9d and 10c . There is no 10d

Page 10 Line 176. Why on earth were these masses not measured on the Q-Exactive. These excuses for mass accuracy weaken the paper in my opinion.

Page 14 line 236. Remove Fig 2

Page 14 Line 238 ….by two disulfide bonds giving rise to the predicted structures for Hyp1 and Hyp2 (Fig 2)

Page 15 Line 256 with the two new genes would be better with the names of the genes.

Page 15, Line 260 . 'was expresed in *E. coli* without *palS* co-expression.' The recombinant hyp1-His core peptide'

Page 16 Line 280. A small nigle. I note there is no evidence presented for these clains in the supplementary information.

Page 17 Line 316 Reads better as 'In vitro studies on the glycosylation of the sublancin precursor showed that S-glycosyltransferase…………'

Reviewer:

Overall the authors have answered all my concerns. The manuscript reads much better but I recommend that they remove all the MALDI spectra as they are now redundant. Absolute proof of the PTMs is given by the ESI Q-Exactive spectra. I recommend that the masses of the various protein products can be calculated from a monoisotopic ion as indicated above.

Reviewer #2 (Remarks to the Author)

Reviewer:

The authors have addressed the comments made by the reviewers. However, I do have a couple of suggestions:

Comment 14. On several occasions instead of the word "cytoplasm" the authors used "citoplasm" not sure if its a spelling error or regional use.

Author's answer:

Corrected.

Reviewer:

Comment 15. Upon the addition of the new data on the antimicrobial activity of modified Hyp1 and Hyp2, the authors did not include within the discussion section as to why Hyp2 does not show antimicrobial activity but Hyp1 does. I think it will be interesting if the authors explore the differences between these peptides that might explain the differences in biological activities.

Author's answer:

Changes in the Discussion section of manuscript:

All *sublancin*-type glycocins, including novel pallidocin, Hyp1 and glycosylated core peptide Hyp2, have relatively rich content of hydrophobic residues in N-terminus, and charged residues in C-terminus. Comparing the core peptides of glycocins, the Hyp2 has relatively long C-terminus "tail", not characteristic to other sublancin-type glycocins, and is relatively rich in charged residues (Glu20, Arg21, Arg22) in the interhelical loop (Fig. 2). These two features or one of them might be the reason why glycosylated Hyp2 core peptide and precursor did not have antibacterial activity against the strains tested. We cannot exclude the possibility that it has a different spectrum of activity, too. This could be the subject for future research on glycocins.

Reviewer:

Comment 16. On the figure legend of several ms spectra, the authors used the phrase" within an error" and I think it should read "within error".

Author's answer:

Corrected.

Reviewer:

As it is written the work presented here seems interesting and in my opinion will be of broad interest to the RiPP and natural product community.

Reviewer #3 (Remarks to the Author)

Reviewer:
My concerns have been adequately addressed. The manuscript reads much better now.

REVIEWERS' COMMENTS:

Reviewer #1 (Remarks to the Author):

1. Overall the authors have answered all my concerns. The manuscript reads much better now and only needs a few minor modifications for publication as shown in the attached modified rebuttal. I recommend that they remove all the MALDI spectra as they are now redundant. Absolute proof of the PTMs is given by the added ESI Q-Exactive spectra. I recommend that the masses of the various protein products can be calculated from a monoisotopic ion when deconvoluted spectra cannot b calulaed.

Authors' answer:

As the reviewer advised, we have removed all figures representing the MALDI-TOF-MS data.

Other errors to be corrected are as follows:

2. Page 10 line 166 Should be supplementary Figure 9d and 10c . There is no 10d.

Authors' answer:

Corrected. Supplementary Figure 9d representing MALDI spectra is removed. Following editorial requests, Supplementary Figure 10c renamed to Supplementary Figure 16.

3. Page 10 Line 176. Why on earth were these masses not measured on the Q-Exactive. These excuses for mass accuracy weaken the paper in my opinion.

Authors' answer:

At the time when these experiments were performed, we expected that MALDI would be sufficient to prove the presence or absence of disulfide bonds, and in fact it still is. At the moment we have no longer possibility to repeat the experiments by the Q-Exactive, but we show the presence of disulfides also convincingly by the alkylation experiments.

4. Page 14 line 236. Remove Fig 2.

Authors' answer:

Corrected. Page 14 Line 229.

5. Page 14 Line 238 add .... by two disulfide bonds giving rise to the predicted structures for Hyp1 and Hyp2 (Fig 2).

Authors' answer:

Corrected. Page 14 Line 231.

6. Page 15 Line 256 with 'the two new genes' replace with with the names of the genes.

Authors' answer:

Corrected. Page 15 Line 249.

7. Page 15, Line 260 . 'was expresed in E. coli without palS co-expression. The recombinant hyp1-His core peptide'

Authors' answer:

Corrected. Page 15 Line 253.

8. Page 16 Line 280. A small nigle. I note there is no evidence presented for these clains in the supplementary information.

Authors' answer:

New Supplementary Figure 28, which represents the evidence, is now included in the Supplementary Information file.

9. Page 17 Line 316 Reads better as 'In vitro studies on the glycosylation of the sublancin precursor showed that S-glycosyltransferase………….'

Authors' answer:

Corrected. Page 17 Line 307.
